# Identification of Opportunistic Pathogens on the Skin of Salamanders for Use as Molecular Targets of a De Novo Design of Multitarget Anti‐*Bd* Proteins

**DOI:** 10.1155/ijm/5903624

**Published:** 2026-04-20

**Authors:** Jimena Ramírez-Villarreal, Roberto Álvarez-Martínez

**Affiliations:** ^1^ Facultad de Ciencias Naturales, Doctorado en Ciencias Biológicas, Laboratorio de Biología Cuantitativa y Sistemas Complejos, Carr. a Chichimequillas S/N, Universidad Autónoma de Querétaro, Ejido Bolaños, Santiago de Querétaro, Querétaro, 76140, Mexico, uaq.mx

**Keywords:** amphibian, bacteriome, chytridiomycosis, diseases, microbiota, treatments

## Abstract

**Objectives:**

Chytridiomycosis, caused by *Batrachochytrium dendrobatidis* (Bd), is one of the most devastating fungal diseases affecting amphibians worldwide. This study aimed to design an in silico protein‐based treatment targeting Bd and pathogenic bacteria associated with the skin microbiota of four Bd‐infected salamander species.

**Methods:**

Pathogenic and opportunistic microorganisms present in the skin microbiota were identified, and those common across all four species were selected for further analysis. A shared molecular target among the common bacterial pathogens was identified, and structural prediction models were used to search for compatible amino acid sequences. Short peptide sequences with previously predicted biological functions were retrieved from a curated database and evaluated for their antimicrobial and antifungal properties, followed by molecular docking assays to select the most promising candidates.

**Results:**

A single protein‐based molecule with therapeutic potential against both Bd and the identified pathogenic and opportunistic bacteria within the skin microbiota of Bd‐infected salamanders was confirmed across all species studied.

**Conclusions:**

These findings highlight the importance of considering both the fungal pathogen and the associated microbiota when developing treatments for chytridiomycosis, though experimental validation remains necessary before potential application in wildlife conservation programs.

## 1. Introduction

In fungal diseases of wildlife, certain environmental factors have favored the proliferation of opportunistic pathogens [1–3]. Examples, including white‐nose syndrome in bats and *Batrachochytrium dendrobatidis* (*Bd*) disease in amphibians, illustrate the seriousness of the problem [4–6]. The chytridiomycosis skin infection caused by *Batrachochytrium dendrobatidis* (*Bd*) and *Batrachochytrium salamandrivorans* (*Bsal*) has led to the extinction of numerous amphibian species [7, 8]. Although this infection has been studied in various parts of the world, there is still no treatment that is effective for all species and life stages of susceptible animals. Additionally, many of the chemical treatments tested have undesirable side effects on juveniles and larvae [9]. The main reason that chemical treatments have not worked in their entirety is that all animals at risk of *Bd* differ significantly in the microbiota present on their skin [10, 11]; these differences are due to the biotic and abiotic context in which they live and their terrestrial [12] or aquatic [13–15] habits, as well as other factors such as sex [16], temperature [17], age [2], and even their susceptibility to chronic limb lesions [18]. Microbiota have been implicated in altering the uptake, distribution, and effects of various treatments [19–26]. The same may be true for *Bd* and amphibians, but this has not been studied in detail. However, there is evidence that dysbiosis of the amphibian skin microbiota may induce changes in limb regeneration [27–30].

Microorganisms play a crucial role in various aspects of host health, particularly in infectious processes [31–34]. Many pathogenic microorganisms can form interactions with other fungi or bacteria to benefit from them, gaining various advantages, such as nutrients, space, and resistance to drugs. They may even induce better colonization of mucosal surfaces through the formation of biofilms [35–40]. It has also been shown that these interactions may not be mutually beneficial [41–43]. An example that has recently been studied is that certain microorganisms may produce secondary metabolites or even directly disrupt the development and growth of other pathogenic microorganisms [44]. All of these positive and negative interactions may play an important role in animal health and infection processes, as it has also been shown that chronic infections and previous dysbiosis can alter the host’s initial microbiota and induce increased susceptibility to other pathogens and even have a synergistic effect on subsequent infections [45]. All of these interactions have not been studied in amphibians.

The microbiota can be regulated by the host animal and, in some cases, by specific communities of microorganisms within the microbiota itself. There is evidence that species such as *Janthinobacterium lividum* may have probiotic effects on amphibian skin [46–48]. These effects may regulate the colonization of pathogenic and opportunistic microorganisms, including *Bd*. Probiotic treatments have been tested on some amphibians, with some cases yielding successful results, while in other cases, the expected effect was not observed [49, 50]. Various chemical treatments have also been tried, such as ivermectin, voriconazole, and itraconazole, which are drugs known for their antifungal and antiparasitic effects; however, avoiding microbial resistance to these compounds by *Bd* and other microorganisms that make up the animal’s microbiota has not yet been achieved, and the effects seen do not apply to all animals susceptible to *Bd* [51–56]. In addition to the undesirable side effects of its use in natural areas, it is due to the contamination of water and soil over long periods, which affects pollinators and invertebrates [57]. The inappropriate and excessive use of these types of chemicals can induce resistance and multiresistance in other microorganisms or parasites due to the permanent selection pressure generated by these treatments released into the environment [58].

It is true that frogs are more severely affected by the infection. However, many treatments are currently being tested on frogs, including heat therapy [59] and various chemical compounds [54, 60, 61]. Some of these treatments have shown favorable results, particularly heat therapy [59]. Conversely, salamanders are less tolerant of high temperatures and tend to inhabit cool environments. In fact, heat has been documented to be lethal to this type of animal, so heat therapy is ineffective [62–67]. Favorable effects of chemical therapies have only been studied in a few species, and they often require synergy with other treatments [54, 60–68]. Excessive use of these options can complicate treatment by inducing resistance in other pathogens that may be present [69].

Several interesting approaches to attacking the fungus have been developed to date. Studies have examined frog skin to identify and isolate antimicrobial peptides (AMPs) that are effective against Bd. These peptides have been applied directly against Bd with varying degrees of success [70]. Researchers have also “improved” these peptides to increase their efficiency [71]. Libraries of molecules that have been tested and approved for other infections have also been used against Bd [54, 59–61]. Similarly, inhibitors of key Bd enzymes that disrupt cell wall synthesis and/or metabolic processes have been designed [72]. New molecular targets for designing treatments are also being sought [73]. These different approaches have helped determine effective strategies against the fungus and decide which path to follow or which new strategies to try.

Designing specific proteins as a treatment is a strategy that has not been widely explored. It is a different approach from those that have been studied. This approach takes into account that the infection continues to affect both types of animals, and there is still no definitive treatment. This work proposes an *in silico* methodological process for designing a treatment against Bd that could be used to develop drugs for other infections. The reported protein design targets pathogenic bacteria present on the skin of infected animals and is independent of a Bd strain capable of infecting frogs and salamanders due to a specific antifungal sequence [74].

Recently, therapies such as exposing the animals to certain temperatures have been tested; this therapy has been called “frog saunas,” which, although a good idea to support amphibian conservation in the face of the pathogen threat, still has some limitations, such as the need for materials or equipment to be installed in the areas where these endangered animals are found [75]. They also need to be sufficient for treatment to be applied on a large scale, and there are also records of reinfection of individuals [76]. In addition, chytrid fungi, such as *Bd*, have been shown to produce barriers and enzymes that protect their integrity and ensure survival at high temperatures and in the presence of chemical treatments [77–80].

The development of treatments for these diseases is a significant challenge. It is crucial to consider not only the pathogen but also the host microbiota, which can help enhance immune defenses [14, 34, 81]. Studies in amphibians have shown that the skin microbiome may play a key role in resistance to fungal infections [82, 83]. However, the interactions between these microorganisms and pathogens require further exploration to develop more effective treatments that do not disrupt the microbiological balance in the animal’s skin. Conservation of amphibians is critical to ecosystems because they regulate insect populations, including pests that affect human health as vectors of important diseases such as dengue, Zika, and malaria [84, 85].

The emergence of new microorganisms that cause severe infections in amphibians has increased the need for more rapid development of new treatments [86, 87]. This work focuses on proposing a treatment design for the potential mitigation of *Bd* based on the exploitation of patterns of pathogenic and opportunistic microorganisms present in the skin of salamanders with *Bd* infection that may be involved in enhancing *Bd* colonization in the skin of susceptible animals. Therefore, the treatment proposed here aims to control the proliferation of *Bd* and the pathogenic and opportunistic microorganisms present on the skin. For this purpose, we will utilize data on the skin microbiota of salamanders in which *Bd* has been detected, for which information has already been published and registered in Bioproject, as well as advanced design tools freely available for a comprehensive bioinformatics approach. This type of specifically designed treatment could not only help address fungal infections but also serve as a pathway for the development of new and targeted therapies.

## 2. Materials and Methods

### 2.1. Compilation of the Sequences of Diseased and Healthy Animals

We extensively searched in PUBMED for projects that would study the microbiota of salamanders and identify *Batrachochytrium dendrobatidis* by qPCR or PCR. Additionally, they were required to register their samples in BIOPROJECT and include the corresponding number in their manuscript. We analyzed the databases associated with these projects to verify that each SRR sample had a corresponding location record in coordinates and that it indicated whether the sample corresponded to an animal with positive or negative *Bd* detection. There are numerous studies on the microbiota of salamanders; however, studies that detect infections are scarce. Therefore, we found only a limited number of articles with *Bd-positive* samples. The data belong to at least two families of Caudata: Salamandridae and Ambystomatidae. The genera studied were *Ambystoma altamirani*, *Notophthalmus viridescens*, *Desmognathus monticola,* and *Eurycea bislineata*. All locations are situated in two areas of Virginia, USA: Brightwood and Front Royal.

### 2.2. Pathogen Identification

Sequences of interest were selected and downloaded using the SRA toolkit in bash from the following Bioprojects: PRJNA819099 [87] and PRJNA659464 [15]. Using the SRA toolkit, the sequences were downloaded from the public database and converted to the correct FASTQ format using a bash script for further processing. For the identification of opportunistic pathogens and pathogens, a 16sPIP detection program was used, which performs pathogen detection within a list of 155 pathogens of clinical importance [88]. Results were downloaded from the program in PDF format for subsequent extraction of data into Excel spreadsheets in CSV file format. With the data already in the tables, a presence–absence matrix was generated for analysis using the R programming language. In addition, different Venn diagrams were generated with the lists to determine the differences in composition by salamander species and by *Bd*/no *Bd* condition.

### 2.3. Multiple Alignment

For the four important microorganisms, the same transmembrane protein of about 300 amino acids in sequence length was found, characterized as a transport protein MFS (Major facilitator superfamily), one of the proteins belonging to two large families of transmembrane transporters present in the orders Bacteria, Archaea, and Eukarya, which can have functions of solute uniport, cation and solute symport, cation/solute antiport, solute/solute, and can be input or output rectifying. Furthermore, they have been found to play an important role in the transport of sugars, inositols, oligosaccharides, amino acids, drugs, and even metabolites [89]. MUSCLE was used as a tool to perform an analysis of the four proteins, looking for similarities as well as conserved regions between the four different microorganisms. In addition, a multiple BLAST alignment was performed with each of the MFS protein sequences to search for microorganisms with partial or complete homology sequences.

### 2.4. Selection of the Target Site

We searched for proteins that preferably had a crystallographic structure, but only the sequences were available. To determine the structure of the target proteins, we utilized the trRosetta program and selected models with a high‐quality TM score greater than 0.8 in all cases. The sequences belonging to the extracellular region of the transport proteins were analyzed using SWISS‐MODEL [90] to determine the membrane location of the proteins. With the help of Pymol, the sequences of the extracellular and pore regions of the transporters were studied. The sequences were extracted, and the possible amino acids with chemical properties that could interact with other molecules were identified, including aromatic amino acids (W, Y, C) with the ability to form sulfhydryl or disulfide bridges. A, G, L, I, T, and M amino acids with specific amphipathic, hydrophilic, and hydrophobic characteristics interact with other molecules. In addition, sequences rich in amino acids, such as arginine (R) and tryptophan (W), which are known to have antimicrobial effects, were considered. A comparison of the structural models obtained from I‐TASSER and trRosetta with those obtained from AlphaFold revealed significant similarities (see Supporting File 1).

### 2.5. Sequence Construction

Several freely available tools were used to construct a sequence with antimicrobial and antifungal properties: Registry of Standard Biological Parts‐iGEM, CIBV (Linker Database), CAMP, and DRAMP [91], which are databases of sequences with natural or synthetic antimicrobial and antifungal prediction effect, and the arginine and tryptophan (R and W) amino acid recommendations from [92]. Twelve random sequences were constructed based on information extracted from the databases, literature recommendations, and links between sequences in the databases. Each was modeled using trRosettaX‐Single, the preferred method for de novo protein modeling that does not rely on templates or homologous sequence searches. A total of 12 structures were obtained, with different TM score quality values, considering that these are typically very low due to the de novo modeling of the protein.

### 2.6. General Tests

The CAMPr4 program [93] was used to analyze the sequences and determine whether or not they had potential antimicrobial activity. These scores are normalized from 0 to 1, and those that were greater than 0.5 according to the program specifications were considered candidates for further testing. Testing for synthetic and natural AMP potential was performed. The Antifp program was used to determine the physicochemical properties of each sequence, such as hydrophobicity, amphipathicity, charge, isoelectric point, and molecular weight, and to assess whether the sequences exhibited any antifungal potential effect. BIOFIN [94] was used to predict whether the constructs had an antibiofilm potential activity. HemoPI [95] was used to determine whether the constructed proteins exhibited hemolytic activity. ProtParam was used to investigate the half‐life of the constructs in various models, including reticulocytes, yeast, and *E*. *coli* [96]. Additionally, a folding quality test was conducted using the QMEANDisCo program, which yielded unsatisfactory results due to its reliance on templates for quality analysis [97].

### 2.7. Choosing the Best Design

To determine which of the constructs is the best choice as an antimicrobial based on the binding of the designed sequence and its molecular target, a molecular docking test was performed using the HEX loria program [98]. The six best‐designed sequences were analyzed based on the results of the above tests. Each of these designed structures was named BD_ plus the generated sequence number, and each BD_# molecule was tested with each of the four molecular targets to ensure its potential effect in the four different microorganisms. A comparison of the structural models obtained from HEX loria vs. HADDOCK with the structural models from AlphaFold revealed significant structural similarities and molecular docking matches (see Supporting File 1).

### 2.8. Antifungal Part Design

It was found that [99] developed a formula for designing synthetic peptides against fungi. This formula was used to design the sequence. The APD3 Antimicrobial Peptide Database can also be used as a reference at the address: https://aps.unmc.edu/. Two different approaches can be followed: (1) enhance the effect of molecules already described and tested in the literature and generate a chimeric protein from them, or (2) take into account aspects such as charge, hydrophobicity, hydrophilicity, pI, solubility, hydropathicity, amphipathicity, and, above all, the structure of the amino acids to design an ideal sequence, and finally, (3) follow formulas or search databases for the sequence. The same design process was performed to link the sequence with the predicted antifungal effects to Antifp, and links from the iGEM parts registry of sequences were used to link to the main BD_# sequences. Quality and other tests were performed with this separate sequence in the same programs. To ensure the effect of each of the protein parts separately. Finally, to ensure recognition of this model concerning its molecular target, a molecular docking analysis was also performed using HEX loria.

## 3. Results

From the entire database of sequences collected for skin microbiota analysis of healthy and diseased animals, the sequences of those amphibians with disease detected by PCR were selected for the development of a potential treatment. The animals analyzed correspond to the following genera and species, along with the number of sequences studied: *Desmognathus monticola* (5 sequences), *Notophthalmus viridescens* (12 sequences), *Eurycea bislineata* (2 sequences), and *Ambystoma altamirani* (29 sequences). The raw sequences were analyzed using the 16S rRNA Pip server. This server identifies sequences of pathogenic or opportunistic microorganisms within each FASTQ file of each processed animal. The results of this step were PDF files containing a list of the pathogenic microorganisms identified; for each animal, variations were observed in the number and identity of the microorganisms present (see complete list in Supporting Information Table S1). Bacterial identification was performed at the genus level based on the 16S rRNA sequence analysis; species‐level assignments should be considered putative due to the inherent limitations of this approach in wildlife microbiome studies.

When comparing salamanders of different species, we identified 13 species of common pathogenic bacteria as being present in all salamander species: *Escherichia coli, Yersinia pseudotuberculosis, Aeromonas hydrophila, Enterobacter aerogenes, Serratia marcescens, Enterobacter cloacae, Yersinia pestis, Pseudomonas aeruginosa, Citrobacter freundii, Edwardsiella tarda, Afipia broomeae, Acinetobacter lwoffii*, and *Klebsiella pneumoniae*. Some of the bacteria (common to all salamanders) used to generate the treatment design were selected based on these results, as in the case of *Edwardsiella tarda* and *Yersinia pestis*. *Acinetobacter baumannii* and *Vibrio fluvialis* were selected due to their high presence in the comparisons within the samples of the same salamander species, as well as the high values of proportions of readings found in the raw files analyzed (see Supporting Information S1–S6 and Table S1 y S2).

The following table summarizes the results obtained from the 16S rRNA Internal Transcribed Spacer (ITS) PCR program for identifying pathogenic bacteria, performed using the raw FASTQ files from animals of the genera *Ambystoma*, *Desmognathus*, *Eurycea*, and *Notophthalmus*. In the first column, we can see the names of the bacteria; in the next, the number of reads that matched the sequence of the bacteria to be detected and the percentage of those same reads (see Supporting Information S1–S6 and Table S1 y S2).

**FIGURE 1:**
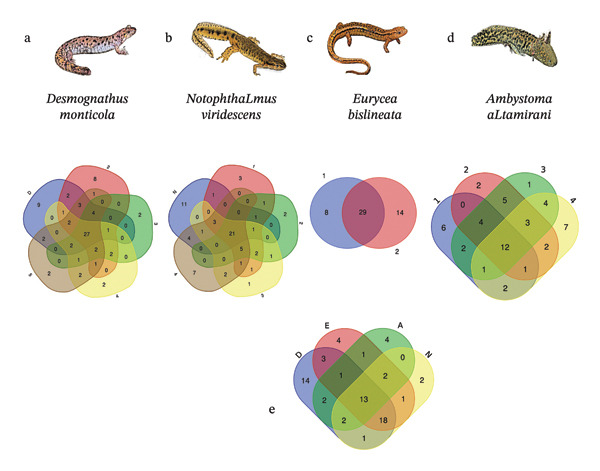
Differences in the microbial composition between groups. The Venn diagram is shown for each salamander species. (a) The results of the number of common and individual microorganisms for each of the *Desmognathus monticola* samples (5 animals in total). (b) The differences in the microbial composition of the samples in *Notophthalmus viridescens* (5 animals sampled in total). (c) *Eurycea bislineata* (2 animals sampled in total). (d) Differences in *Ambystoma altamirani* (4 animals sampled in total). And (e) shows the comparison between each species D (*Desmognathus*), E (*Eurycea*), A (*Ambystoma*), and N (*Notophthalmus*).

The genera and species with the highest number of sequence matches among the salamanders are *Vibrio fluvialis*, *Acinetobacter baumannii*, *Yersinia pestis*, and *Edwardsiella tarda*, with a higher percentage of values identified within each sample and animal (see Figure 1). Complete lists for each sample are available in the Supporting Information Table S1 of this article. All of these results were then analyzed by name in the PUBMED search bar to find articles with information on the characteristics of each pathogen and the diseases with which they are directly or indirectly associated.

For this purpose, three specific molecular targets were proposed in the opportunistic bacteria present: the cell wall, which is known to have a net negative charge, and a third target in a specific and functional protein of the membrane. Based on the above, for each genus and species identified, a search was performed in NCBI in the protein section or in the structures section to locate proteins with conserved vital functions. Proteins with a previously published crystallographic structure in the database can be used directly by downloading the PDB file for subsequent analysis. The same procedure was followed for *Bd*. In this case, however, no membrane proteins with already identified structures were found, so we continued to search for sequences in the protein section. With the sequences in the FASTA format, structural models of each were generated using the trRosetta program [100] to design a treatment that could interact with these proteins (see Supporting Information S1–S6 and Table S1 y S2).

Four target microorganisms were found, belonging to the species *Vibrio fluvialis, Acinetobacter baumannii, Yersinia pestis,* and *Edwardsiella tarda*. A common molecular target for all of them is the transmembrane protein MSF. A multiple alignment was performed to determine whether the protein was homologous between the different species. Sequences that were significantly conserved within the transmembrane regions were searched for to ensure that the designed protein interacted with parts of the protein that could potentially be key to its function. In none of the cases were homologies found in the sequence, making it ideal for generating a unique complementary sequence for receptor–ligand binding in all cases. Therefore, for each of the proteins of interest, the amino acids present in the extracellular regions were studied in order to define a target sequence for the treatment to be designed (see Supporting Information S1–S6 and Table S1 y S2).

A total of 12 sequences have been designed, each with a section corresponding to a specific potential action. That is, each sequence must contain one or two segments that favor the binding of the engineered protein to surface targets such as the wall and membrane of the microorganism. On the other hand, it must also have a section that binds by “complementarity” to the specific membrane proteins in the bacteria. In addition, each section of the protein must have an intermediate sequence that serves as a spacer, facilitating the protein’s folding and thereby enhancing its stability. Therefore, short spacer sequences from the Registry of Standard Biological Parts (iGEM database) were also utilized (see Supporting Information S1–S6 and Table S1 y S2).

To ensure that each part of the sequence performs its potential effect both generally and specifically, various tests were performed to determine whether current bioinformatics tools would identify the engineered protein as antimicrobial or antifungal. For this purpose, a CAMPr4 database was used, which can identify sequences such as AMP (antimicrobial) or NAMP (nonantimicrobial). Within the same database, there are synthetic AMP sequences, that is, those designed in laboratories or naturally isolated from compounds found in nature or secreted by animals. Using these data, the program is responsible for identifying similarities between the amino acids of the sequence of interest and the data it stores (see Table 1). Of the 12 sequences designed, only half were named: BD_3, BD_4, BD_7, BD_8, BD_10, and BD_12 passed this first filter and were identified as synthetic AMPs. Nine natural AMPs were identified: BD_1, BD_2, BD_3, BD_7, BD_8, BD_9, BD_10, BD_11, and BD_12 (see Supporting Information and Table 2).

**TABLE 1:** Antimicrobial properties of the designed sequences.

ID	CAMPr4_RF (synthetic)		CAMPr4_SVM		CAMPr4_ANN		CAMPr4_RF (natural)		CAMPr4_SVM		CAMPr4_ANN	
	CLASS	AMP PROB	CLASS	AMP PROB	CLASS	AMP PROB	CLASS	AMP PROB	CLASS	AMP PROB	CLASS	AMP PROB
BD_1	NAMP	0.41	NAMP	0.39	NAMP	0.17	AMP	0.74	AMP	0.97	AMP	0.81
BD_2	NAMP	0.47	AMP	0.69	NAMP	0.38	AMP	0.71	AMP	0.99	AMP	0.85
BD_3	AMP	0.66	AMP	0.68	NAMP	0.4	AMP	0.61	AMP	0.96	NAMP	0.35
BD_4	AMP	0.55	AMP	0.7	NAMP	0.42	NAMP	0.44	AMP	0.63	NAMP	0.06
BD_5	NAMP	0.46	NAMP	0.44	NAMP	0.15	NAMP	0.48	AMP	0.53	NAMP	0.06
BD_6	NAMP	0.48	NAMP	0.35	NAMP	0.12	NAMP	0.46	AMP	0.53	NAMP	0.06
BD_7	AMP	0.99	AMP	0.98	AMP	0.99	AMP	0.77	AMP	1	NAMP	0.15
BD_8	AMP	0.98	AMP	0.97	AMP	0.99	AMP	0.66	AMP	0.99	NAMP	0.18
BD_9	NAMP	0.27	NAMP	0.15	NAMP	0.21	AMP	0.78	AMP	0.95	AMP	0.84
BD_10	AMP	0.89	AMP	0.99	AMP	0.97	AMP	0.84	AMP	0.56	AMP	0.88
BD_11	NAMP	0.14	NAMP	0.16	NAMP	0.03	AMP	0.52	AMP	0.95	NAMP	0.07
BD_12	AMP	0.96	AMP	0.97	AMP	0.99	AMP	0.66	AMP	1	NAMP	0.33

*Note:* In the following table, we can observe the results of the CAMPr4 program, which predicts whether a protein has a potential antimicrobial prediction activity (AMP) or not (NAMP). This prediction also applies to both designed proteins (synthetic) and protein sequences extracted from nature (natural). It has three different prediction methods using different prediction databases (_RF, _SVM, and _ANN). Probabilities above ≥ 0.5 are considered as AMPs.

**TABLE 2:** Antifungal properties.

ID	Score	Prediction	Hydrophobicity	Hydropathicity	Hydrophilicity	Amphipathicity	Charge	pI	Mol wt
BD_3	−0.03	NANTI	−0.16	−1	0.18	0.4	−1	4.95	3851.62
BD_4	0.013	NANTI	−0.51	−2	0.81	0.8	−1	4.95	2263.61
BD_5	0.04	NANTI	−0.51	−2	0.7	0.84	−1	5.06	2966.47
BD_11	−0.03	NANTI	−0.61	−2	1.77	0.84	−12	4.04	4580.04
BD_6	−0.02	NANTI	−0.5	−2	0.69	0.81	−1	5.06	3053.56
BD_9	−0.06	NANTI	0.09	0.44	−0.49	0.09	0	5.88	3844.83
BD_10	−0.26	NANTI	−0.08	−0.2	−0.83	0.44	2	12.01	1819.31
BD_8	0.25	NANTI	−0.48	−2.34	−0.84	0.98	4	12.48	1760.18
BD_7	0.02	NANTI	−0.4	−2.21	−1.07	0.89	4	12.48	1946.41
BD_12	0.003	NANTI	−0.31	−1.83	−0.8	0.68	12	13.05	6494.01

*Note:* The following table presents the results of analyzing the designed sequences to determine whether they exhibit antifungal activity. We tested the 12 designed sequences, and their hydrophobicity, hydrophilicity, amphipathicity, isoelectric point charge, and molecular weight were determined using the Antifp program [101]. The results showed that none of the sequences had a potential antifungal effect (NANTI).

To demonstrate that these sections were identified as such, the Antifp program was utilized, which, like other programs, searches within protein sequences of a particular length for similarities between key amino acids known to have a specific effect, in this case, antifungal activity. These programs are linked to databases of sequences in which this potential effect has already been defined and tested experimentally in the laboratory. The results obtained for this part of the analysis were NANTI (nonantifungal) negative in all cases. In other words, despite the addition of a modified sequence that was registered as an antifungal during the evolution of this protein, the sequence, together with the other segments, did not retain its potential activity in any of the cases (see Table 2). The same program provided us with interesting information about the sequences introduced, including their net charge, the respective indices of hydrophobicity, hydrophilicity, and amphiphilicity, as well as the molecular weight of each sequence and its isoelectric point.

In the process of designing proteins as potential treatments, specific key characteristics must be met. For example, the designed protein must not exhibit hemolytic activity; it may or may not (although it is preferable) have an inhibitory effect on biofilm formation. Additionally, it is also of interest to determine the stability of this protein and its half‐life in vitro experiments. To test engineered proteins in all these areas, various programs are available, such as ProtParam, which helps determine the half‐life of proteins. BIOFIN, which can identify proteins that may have inhibitory effects on bacterial biofilm formation, and HemoPI, which helps distinguish between constructs with hemolytic activity and those without (see Table 3).

**TABLE 3:** Properties of designed sequences.

ID	ProtParam			BIOFIN		HemoPI
	Reticulocyte	Levadure	*E. coli*	Score	Prediction	PROB score (hemolytic)
BD_1	1 h	30 min	> 10 h	−0.659775	Negative	0.18
BD_2	1 h	30 min	> 10 h	−0.411233	Negative	0.14
BD_3	1 h	30 min	> 10 h	−1.19434	Negative	0.11
BD_4	1 h	30 min	> 10 h	0.189585	Negative	0.4
BD_5	1 h	30 min	> 10 h	−0.521029	Negative	0.47
BD_6	1 h	30 min	> 10 h	−0.732722	Negative	0.47
BD_7	1 h	2 min	2 min	−0.235444	Negative	0.76
BD_8	1 h	2 min	2 min	−0.274915	Negative	0.78
BD_9	30 h	> 20 h	> 10 h	−0.442545	Negative	0.27
BD_10	30 h	> 20 h	> 10 h	−0.122223	Negative	0.87
BD_11	1 h	30 min	10 min	−0.223023	Negative	0
BD_12	1 h	2 min	2 min	−1.00677	Negative	0.93

*Note:* The following table shows the results of the analysis of the properties of the designed sequences. The half‐life properties of the proteins were analyzed using the program ProtParam, BIOFIN program, and HemoPI, which is a normalized PROB score, shows ranges of values between 0 and 1 (1: very likely to be hemolytic and 0: improbable to be hemolytic).

All designs were subjected to the three tests mentioned above; the results obtained were a half‐life of approximately 30 min in most experimental models, such as yeast, 1 h in models like reticulocytes, and more than 10 h in models like *E*. *coli*. BD_9 and BD_10 were obtained after 30 h in reticulocyte models, more than 20 h in yeast, and more than 10 h in *E*. *coli*, making them the best models at this stage (see Table 3). In the same sense, the design with the best result for HemoPI is BD_11, with a PROB score of 0, followed by BD_3, BD_2, and BD_1 models with 0.11, 0.14, and 0.18, respectively, which were considered very unlikely to be hemolytic. The results of the BIOFIN program indicate that none of the designs have potential inhibitory activity for biofilm formation (see Table 3).

Based on previous results, the best models were selected to study potential receptor–protein interactions using the HEX loria program. Up to this point, most analyses did not require a structural model. From this point on, it was necessary to generate the structural models of the designed proteins. The sequences were entered into an I‐TASSER structural prediction program [102], and then the structures were annotated and downloaded in PDB format for further analysis. The models that performed better in most tests were BD_7, BD_8, BD_9, BD_10, BD_11, and BD_12 (see Supporting Information S1–S6 and Table S1 y S2).

In addition to seeking a treatment that targets specific opportunistic bacteria, they also attempted to identify a sequence with antifungal properties to create a more comprehensive and targeted treatment. In the case of *Bd*, we worked as we did with bacteria. In *Bd*, they looked for membrane‐specific proteins, a membrane, and a wall with a net negative charge and a complementary sequence to the specific protein located in the membrane. This new short sequence was added to the designed sequences that passed the previous filtering (see Supporting Information S1–S6 and Table S1 y S2).

**FIGURE 2:**
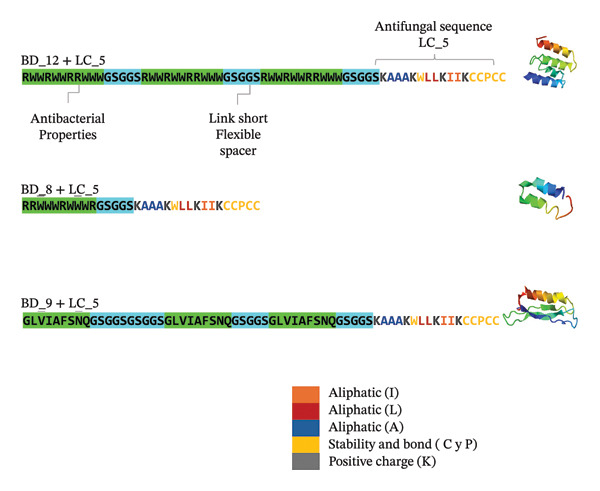
Construction of a sequence with several molecular targets. This is shown in the figure; in the green and cyan sections are the sequences of the proteins that passed the filters, and in the N‐terminal region, we can see the anti‐*Bd* sequence in the colors orange, red, blue, yellow, and gray, which have activity specified in the box in the figure. These new sequences are named as the first sequence name BD+ the sequence number + the antifungal sequence LC_5 = BD#LC5, which is equal to BD12LC5, BD8LC5, BD9LC5, and BD11LC5.

This new design features four main sections: negatively charged amino acids, positively charged amino acids, aromatic amino acids, and a cysteine tail that not only helps stabilize this protein section in itself but also confers this attribute when added to any of the aforementioned BD sequences. This addition was designated LC5, and the conjugates were named BD#LC5. The binding of each sequence was facilitated by a short, flexible spacer sequence, allowing for the natural folding of both parts into a complete protein (see Figure 2). The tests performed to select the LC5 fragment are described in the Supporting Information Figures S1 y S2.

After ensuring that the desired potential activity of each sequence segment was present, we proceeded to investigate whether the protein as a whole could bind to the walls or membranes, which were assumed to be negatively charged. For this part of the study, the PMIpred program was used, which performs the necessary calculations to predict the interactions between proteins or peptides and membranes, labeling them as BINDER (binding/interaction), NON‐BINDER (not binding/no interaction), and SENSOR (identifying sensor proteins, they do not bind but there is some interaction) [103] (see Supporting Information S1–S6 and Table S1 y S2).

**FIGURE 3:**
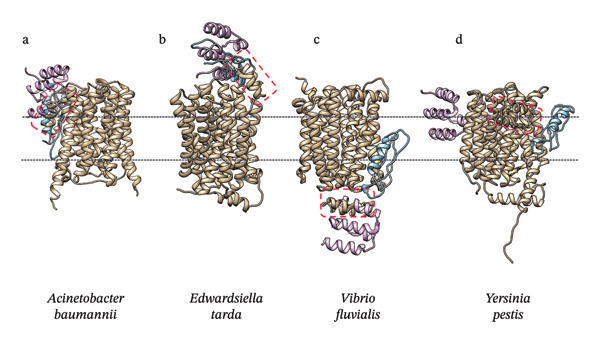
Final molecular docking and final model. The following figure displays the designed proteins BD8LC5, BD9LC5, and BD12LC5 that were selected for the final test because they obtained the lowest scores in HemoPI, the predicted antimicrobial prediction activity in CAMPr4, and an extensive sequence in PMIpred, as well as good half‐life parameters in ProtParam. The structures of each white protein present in each of the pathogenic bacteria are located in the sand. The purple arrow indicates the location of BD8LC5, the blue arrow indicates BD9LC5, and the violet arrow indicates the result for BD12LC5.

The final selection was made based on the best molecular docking result obtained, which implies the fulfillment of several characteristics, such as the designed protein being as close as possible to its target protein and being located in the extracellular plane of the plasma membrane. In addition, the designed protein must possess the aforementioned properties in at least three of the four target proteins or most of them. The models that best fit these assumptions were BD8LC5, BD9LC5, and BD12LC5 in *Acinetobacter baumannii*, *Edwardsiella tarda*, and *Yersinia pestis*, where an expected docking result was observed. However, in the case of *Vibrio fluvialis*, which is present in most of the data analyzed with 16sPIP, a negative result was found because the docking of the designed proteins is located in the lower part of the target proteins, an area characterized by being on the intracellular side of the plasma membrane. The expected mechanism of action with the BD#LC5 proteins designed in the several targets is shown in Figure 3.

It should be noted that all results derived from molecular docking, AMP prediction, and membrane‐binding analyses represent computational predictions and should not be interpreted as experimentally confirmed biological functions. Experimental validation is required to verify the actual activity and specificity of the molecule designed. It should be noted that the antifungal component was incorporated into the design following an initial prediction failure, representing an exploratory adjustment to the computational pipeline. Although the corresponding computational analyses were carried out to evaluate its potential function, this approach carries inherent limitations and should be interpreted with caution, as its inclusion was not part of the original experimental design. Experimental validation will be essential to assess the actual antifungal potential of the final molecule.

## 4. Discussion

It is important to acknowledge that 16S rRNA‐based identification has well‐recognized limitations for species‐level resolution, particularly in wildlife microbiomes, where closely related taxa may share highly similar sequences, increasing the risk of false positives. Whole‐genome sequencing or metagenomic approaches would be required for definitive species‐level experimental conformation and *in vitro* validation.

While the selection of conserved MFS transporters as molecular targets provides broad applicability, it also raises concerns regarding potential collateral effects on beneficial bacterial strains and the risk of exacerbating microbiota dysbiosis. These limitations must be considered when interpreting our results. Future refinement strategies should include the isolation and phenotypic characterization of the identified target bacteria, as well as site‐directed mutagenesis experiments to improve the selectivity of the designed molecule. Importantly, the computational approach employed here reduces the need to synthesize and experimentally test a large number of candidates, streamlining the path toward experimental validation by narrowing the selection to the most promising molecules.

This type of comprehensive methodology presents a wide range of opportunities, as it could improve the criteria for selecting pathogenic and opportunistic microorganisms to be targeted for treatment. Additionally, this study has some limitations, including the databases used and the pathogenic microorganism sequence prediction program 16sPIP, which is a small database comprising only 155 pathogens, most of which are human pathogens rather than animal pathogens. The tests that can be performed on the designed protein models have improved in recent years, and more tests can be performed. However, they are still limited. Nevertheless, a result was obtained that the currently available tools can ensure their interaction in negatively charged membranes of pathogenic microorganisms, in addition to the fact that some of them will have a fairly adequate half‐life. The sequence and model designed with the anti‐Bd potential activity also had good parameters to follow its effects. The importance of this type of design lies in the fact that the sequences can be optimized until the desired result is achieved at a low cost. Additionally, a significant number of new molecules with antimicrobial potential can be identified, which is particularly important given the increasing prevalence of antibiotic resistance. The following steps are protein synthesis and further in vitro testing on the key microorganisms mentioned in this article. In addition, with the technology now available, an efficient method of administration to wildlife could be ensured, thus supporting the conservation of these animals.

No endemic pathogens or amphibian‐specific pathogens that cause infections other than the detected Bd were sought. The search focused on pathogens in general, i.e., those that are usually recognized as opportunistic, in the sense that although they are mainly causes of infection in humans, they are often zoonotic and can have a wide range of hosts [104]. In addition, many of these bacteria have been identified as microorganisms capable of having positive or negative interactions in the microbiota [18, 42–44, 105–108] and some other bacteria may be capable of altering the efficiency of treatments [19, 21–24, 109, 110]. This has already been described in humans, although there are currently no records specifically for amphibians, nor is there sufficient literature identifying pathogens of any kind in this study model.

The bacteria identified here may be capable of causing dysbiosis among the microorganisms present in a healthy or sick animal, which may also affect the health of the host [111, 112]. The only condition taken into account for this occasion was that the bacteria were classified as pathogenic and were not present in healthy animals at the time of identification. Healthy animals were analyzed following the same protocol, and no such bacteria were found in them (results not shown here). There are records of pathogens that cause infection in humans and can also cause infection in animals, an event known as zoonosis, which, as mentioned, can be bilateral. Some of these microorganisms are classified as “opportunistic pathogens” [113–115]. Although these interactions have not yet been described for the pathogens reported in this manuscript, this is another area of research that should/could be addressed in the future.

Studies have shown that the more targets a protein design has, the more complicated it will be, both *in silico* and experimentally, to achieve the desired potential activity and required effectiveness. However, new design support tools such as Rosetta are extremely helpful [115–120]. For this reason, the number of targets was limited, using a total of four in this study. And it is for this reason that the 16sPIP tool was used, because it is capable of providing us with a “filtered” list of possible target bacteria. If the design were successful in experimental tests, its effectiveness against Bd would not be limited nor would it lose effectiveness; because although a multitarget protein is proposed, the basis of that protein and the design is a sequence section that is exclusively targeted against Bd, which has also been tested, including molecular docking, with favorable results [74]. Although this could be considered a limitation of our work, we believe it is a good starting point for designing specific proteins as treatments. In addition to the findings regarding pathogens other than Bd in infected animals, our work contributes a method that could work against any type of pathogen of biomedical interest. For now, however, we are testing it against a small list of bacteria.

To our knowledge, the bacterial taxa identified in this study have not been previously reported or characterized in amphibian skin microbiota, and their functional role in salamander health remains unknown. While none of the detected genera have been formally cataloged as pathogens or opportunistic organisms directly affecting amphibian skin, some have been recognized as potential pathogens in human clinical settings. This warrants careful consideration, as their presence in Bd‐infected salamanders may have implications for wildlife health that remain to be investigated through experimental studies.

## 5. Conclusions

This work represents the first approach to designing an anti‐*Bd* fungus treatment. This proposal takes into account specific molecular targets in different microorganisms: on the one hand, the *Bd* fungus to alleviate the skin infection caused by this fungus, and, on the other hand, opportunistic pathogenic microorganisms that could be harmful to the host; altogether to design a protein capable of destabilizing the structure of the microbial communities on the skin of infected salamanders, so that other chemical treatments, which have not been effective, or the design proposed here per se, achieve their objective: to alleviate the *Bd* fungus.

## Author Contributions

Jimena Ramírez‐Villarreal: writing–original draft, investigation, conceptualization, visualization, and methodology. Roberto Álvarez‐Martínez: conceptualization, funding acquisition, writing–review and editing, project administration, and supervision.

## Funding

This work was supported by the scholarship from the Consejo Nacional de Humanidades, Ciencias y Tecnologías (CONAHCyT) currently, Secretaría de Ciencia, Humanidades, Tecnología e Innovación (SECIHTI) (1003112).

## Ethics Statement

The present work has been approved by the Bioethics Committee of the University Autonomous of Queretaro, Faculty of Natural Sciences, with the ID number 083FCN2023.

## Conflicts of Interest

The authors declare no conflicts of interest.

## Supporting Information

Supporting Information:

File 1: Supporting_Information_S1.pdf.

Complement_Figures_information: This archive shows the complement figures with the table of pathogens identify, sequence alignment of molecular targets, three best models predicted and the results of BINDER assays, the 12 models predicted, and the structure of final treatment sequence (with antifungal section).

File 2: Structural_Comparison_AlphaFold_vs_Original_Main_Manuscript_Models_5903624_S2.pdf.

File 1 (PDF): It shows a comparison of the structural models that were originally obtained in the manuscript using the I‐TASSER program and trRosetta against the structural results obtained using AlphaFold.

File 2: Supporting_Information_Figures_S3_S4.docx.

Figure 3S: Results of molecular docking between the LC5 protein model and the Na^+^/H^+^ transporter from *Bd*.

Figure 4S: Distance measurements between the designed LC5 protein and the *Bd* transporter, performed in Chimera, showing interaction ranges in angstroms.

File 3: Possible_Mechanism_of_Action_Proteins_Designed_BD#LC5_5903624_S5.pdf.

File 3 (PDF): It shows the expected mechanism of action proteins designed in both specific membrane proteins present in the fungus Bd and the bacteria targets. Additionally, the explanation of the mechanism and some references about it.

File 4: CASTpFold_Results_5903624_S6.pdf.

File 4 (PDF): It shows all the results obtained from CASTpFold and the transporter surface analysis. Each transporter target has this analysis. The figures show each transporter and the pockets present on its surface. These pockets contain several amino acids that may match other amino acids present in the BD#LC5 proteins. This list of amino acids is in the same document as a large table with the name and amino acid number of each amino acid.

File: Table S1_extracted_species.xlsx.

Table S1. Complete list of bacterial species detected across amphibian samples. For each bacterium, the total number of sequence matches and relative abundance percentage are reported. Presence (1) or absence (0) of each bacterial species in amphibian hosts is indicated across columns named by host species.

File: Table S2 Energy values for sequence.xlsx.

Table S2. Complete list of energy values by sequence and amino acids obtained as result by the program PMIpred. Helps predict which amino acids would or would not have contact with the membrane. The designed protein models were individually analyzed with this tool.

## Supporting information


**Supporting Information** Additional supporting information can be found online in the Supporting Information section.

## Data Availability

The skin microbiota of the axolotl *Ambystoma altamirani* (https://www.ncbi.nlm.nih.gov/sra/?term=PRJNA819099) ID Bioproject: PRJNA819099. Salamander skin microbiome and association with mucosome (https://www.ncbi.nlm.nih.gov/sra/?term=PRJNA659464) ID BIOPROJECT PRJNA659464. Processed Data (https://github.com/VillarrealJ24/Art_II).
