# Supplementary material for: Identification of Opportunistic Pathogens on the Skin of Salamanders for Use as Molecular Targets of a De Novo Design of Multitarget Anti‐Bd Proteins
Source: Int J Microbiol. 2026 Apr 20;2026:5903624. doi: 10.1155/ijm/5903624 (PMC13093180; doi:10.1155/ijm/5903624)
Supplement: Supplementary file 1 — Supporting Information Additional supporting information can be found online in the Supporting Information section. [file IJM-2026-5903624-s001.zip › CASTpFold_Results.pdf]

CASTpFold

Computed Atlas of Surface Topography of the universe of protein Folds

Home Compute News About

input PDB, AF2, or job id

The CASTpFold server is free and open to all users, and there is no login requirement.
Your citation is really important to us. Please cite this paper if you publish or present results using CASTpFold analysis:
CASTpFold: computed atlas of surface topography of the universe of protein Folds.
***Nucleic Acids Res*. 2024. PMID: 38783102. DOI: 10.1093/nar/gkae415.**
Bowei Ye, Wei Tian, Boshen Wang, and Jie Liang.

j_68d487d403791

Download CASTpFold Data

Pocket Info

	Pocket ID	Area (SA) ($Å^2$)	Volume (SA) ($Å^3$)
-	1	5808.748	59171.347

Show negative volume: Negative volume color: Representation style: Cartoon

Atom Info

Chain	Seq ID	AA	ATOM
A	15	TRP	O

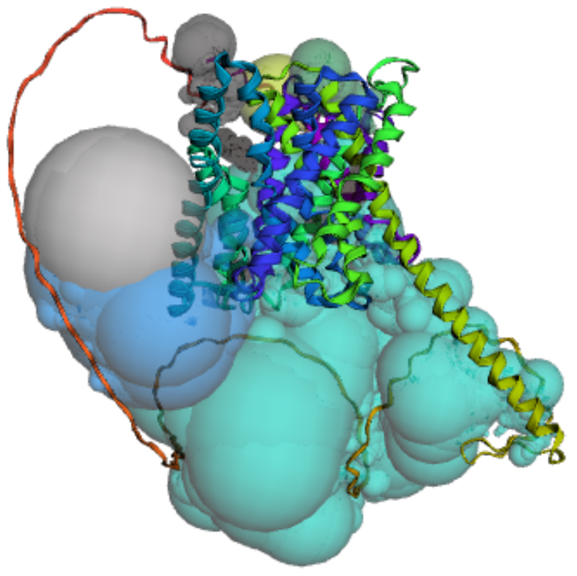

Pocket ID	Area (SA) (Å^2)	Volume (SA) (Å^3)

Chain	Seq ID	AA	ATOM
A	15	TRP	CE3
A	15	TRP	CZ3
A	15	TRP	CH2
A	18	LEU	C
A	18	LEU	O
A	18	LEU	CB
A	18	LEU	CD1
A	18	LEU	CD2
A	19	ILE	CA

< 1 2 3 4 5 ••• 84 >

	Pocket ID	Area (SA) (Å^2)	Volume (SA) (Å^3)
-	2	678.692	4913.953

Show negative volume : Negative volume color : Representation style : Cartoon

Atom Info

Take Screenshot Spectrum

Pocket ID	Area (SA) ($Å^2$)	Volume (SA) ($Å^3$)

Chain	Seq ID	AA	ATOM
A	111	ARG	CG
A	111	ARG	NE
A	111	ARG	NH2
A	112	SER	CB
A	114	ILE	CB
A	114	ILE	CG1
A	114	ILE	CG2
A	114	ILE	CD1
A	115	TRP	N
A	115	TRP	O

< 1 2 3 4 5 ••• 13 >

	Pocket ID	Area (SA) ($Å^2$)	Volume (SA) ($Å^3$)
-	3	25.012	415.431

Show negative volume : Negative volume color : Representation style : Cartoon

Pocket ID	Area (SA) ($Å^2$)	Volume (SA) ($Å^3$)

Atom Info

Chain	Seq ID	AA	ATOM
A	227	ILE	O
A	227	ILE	CG1
A	227	ILE	CG2
A	227	ILE	CD1
A	231	VAL	CG1
A	231	VAL	CG2
A	232	LEU	N
A	232	LEU	CA
A	232	LEU	CB
A	232	LEU	CD1

< 1 2 >

	Pocket ID	Area (SA) ($Å^2$)	Volume (SA) ($Å^3$)
-	4	440.110	322.878

Pocket ID	Area (SA) (Å^2)	Volume (SA) (Å^3)

Show negative volume : Negative volume color : Representation style : Cartoon

Atom Info

Chain	Seq ID	AA	ATOM
A	203	PHE	CD1
A	203	PHE	CE1
A	206	VAL	CG1
A	207	TYR	CA
A	207	TYR	CD1
A	207	TYR	CD2
A	207	TYR	CE1
A	207	TYR	CE2
A	207	TYR	CZ
A	207	TYR	OH

< 1 2 3 4 5 ••• 11 >

	Pocket ID	Area (SA) (Å²)	Volume (SA) (Å³)
-	5	106.225	288.940

Show negative volume : Negative volume color : Representation style : Cartoon

Atom Info

Chain	Seq ID	AA	ATOM
A	4	THR	O
A	6	GLY	N
A	6	GLY	CA
A	6	GLY	C
A	6	GLY	O
A	7	HIS	C
A	7	HIS	O
A	8	ILE	CA
A	8	ILE	CG1
A	8	ILE	CG2

< 1 2 3 4 >

	Pocket ID	Area (SA) ($Å^2$)	Volume (SA) ($Å^3$)
-	6	274.326	287.819

Show negative volume : Negative volume color : Representation style : Cartoon

Atom Info

Chain	Seq ID	AA	ATOM
A	66	ILE	CA
A	66	ILE	O
A	69	GLY	CA
A	70	PRO	CB
A	70	PRO	CG
A	70	PRO	CD
A	71	ALA	N
A	71	ALA	CB
A	77	ASN	OD1
A	78	PRO	CB

< 1 2 3 4 5 6 7 8 >

	Pocket ID	Area (SA) ($Å^2$)	Volume (SA) ($Å^3$)
-	7	99.703	34.775

Show negative volume : Negative volume color : Representation style : Cartoon

Atom Info

Chain	Seq ID	AA	ATOM
A	95	ARG	NH1
A	95	ARG	NH2
A	199	ALA	CB
A	203	PHE	CE2
A	229	GLN	CG
A	229	GLN	CD
A	229	GLN	OE1
A	278	SER	CA
A	278	SER	O
A	278	SER	CB

	Pocket ID	Area (SA) ($Å^2$)	Volume (SA) ($Å^3$)
-	8	75.789	< 1 2 5[illegible] 3 4 >

Show negative volume : Negative volume color : Representation style : Cartoon

Atom Info

Chain	Seq ID	AA	ATOM
A	132	MET	CE
A	351	ALA	CA
A	351	ALA	O
A	351	ALA	CB
A	354	LEU	C
A	354	LEU	O
A	354	LEU	CB
A	354	LEU	CD1
A	355	VAL	N
A	355	VAL	CA

	Pocket ID	Area (SA) ($Å^2$)	Volume (SA) ($Å^3$)
-	9	28.781	4

< 1 2 3 4 >

Show negative volume: Negative volume color: Representation style: Cartoon

Atom Info

Chain	Seq ID	AA	ATOM
A	109	LEU	O
A	109	LEU	CB
A	110	PRO	C
A	110	PRO	O
A	111	ARG	CA
A	112	SER	N
A	112	SER	CA
A	113	PHE	N
A	113	PHE	CB
A	114	ILE	N

Pocket ID	Area (SA) (Å^2)	Volume (SA) (Å^3)
10	35.171	2

< 1 2 3 >

Show negative volume : Negative volume color : Representation style : Cartoon

Atom Info

Chain	Seq ID	AA	ATOM
A	161	THR	CB
A	161	THR	OG1
A	161	THR	CG2
A	166	ALA	CA
A	166	ALA	CB
A	169	ILE	CB
A	169	ILE	CG2
A	169	ILE	CD1
A	170	VAL	CG1
A	187	LEU	O

Pocket ID	Area (SA) ($Å^2$)	Volume (SA) ($Å^3$)

Sequence info ⓘ

Chain A

M D T T I G H I D S S H G T W N E L I P E D P G L R T A H L T Y L L L G G F V G G F G L I S L I V K D R L Y M S E A M V A T I F G I I I G P A G C N I F N P E A L F
P G Q V N H V I L E F S R L V I A I Q C L V A G I S L P R S F I W K E R Q S I T M L L G P V M L Y M W L M S A L A I W V V C G V P W Q L A L I I A G C L T P T D P V
L A N S I V K G K F A E K H I P Y H V R L L L S A E S G S N D G F G A P L V F F P V Y I W R M S N T G E A I G W W L V N V V I Y Q V V L S I V L G V I I G T L S R K
A L K C A E S N G W I D K E S I L G F F I A I A L V T T G A L S L L G V D D V L G C F V V G A I L S W D D W F N R Q I E E T H I Q E V L D A L I N M S F F I V F G T
L I P W H E Y T A V E G V L S I W R M I L V A L W L V L L R R L P I V V L L S R W M P A L K N Q K E V F F C G W F G P I G V G A L F Y C M I A V I Y L Q I D Y K P L
F P I V A F M V L S S I V L H G G S V S L F E I G I T R H S T W Q N N R L A A Q I A S A M Q E Q Q G N Q G N Q V V I G G L V I P S N V T P A T V E S R S V I E I P Q
S K E D Q A N P G R D A S Q I D Q D C V S E R K G E G V P T G R E S G A T V I Q S V S A M D F E N T N P R S S L D I R R S I N A D R S M Q D T I P L E E N T N S G S
R Q P K I Y T F Q

CASTpFold

Computed Atlas of Surface Topography of the universe of protein Folds

Home Compute News About

input PDB, AF2, or job id

The CASTpFold server is free and open to all users, and there is no login requirement.
Your citation is really important to us. Please cite this paper if you publish or present results using CASTpFold analysis:
CASTpFold: computed atlas of surface topography of the universe of protein Folds.
***Nucleic Acids Res*. 2024. PMID: 38783102. DOI: 10.1093/nar/gkae415.**
Bowei Ye, Wei Tian, Boshen Wang, and Jie Liang.

j_68d5ad6e3defd

Download CASTpFold Data

Pocket Info ?

	Pocket ID	Area (SA) (Å^2)	Volume (SA) (Å^3)
-	1	2838.097	3047.333

Show negative volume: Negative volume color: Representation style: Cartoon

Atom Info

Chain	Seq ID	AA	ATOM
A	8	ARG	NH1
A	28	ALA	CA
A	28	ALA	O
A	28	ALA	CB

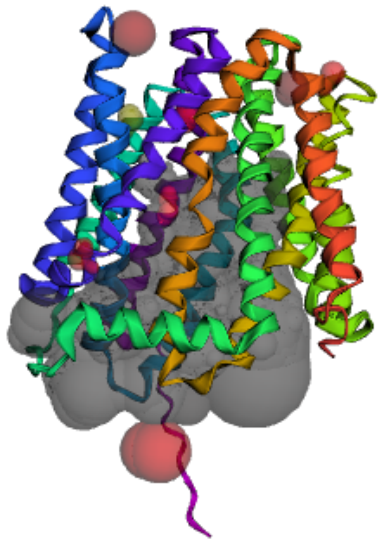

Take Screenshot Spectrum

Pocket ID	Area (SA) (Å^2)	Volume (SA) (Å^3)

Chain	Seq ID	AA	ATOM
A	31	SER	CA
A	31	SER	O
A	31	SER	CB
A	31	SER	OG
A	32	LYS	O
A	32	LYS	CB

< 1 2 3 4 5 ••• 46 >

	Pocket ID	Area (SA) (Å^2)	Volume (SA) (Å^3)
-	2	97.168	58.082

Show negative volume: Negative volume color: Representation style: Cartoon

Atom Info

Chain	Seq ID	AA	ATOM
A	7	TYR	O
A	8	ARG	CA
A	8	ARG	CB
A	8	ARG	CG
A	8	ARG	CD

+

Pocket ID	Area (SA) (Å^2)	Volume (SA) (Å^3)

Chain	Seq ID	AA	ATOM
A	8	ARG	NE
A	8	ARG	CZ
A	8	ARG	NH1
A	8	ARG	NH2
A	9	THR	N

< 1 2 3 >

	Pocket ID	Area (SA) (Å^2)	Volume (SA) (Å^3)
-	3	15.603	8.422

Show negative volume: Negative volume color: Representation style: Cartoon

Atom Info

Chain	Seq ID	AA	ATOM
A	4	LEU	CD2
A	5	THR	O
A	7	TYR	N
A	7	TYR	CA
A	7	TYR	C
A	7	TYR	O

+

Pocket ID	Area (SA) (Å^2)	Volume (SA) (Å^3)

Chain	Seq ID	AA	ATOM
A	7	TYR	CB
A	140	GLU	OE1
A	140	GLU	OE2

< 1 >

-	4	7.999	5.397

Show negative volume: Negative volume color: Representation style: Cartoon

Atom Info

Chain	Seq ID	AA	ATOM
A	51	MET	CE
A	108	LEU	CB
A	108	LEU	CD1
A	109	TYR	CD1
A	109	TYR	CE1
A	109	TYR	CE2
A	109	TYR	CZ
A	109	TYR	OH

Pocket ID	Area (SA) (Å^2)	Volume (SA) (Å^3)
5	17.600	4.726

< 1 >

Show negative volume: Negative volume color: Representation style: Cartoon

Atom Info

Chain	Seq ID	AA	ATOM
A	370	LEU	O
A	370	LEU	CB
A	370	LEU	CD1
A	370	LEU	CD2
A	374	TYR	CB
A	374	TYR	CD2
A	375	GLY	O
A	378	GLU	C
A	378	GLU	CB
A	379	LEU	N

< 1 2 >

Pocket ID	Area (SA) (Å^2)	Volume (SA) (Å^3)
6	22.046	3.902

Show negative volume: Negative volume color: Representation style: Cartoon

Pocket ID	Area (SA) (Å^2)	Volume (SA) (Å^3)

Atom Info

Chain	Seq ID	AA	ATOM
A	34	TYR	CE2
A	34	TYR	OH
A	38	LEU	CD1
A	93	THR	CG2
A	94	ILE	CD1
A	97	SER	CB
A	97	SER	OG
A	111	LEU	O
A	111	LEU	CD2
A	114	CYS	C

< 1 2 >

	Pocket ID	Area (SA) (Å^2)	Volume (SA) (Å^3)
-	7	15.167	2.813

Show negative volume: Negative volume color: Representation style: Cartoon

Atom Info

Chain	Seq ID	AA	ATOM

+

Pocket ID	Area (SA) (Å^2)	Volume (SA) (Å^3)

Chain	Seq ID	AA	ATOM
A	38	LEU	CG
A	38	LEU	CD2
A	39	PRO	CA
A	39	PRO	CG
A	39	PRO	CD
A	42	VAL	CB
A	42	VAL	CG2
A	52	MET	CA
A	52	MET	O
A	55	MET	CB

< 1 2 >

	Pocket ID	Area (SA) (Å^2)	Volume (SA) (Å^3)
-	8	13.255	1.703

Show negative volume: Negative volume color: Representation style: Cartoon

Atom Info

Chain	Seq ID	AA	ATOM
A	68	LEU	CB

Pocket ID	Area (SA) (Å^2)	Volume (SA) (Å^3)

Chain	Seq ID	AA	ATOM
A	68	LEU	CD1
A	69	PHE	CE1
A	69	PHE	CZ
A	207	LEU	CD2
A	208	TRP	CZ2
A	208	TRP	CH2
A	211	LEU	CD1

< 1 >

	Pocket ID	Area (SA) (Å^2)	Volume (SA) (Å^3)
-	9	15.451	1.609

Show negative volume: Negative volume color: Representation style: Cartoon

Atom Info

Chain	Seq ID	AA	ATOM
A	60	PHE	O
A	60	PHE	CB
A	63	ASN	C
A	63	ASN	O

+

Pocket ID	Area (SA) (Å^2)	Volume (SA) (Å^3)

Chain	Seq ID	AA	ATOM
A	63	ASN	CB
A	64	LEU	N
A	64	LEU	CA
A	64	LEU	CB
A	64	LEU	CD1
A	354	SER	CA

< 1 2 >

Pocket ID	Area (SA) (Å^2)	Volume (SA) (Å^3)
- 10	9.865	1.520

Show negative volume: Negative volume color: Representation style: Cartoon

Atom Info

Chain	Seq ID	AA	ATOM
A	248	PHE	CE2
A	248	PHE	CZ
A	305	PRO	C
A	305	PRO	O
A	305	PRO	CB

+

Pocket ID	Area (SA) ($Å^2$)	Volume (SA) ($Å^3$)

Chain	Seq ID	AA	ATOM
A	306	VAL	N
A	306	VAL	CA
A	306	VAL	CG2
A	309	PRO	CB
A	377	ALA	CA

< 1 2 >

< 1 2 3 4 5 >

Sequence info

Chain A

M N T L T D Y R T S I W K S S S F V I L F S S A F F V A L G S K I Y D L A L P L L V Y D L T Q S S E M M G W M R A V E F L P N L L L A L F I G V W V D R F D K K Q W S Q V
M L L G Q I V T I L I S Y S A V N W M T E P L Y V L F P C A F L M M A F N Y G Y H N A R M A M L K L A L P Q E Q Q N T A T A R M S S L Y S L M E T V G P V L S G A L L L L
S Q L H Q I F L G L A L L W L L A Y W Q L N R L T L E T P Q V S D H P P L W Q A L R E G W Q V L Y N E K N M W L I T L A V M V I N T T G S I F W I Q S I Y F A K A E L S L
S A I E V G Y M V A A S G I G G V L S A F V A D K V R R Q I G L G K L L I L S I A L E S V G F V L P V L S P T P M M L I V A F F W V S A V G L F S N I C I W S Y R Q E A F
S Q Q H L G R I A G L T G S L F K L L M P F G L A A S G Y L V T R Y G S A E L F S V C F A V Q F V V A I F L L F S R V R A I P

+

CASTpFold

Computed Atlas of Surface Topography of the universe of protein Folds

Home Compute News About

input PDB, AF2, or job id

The CASTpFold server is free and open to all users, and there is no login requirement.
Your citation is really important to us. Please cite this paper if you publish or present results using CASTpFold analysis:
CASTpFold: computed atlas of surface topography of the universe of protein Folds.
Nucleic Acids Res. 2024. PMID: 38783102. DOI: 10.1093/nar/gkae415.
Bowei Ye, Wei Tian, Boshen Wang, and Jie Liang.

j_68d5b2234cd5c

Download CASTpFold Data

Pocket Info ⓘ

	Pocket ID	Area (SA) (Å^2)	Volume (SA) (Å^3)
-	1	1139.758	1491.036

Show negative volume: Negative volume color: Representation style: Cartoon

Atom Info

Chain	Seq ID	AA	ATOM
A	19	TRP	CZ2
A	19	TRP	CH2
A	26	PHE	CD1

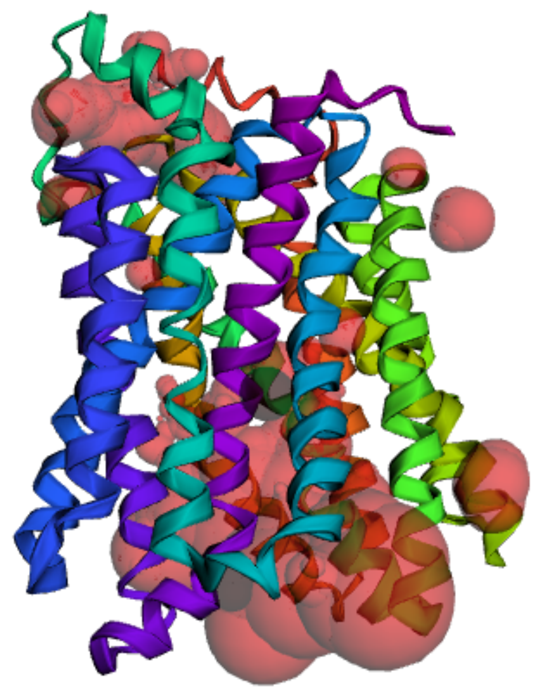

Take Screenshot Spectrum

Pocket ID	Area (SA) (Å^2)	Volume (SA) (Å^3)

Chain	Seq ID	AA	ATOM
A	26	PHE	CE1
A	26	PHE	CZ
A	27	PHE	CD1
A	27	PHE	CE1
A	27	PHE	CZ
A	30	VAL	O
A	30	VAL	CG1

< 1 2 3 4 5 ••• 21 >

	Pocket ID	Area (SA) (Å^2)	Volume (SA) (Å^3)
-	2	392.435	287.683

Show negative volume: Negative volume color: Representation style: Cartoon

Atom Info

Chain	Seq ID	AA	ATOM
A	70	GLU	O
A	70	GLU	CB
A	70	GLU	OE2
A	71	LYS	CA

Pocket ID	Area (SA) (Å^2)	Volume (SA) (Å^3)

Chain	Seq ID	AA	ATOM
A	71	LYS	O
A	74	ARG	NH1
A	74	ARG	NH2
A	189	VAL	CG1
A	190	TRP	CA
A	190	TRP	O

< 1 2 3 4 5 6 7 8 >

	Pocket ID	Area (SA) (Å^2)	Volume (SA) (Å^3)
-	3	29.904	18.030

Show negative volume: Negative volume color: Representation style: Cartoon

Atom Info

Chain	Seq ID	AA	ATOM
A	252	ILE	CG2
A	253	ILE	CA
A	253	ILE	CG1
A	256	SER	CB
A	256	SER	OG

+

Pocket ID	Area (SA) ($Å^2$)	Volume (SA) ($Å^3$)

Chain	Seq ID	AA	ATOM
A	301	SER	O
A	302	LEU	CA
A	302	LEU	O
A	302	LEU	CD1
A	305	GLY	CA

< 1 2 >

-	4	20.729	8.878

Show negative volume: Negative volume color: Representation style: Cartoon

Atom Info

Chain	Seq ID	AA	ATOM
A	267	PHE	CA
A	267	PHE	O
A	267	PHE	CD2
A	267	PHE	CE2
A	270	THR	CB
A	270	THR	CG2

+

Pocket ID	Area (SA) (Å^2)	Volume (SA) (Å^3)

Chain	Seq ID	AA	ATOM
A	271	LEU	N
A	271	LEU	CA
A	271	LEU	CB
A	271	LEU	CD1

< 1 2 >

	Pocket ID	Area (SA) (Å^2)	Volume (SA) (Å^3)
-	5	24.687	6.131

Show negative volume: Negative volume color: Representation style: Cartoon

Atom Info

Chain	Seq ID	AA	ATOM
A	67	ARG	CA
A	67	ARG	CG
A	67	ARG	NE
A	70	GLU	CB
A	70	GLU	CG
A	200	GLY	CA
A	201	MET	O

+

Pocket ID	Area (SA) (Å^2)	Volume (SA) (Å^3)

Chain	Seq ID	AA	ATOM
A	203	LEU	N
A	203	LEU	CA
A	203	LEU	CB

< 1 2 >

	Pocket ID	Area (SA) (Å^2)	Volume (SA) (Å^3)
-	6	28.932	5.649

Show negative volume: Negative volume color: Representation style: Cartoon

Atom Info

Chain	Seq ID	AA	ATOM
A	189	VAL	CA
A	189	VAL	O
A	189	VAL	CG1
A	189	VAL	CG2
A	192	ALA	C
A	192	ALA	CB
A	193	ALA	N
A	193	ALA	CA

+

	Pocket ID	Area (SA) (Å^2)	Volume (SA) (Å^3)

Chain	Seq ID	AA	ATOM
A	193	ALA	CB
A	196	HIS	CB

< 1 2 >

	Pocket ID	Area (SA) (Å^2)	Volume (SA) (Å^3)
-	7	6.967	1.588

Show negative volume: Negative volume color: Representation style: Cartoon

Atom Info

Chain	Seq ID	AA	ATOM
A	21	LEU	CD2
A	24	PHE	CE1
A	24	PHE	CZ
A	149	LEU	CD2
A	172	ILE	CB
A	172	ILE	CG2
A	172	ILE	CD1

< 1 >

	Pocket ID	Area (SA) (Å^2)	Volume (SA) (Å^3)
-	8	7.393	1.536

Pocket ID	Area (SA) (Å^2)	Volume (SA) (Å^3)

Show negative volume: Negative volume color: Representation style: Cartoon

Atom Info

Chain	Seq ID	AA	ATOM
A	32	LEU	CD2
A	35	ASP	CG
A	35	ASP	OD1
A	35	ASP	OD2
A	158	ALA	O
A	158	ALA	CB
A	162	GLY	CA
A	162	GLY	C
A	163	TRP	N

< 1 >

	Pocket ID	Area (SA) (Å^2)	Volume (SA) (Å^3)
-	9	7.308	1.480

Show negative volume: Negative volume color: Representation style: Cartoon

Atom Info

+

Pocket ID	Area (SA) (Å^2)	Volume (SA) (Å^3)

Chain	Seq ID	AA	ATOM
A	2	PHE	CE1
A	2	PHE	CZ
A	130	ARG	O
A	131	SER	CA
A	134	LEU	CB
A	270	THR	CG2
A	273	GLU	CG

< 1 >

	Pocket ID	Area (SA) (Å^2)	Volume (SA) (Å^3)
-	10	8.981	1.237

Show negative volume: Negative volume color: Representation style: Cartoon

Atom Info

Chain	Seq ID	AA	ATOM
A	62	ALA	CA
A	62	ALA	O
A	62	ALA	CB
A	66	GLY	N

+

Pocket ID	Area (SA) (Å^2)	Volume (SA) (Å^3)

Chain	Seq ID	AA	ATOM
A	66	GLY	CA
A	113	GLY	O
A	114	GLY	CA
A	114	GLY	O
A	117	GLY	CA
A	118	VAL	CG2

< 1 2 >

< 1 2 3 4 5 >

Sequence info ?

Chain A

M F A W S P Q Q R H V A I A S F L S W T L D A F D F F I L V F L L S D I A S A F H V D V E Q V T L A I L L T L A V R P I G A L I F G R A A E K Y G R R P I L M L N I I C F
S V F E L L S A A A P S L M I F L L L R V L Y G V A M G G I W G V A S S L T M E T I P D R S R G L M S G I F Q A G Y P F G Y L L A A V I Y G L F A Q W L G W R G M F V I G
A I P I V L L P Y I Y F Y V P E S P V W L A A K A H R Q S G M P L W P V L R H N W K L C I Y L V V L M A A F N F F S H G T Q D L Y P T F L K V Q H G F S A N V I S I I A V
S Y N I A S I A G G I F F G T L S E R I G R K R A I M L A A L L A L P V I P L W A F S G G S L M L G I G A F L M Q F M V Q G A W G V I P T Y L N E L V P A S A R A V L P G
F V Y Q L G N L L A S V N A T L Q A S I A A H H G Q N Y A L A M A I T A G S V A V I I A I L I S F G R D T R G V S M A E K S S E

+

CASTpFold

Computed Atlas of Surface Topography of the universe of protein Folds

Home Compute News About

input PDB, AF2, or job id

The CASTpFold server is free and open to all users, and there is no login requirement.
Your citation is really important to us. Please cite this paper if you publish or present results using CASTpFold analysis:
CASTpFold: computed atlas of surface topography of the universe of protein Folds.
***Nucleic Acids Res*. 2024. PMID: 38783102. DOI: 10.1093/nar/gkae415.**
Bowei Ye, Wei Tian, Boshen Wang, and Jie Liang.

j_68d5b4212fd0e

Download CASTpFold Data

Pocket Info

	Pocket ID	Area (SA) (Å^2)	Volume (SA) (Å^3)
-	1	1196.298	1145.665

Show negative volume: Negative volume color: Representation style: Cartoon

Atom Info

Chain	Seq ID	AA	ATOM
A	25	GLU	OE1
A	25	GLU	OE2
A	26	PHE	CE2
A	26	PHE	CZ

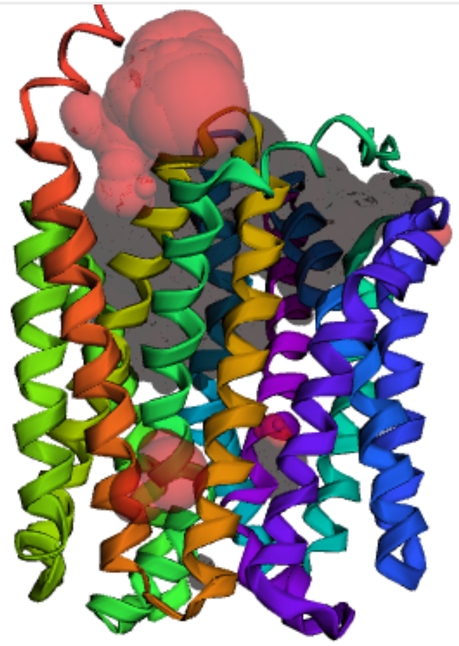

Take Screenshot Spectrum

Pocket ID	Area (SA) (Å^2)	Volume (SA) (Å^3)

Chain	Seq ID	AA	ATOM
A	57	VAL	O
A	57	VAL	CG1
A	58	MET	CA
A	58	MET	O
A	61	ALA	C
A	61	ALA	CB

< 1 2 3 4 5 ••• 22 >

	Pocket ID	Area (SA) (Å^2)	Volume (SA) (Å^3)
-	2	307.923	343.106

Show negative volume: Negative volume color: Representation style: Cartoon

Atom Info

Chain	Seq ID	AA	ATOM
A	204	ARG	CB
A	204	ARG	CG
A	204	ARG	CD
A	204	ARG	CZ
A	204	ARG	NH1

+

Pocket ID	Area (SA) (Å^2)	Volume (SA) (Å^3)

Chain	Seq ID	AA	ATOM
A	204	ARG	NH2
A	206	PRO	N
A	206	PRO	CA
A	206	PRO	O
A	206	PRO	CB

< 1 2 3 4 5 6 7 >

	Pocket ID	Area (SA) (Å^2)	Volume (SA) (Å^3)
-	3	33.437	22.872

Show negative volume: Negative volume color: Representation style: Cartoon

Atom Info

Chain	Seq ID	AA	ATOM
A	4	GLN	CA
A	4	GLN	CG
A	4	GLN	NE2
A	7	THR	CB
A	7	THR	OG1
A	7	THR	CG2

+

Pocket ID	Area (SA) (Å^2)	Volume (SA) (Å^3)

Chain	Seq ID	AA	ATOM
A	125	ILE	O
A	126	VAL	CA
A	126	VAL	CG1
A	127	PRO	CG

< 1 2 >

	Pocket ID	Area (SA) (Å^2)	Volume (SA) (Å^3)
-	4	45.559	18.637

Show negative volume: Negative volume color: Representation style: Cartoon

Atom Info

Chain	Seq ID	AA	ATOM
A	19	PHE	CE1
A	19	PHE	CZ
A	23	THR	CG2
A	149	GLY	CA
A	149	GLY	O
A	152	LEU	CB
A	152	LEU	CG

Pocket ID	Area (SA) ($Å^2$)	Volume (SA) ($Å^3$)

Chain	Seq ID	AA	ATOM
A	152	LEU	CD2
A	153	ALA	N
A	153	ALA	CA

< 1 2 >

	Pocket ID	Area (SA) ($Å^2$)	Volume (SA) ($Å^3$)
-	5	24.241	11.539

Show negative volume: Negative volume color: Representation style: Cartoon

Atom Info

Chain	Seq ID	AA	ATOM
A	348	ALA	CA
A	348	ALA	O
A	348	ALA	CB
A	351	LEU	CD2
A	352	VAL	CG1
A	357	TRP	CZ3
A	365	ALA	CA
A	365	ALA	O

+

Pocket ID	Area (SA) (Å^2)	Volume (SA) (Å^3)

Chain	Seq ID	AA	ATOM
A	365	ALA	CB
A	368	LEU	CD2

< 1 2 >

	Pocket ID	Area (SA) (Å^2)	Volume (SA) (Å^3)
-	6	16.512	4.682

Show negative volume: Negative volume color: Representation style: Cartoon

Atom Info

Chain	Seq ID	AA	ATOM
A	1	MET	CE
A	6	ASN	CA
A	6	ASN	O
A	6	ASN	CB
A	6	ASN	CG
A	6	ASN	OD1
A	9	PHE	CD1
A	182	LYS	O
A	183	ALA	CA

+

Pocket ID	Area (SA) ($Å^2$)	Volume (SA) ($Å^3$)

Chain	Seq ID	AA	ATOM
A	183	ALA	O

< 1 >

	Pocket ID	Area (SA) ($Å^2$)	Volume (SA) ($Å^3$)
-	7	10.356	2.725

Show negative volume: Negative volume color: Representation style: Cartoon

Atom Info

Chain	Seq ID	AA	ATOM
A	12	LEU	CD1
A	15	ALA	CB
A	140	MET	CE
A	141	GLY	N
A	141	GLY	CA
A	144	ILE	CD1

< 1 >

	Pocket ID	Area (SA) ($Å^2$)	Volume (SA) ($Å^3$)
-	8	6.405	1.049

Show negative volume: Negative volume color: Representation style: Cartoon

+

Pocket ID	Area (SA) (Å^2)	Volume (SA) (Å^3)

Atom Info

Chain	Seq ID	AA	ATOM
A	72	ALA	CB
A	74	ARG	CB
A	74	ARG	CD
A	75	ASN	OD1
A	75	ASN	ND2
A	186	GLN	CA
A	186	GLN	CB
A	186	GLN	CG
A	187	GLY	N

< 1 >

	Pocket ID	Area (SA) (Å^2)	Volume (SA) (Å^3)
-	9	6.667	0.842

Show negative volume: Negative volume color: Representation style: Cartoon

Atom Info

Chain	Seq ID	AA	ATOM
A	155	TRP	CD1

+

Pocket ID	Area (SA) (Å^2)	Volume (SA) (Å^3)

Chain	Seq ID	AA	ATOM
A	158	GLN	OE1
A	159	ASN	ND2
A	240	PRO	C
A	240	PRO	CB
A	241	THR	N
A	241	THR	CA
A	241	THR	OG1
A	244	THR	OG1

< 1 >

	Pocket ID	Area (SA) (Å^2)	Volume (SA) (Å^3)
-	10	8.164	0.833

Show negative volume: Negative volume color: Representation style: Cartoon

Atom Info

Chain	Seq ID	AA	ATOM
A	25	GLU	O
A	25	GLU	CG
A	29	MET	CE

+

Pocket ID	Area (SA) ($Å^2$)	Volume (SA) ($Å^3$)

Chain	Seq ID	AA	ATOM
A	50	ILE	CA
A	50	ILE	O
A	50	ILE	CG1
A	50	ILE	CG2
A	53	TYR	CB
A	53	TYR	CD1
A	104	ARG	NH2

< 1 2 >

< 1 2 3 4 5 >

Sequence info ?

Chain A

M N T Q S N T A F S L L A L A I G A F A I G T T E F S P M G L L P N I A N D L G I S I P T A G M L I T G Y A L G V M L G A P F M T L W F G G F A R R N A L I F L M A I F T
V G N L I A A F S P N Y M S L L G A R L I T S L N H G A F F G I G S V V A A S I V P A H K Q A S A V A T M F M G L T I A N I G G V P L A T W V G Q N I G W R M S F L A I S
V L G I I T M L A L W K A L P Q G M V A Q K P N V K A E L K V L T R T P V V L A L L T T V L G A G A M F T L Y T Y I A P S L T E F T H A S P T F I T F M L V L I G V G F S
I G N H L G G R F A D L S I N K T L I G F L V L L I V M M V T F P I L A Q S Q I G A A I A L V I W G A A T F A L V P P L Q M R V M S V A H E A P G L A S S V N I G A F N L
G N A V G A A A G A L V L D L G W G Y S A V S F A G A L L A G L G L L L V L F Q I K R E S S P A Q T L Q Q C S D

CASTpFold

Computed Atlas of Surface Topography of the universe of protein Folds

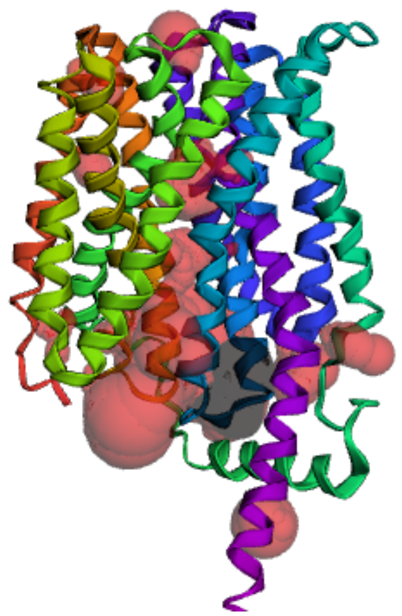

The CASTpFold server is free and open to all users, and there is no login requi[illegible]
Your citation is really important to us. Please cite this paper if you publish or present results us[illegible]
CASTpFold: computed atlas of surface topography of the universe of protein [illegible]
Nucleic Acids Res. 2024. PMID: 38783102. DOI: 10.1093/nar/gkae415.
Bowei Ye, Wei Tian, Boshen Wang, and Jie Liang.

j_68d5b56704513

Take Screenshot Spectrum

Pocket Info

	Pocket ID	Area (SA) (Å^2)	Volume (SA) (Å^3)
-	1	1398.194	1351.629

Show negative volume: Negative volume color: Representation style: Cartoon

Atom Info

Chain	Seq ID	AA	ATOM
A	37	TYR	CE2
A	37	TYR	OH
A	40	ASN	OD1

Pocket ID	Area (SA) ($Å^2$)	Volume (SA) ($Å^3$)

Chain	Seq ID	AA	ATOM
A	40	ASN	ND2
A	41	TYR	CE2
A	41	TYR	OH
A	44	ARG	CZ
A	44	ARG	NH1
A	44	ARG	NH2
A	76	TYR	CE2

< 1 2 3 4 5 ••• 26 >

-	2	116.416	87.466

Show negative volume : Negative volume color : Representation style : Cartoon

Atom Info

Chain	Seq ID	AA	ATOM
A	19	ASN	O
A	22	TYR	C
A	22	TYR	CB
A	23	ARG	N

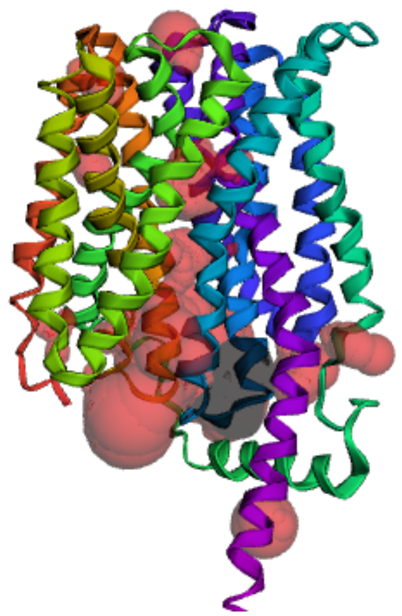

Take Screenshot Spectrum

	Pocket ID	Area (SA) ($Å^2$)	Volume (SA) ($Å^3$)

Chain	Seq ID	AA	ATOM
A	23	ARG	CA
A	23	ARG	CB
A	23	ARG	CG
A	26	VAL	CB
A	26	VAL	CG1
A	26	VAL	CG2

< 1 2 3 4 >

-	3	156.219	58.426

Show negative volume : Negative volume color : Representation style : Cartoon

Atom Info

Chain	Seq ID	AA	ATOM
A	41	TYR	CB
A	41	TYR	CD1
A	42	LEU	CA
A	42	LEU	O
A	42	LEU	CG

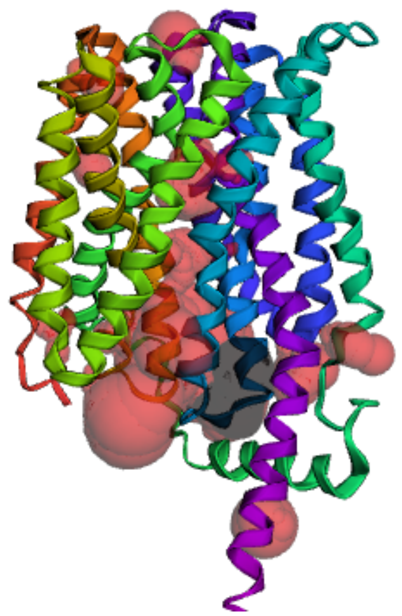

Take Screenshot Spectrum

+

[illegible]	[illegible]	[illegible]

Chain	Seq ID	AA	ATOM
A	45	VAL	O
A	45	VAL	CB
A	45	VAL	CG1
A	45	VAL	CG2
A	46	ASN	CA

< 1 2 3 4 5 >

-	4	67.481	49.231

Show negative volume : Negative volume color : Representation style : Cartoon

Atom Info

Chain	Seq ID	AA	ATOM
A	87	LEU	O
A	87	LEU	CD2
A	88	HIS	CA
A	88	HIS	O
A	89	ARG	O
A	91	GLY	N

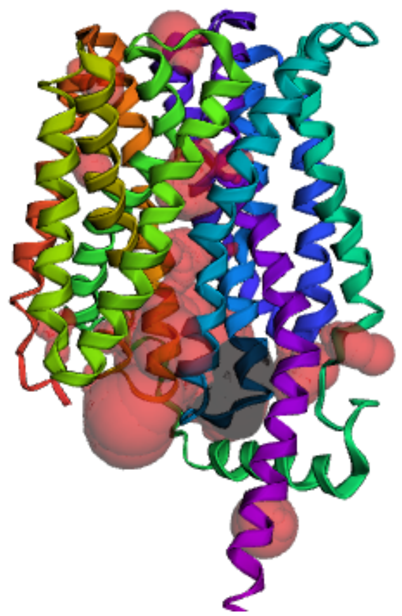

Take Screenshot Spectrum

+

[illegible]	[illegible]	[illegible]

Chain	Seq ID	AA	ATOM
A	91	GLY	CA
A	92	ALA	N
A	92	ALA	CB
A	140	TYR	CD2

< 1 2 3 >

-	5	45.625	24.988

Show negative volume: Negative volume color: Representation style: Cartoon

Atom Info

Chain	Seq ID	AA	ATOM
A	51	LYS	O
A	51	LYS	CG
A	51	LYS	CE
A	52	LEU	CA
A	52	LEU	CD2
A	55	ALA	CB
A	56	ASP	CG

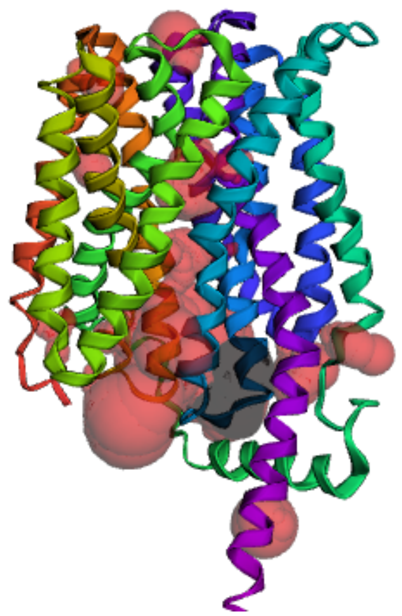

Take Screenshot Spectrum

+

Chain	Seq ID	AA	ATOM
A	56	ASP	OD1
A	60	PHE	O
A	61	SER	C

< 1 2 3 >

	Pocket ID	Area (SA) (Å²)	Volume (SA) (Å³)
-	6	64.009	20.048

Show negative volume: Negative volume color: Representation style: Cartoon

Atom Info

Chain	Seq ID	AA	ATOM
A	277	LEU	CA
A	277	LEU	O
A	277	LEU	CB
A	277	LEU	CD2
A	280	ASP	C
A	280	ASP	CB
A	281	SER	N
A	281	SER	CA

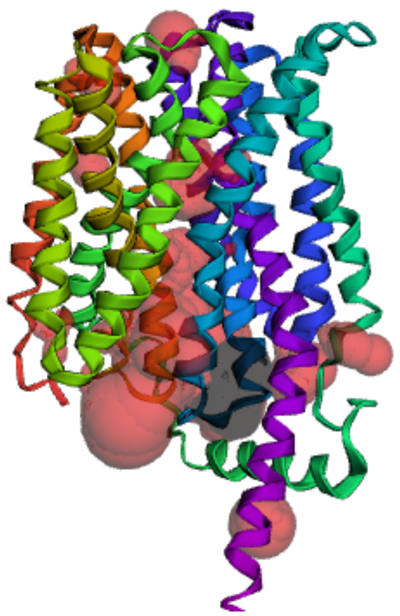

Take Screenshot Spectrum

+

	Pocket ID	Area (SA) [illegible]	Volume (SA) [illegible]

Chain	Seq ID	AA	ATOM
A	281	SER	CB
A	335	SER	C

< 1 2 3 >

-	7	40.585	16.211

Show negative volume: Negative volume color: Representation style: Cartoon

Atom Info

Chain	Seq ID	AA	ATOM
A	28	HIS	CA
A	28	HIS	O
A	28	HIS	CB
A	28	HIS	CD2
A	29	LEU	CA
A	29	LEU	CD2
A	32	LEU	CD2
A	209	LYS	CA
A	209	LYS	O

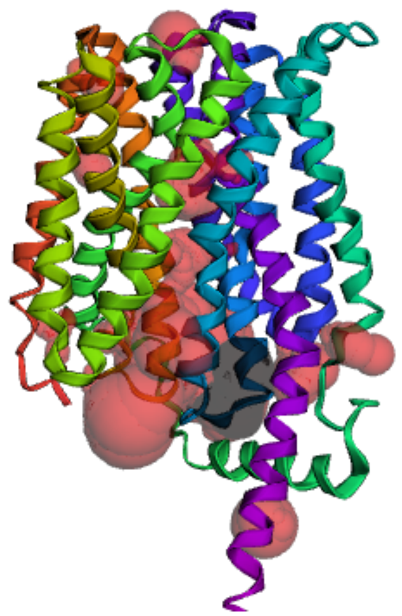

Take Screenshot Spectrum

+

Pocket ID	Area (SA) (Å²)	Volume (SA) (Å³)

Chain	Seq ID	AA	ATOM
A	209	LYS	CB

< 1 2 >

	Pocket ID	Area (SA) (Å²)	Volume (SA) (Å³)
-	8	8.800	9.931

Show negative volume: Negative volume color: Representation style: Cartoon

Atom Info

Chain	Seq ID	AA	ATOM
A	14	GLN	CB
A	14	GLN	CG
A	14	GLN	CD
A	14	GLN	OE1
A	14	GLN	NE2
A	227	ILE	CG1
A	227	ILE	CD1
A	230	THR	CG2

< 1 >

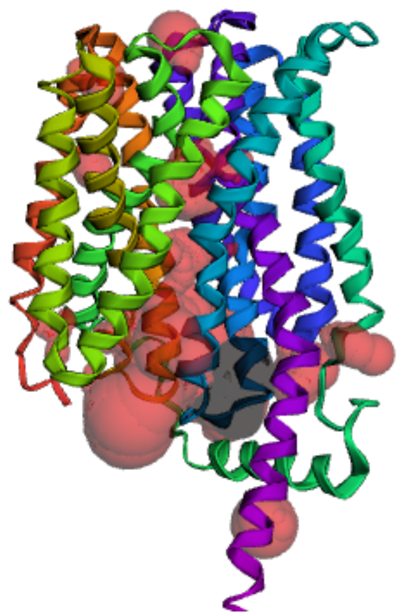

Take Screenshot Spectrum

+

	Pocket ID	Area (SA) ($Å^2$)	Volume (SA) ($Å^3$)
-	9	17.307	5.470

Show negative volume : Negative volume color : Representation style : Cartoon

Atom Info

Chain	Seq ID	AA	ATOM
A	261	PHE	CE1
A	261	PHE	CZ
A	265	SER	CB
A	265	SER	OG
A	393	VAL	CG1
A	394	ALA	N
A	394	ALA	CA
A	394	ALA	CB
A	397	MET	SD
A	397	MET	CE

< 1 2 >

	Pocket ID	Area (SA) ($Å^2$)	Volume (SA) ($Å^3$)
-	10	26.413	3.492

Show negative volume : Negative volume color : Representation style : Cartoon

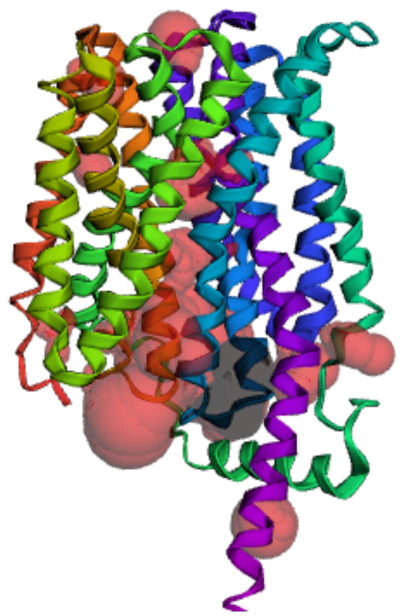

Take Screenshot Spectrum

+

Pocket ID | Area (SA) (A²) | Volume (SA) (A³)

Atom Info

Chain	Seq ID	AA	ATOM
A	252	ARG	CA
A	252	ARG	O
A	252	ARG	CB
A	252	ARG	CG
A	252	ARG	CD
A	252	ARG	NE
A	252	ARG	CZ
A	252	ARG	NH1
A	255	LEU	CD2
A	256	LEU	CG

< 1 2 >

< 1 2 3 4 5 6 >

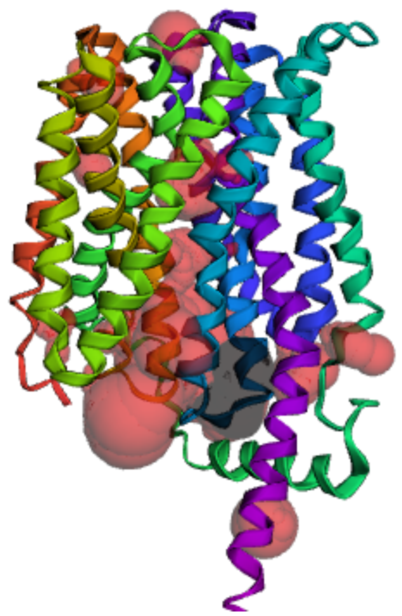

Take Screenshot Spectrum

Sequence info ⓘ

Chain A

Home Compute News About

M S E S S T E S L S Q P T Q L Q E E N N A Y R K T V W H L L P L L M L C Y V L N Y L D R V N V G F A K L Q M A D D L Q F S D T V Y G L G A G M F F I G Y F F L E I P S N L

I L H R V G A R L W I A R I M I T W G I I S A C M A F V T T P M S F Y V M R F L L G V A E A G F F P G I I L Y I S Y

L S G W I M Q Y F N G F Q G Y A G W Q W M F I L E G I P S V L V G I L I I Y K L K D R I T H A T W L T D K E K D I L

L T L I Y F C L I S G F Y T I G F W L P T L I R D S G V K D I L S I G F L T A I P S A A A A V T M I V V S R S A D R

N I V V A M V C L T I G A M G A L S T I P L F W S L P T A F L G G S A A A A G I A F I N S W G N L G G F V A P Y M M

V F L I P A K A V N H

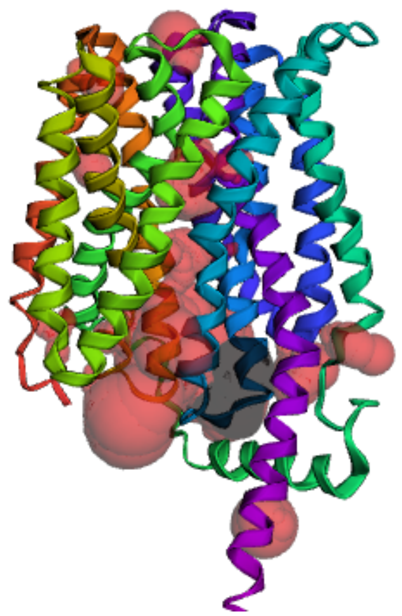

Take Screenshot

Spectrum
