# Supplementary material for: Identification of Opportunistic Pathogens on the Skin of Salamanders for Use as Molecular Targets of a De Novo Design of Multitarget Anti‐Bd Proteins
Source: Int J Microbiol. 2026 Apr 20;2026:5903624. doi: 10.1155/ijm/5903624 (PMC13093180; doi:10.1155/ijm/5903624)
Supplement: Supplementary file 1 — Supporting Information Additional supporting information can be found online in the Supporting Information section. [file IJM-2026-5903624-s001.zip › Possible_Mecanism_of_Action_Proteins_Designed_BD#LC5_5903624.pdf]

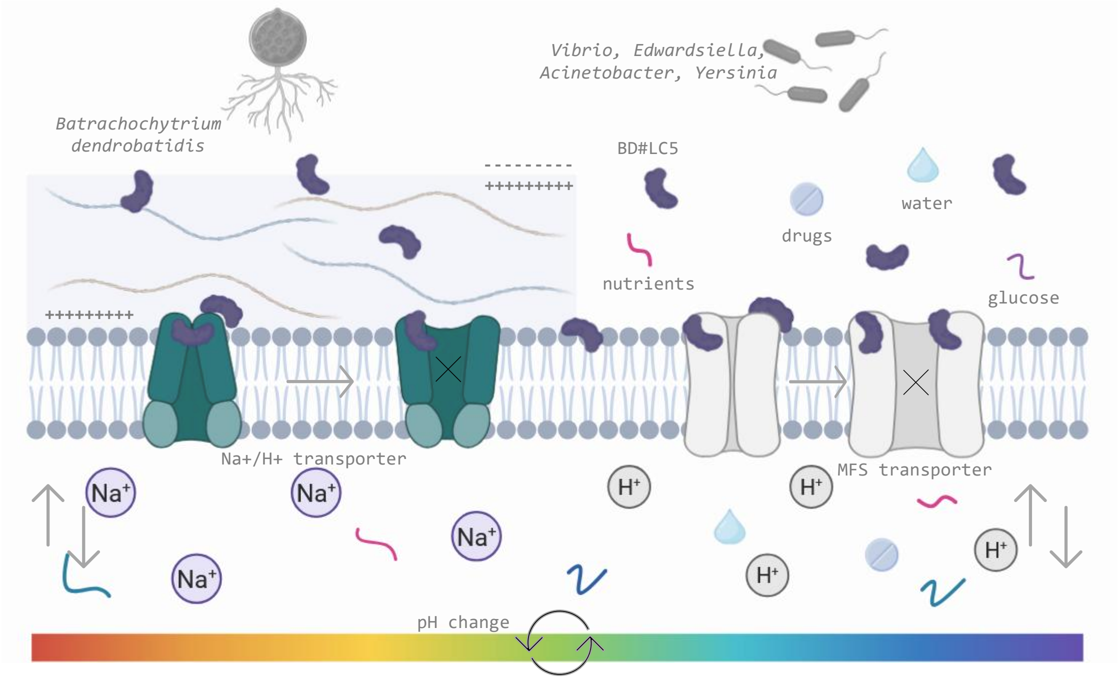

Created with: BioRender: https://app.biorender.com/

1

Figure S3. Expected mode of action of BD#LC5 designs. The following figure illustrates the anticipated mode of action of the designs. First, the anti-Bd protein section is expected to bind primarily through electrostatic interactions to the fungal cell wall, with part of the sequence being specifically designed to insert into and act upon cell walls and membranes, as described in the main manuscript (Shai, 2002). Subsequently, a specific effect is expected on bacterial MFS membrane proteins (Quistgaard *et al.*, 2016) and the Na+/H+ antiporter present in *Batrachochytrium dendrobatidis* (Clemons *et al.*, 2023), through binding to surface cavities on these proteins, which contain specific amino acids predicted to match the sequences present in the design. These channels and membrane proteins in both microorganisms function through conformational changes that allow their opening and closing, thereby enabling the transport of various solutes, nutrients, and ionic exchange to maintain balanced intracellular pH. In bacteria, these channels also function as efflux pumps for various drugs and have been identified as contributors to microbial resistance in some strains (K Redhu *et al.*, 2016). The literature documents that binding of non-specific proteins to such conformationally dependent channels can disrupt their opening or closing mechanisms (Hodi *et al.*, 2010). Proteins of this type (such as the designs proposed here) have been identified in nature as toxins in some cases (Kini & Koh, 2020). The truncated function of these membrane proteins can immediately disrupts osmotic balance, prevents internal pH regulation, impedes the entry of essential solutes and nutrients into the cell, and, most critically, inhibits the efflux of antibiotic or drug molecules toward the extracellular environment. The Figure 3S was created with created with: BioRender: https://app.biorender.com/.

2

Quistgaard, E. M., Löw, C., Guettou, F., & Nordlund, P. (2016). Understanding transport by the major facilitator superfamily (MFS): Structures pave the way. Nature Reviews. Molecular Cell Biology, 17(2), 123-132. https://doi.org/10.1038/nrm.2015.25

K Redhu, A., Shah, A. H., & Prasad, R. (2016). MFS transporters of Candida species and their role in clinical drug resistance. FEMS Yeast Research, 16(4), fow043. https://doi.org/10.1093/femsyr/fow043

Hodi, F. S., O'Day, S. J., McDermott, D. F., Weber, R. W., Sosman, J. A., Haanen, J. B., Gonzalez, R., Robert, C., Schadendorf, D., Hassel, J. C., Akerley, W., van den Eertwegh, A. J. M., Lutzky, J., Lorigan, P., Vaubel, J. M., Linette, G. P., Hogg, D., Ottensmeier, C. H., Lebbé, C., … Urba, W. J. (2010). Improved survival with ipilimumab in patients with metastatic melanoma. The New England Journal of Medicine, 363(8), 711-723. https://doi.org/10.1056/NEJMoa1003466

Kini, R. M., & Koh, C. Y. (2020). Snake venom three-finger toxins and their potential in drug development targeting cardiovascular diseases. Biochemical Pharmacology, 181, 114105. https://doi.org/10.1016/j.bcp.2020.114105
