# Supplementary material for: Identification of Opportunistic Pathogens on the Skin of Salamanders for Use as Molecular Targets of a De Novo Design of Multitarget Anti‐Bd Proteins
Source: Int J Microbiol. 2026 Apr 20;2026:5903624. doi: 10.1155/ijm/5903624 (PMC13093180; doi:10.1155/ijm/5903624)
Supplement: Supplementary file 1 — Supporting Information Additional supporting information can be found online in the Supporting Information section. [file IJM-2026-5903624-s001.zip › Structural_Comparison_AlphaFold_vs_Original_Main_Manuscript_Models_5903624.pdf]

Supplementary material
File: Structural_Comparison_AlphaFold_vs_Original_Main_Manuscript_Models

The following figures show the final results of the structural comparison methods: I-TASSER and trRosetta versus AlphaFold. The same sequences were uploaded to the AlphaFold Server. The reconstructed models are of the original protein receptor in the fungus *Batrachochytrium dendrobatidis*, the section protein with anti-Bd function, and the four MSF protein transporters present in *Vibrio fluvialis*, *Edwardsiella tarda*, *Acinetobacter baumannii*, and *Yersinia pestis*, with respective treatment designs (BD8LC5, BD9LC5, and BD12LC5). The dark display shows all the structural models obtained in AlphaFold.

Next, surface analyses were generated for all the receptors. This analysis indicates probable zones with pockets where interaction with other proteins is possible. These areas are shown with a cartoon surface in gray, green, red, and black. Ten possible positions are shown in each picture and have several matches with the molecular docking results elaborated in HEX Loria and HADDOCK (with the structural models downloaded from AlphaFold). Compare the three results: the surface analysis generated with CASTpFold, the molecular docking results from HEX Loria, and the molecular docking results from HADDOCK with the AlphaFold structural models. This document shows the HADDOCK results, the structural model of all the receptors in blue, and the treatment design in red.

The graphics that follow show a clustering analysis to select the best characteristics of all the measures taken for HADDOCK, as well as the selection of the best cluster with structural models (docking) and quality parameters.

1

Bd Receptor (Transporter) AlphaFold

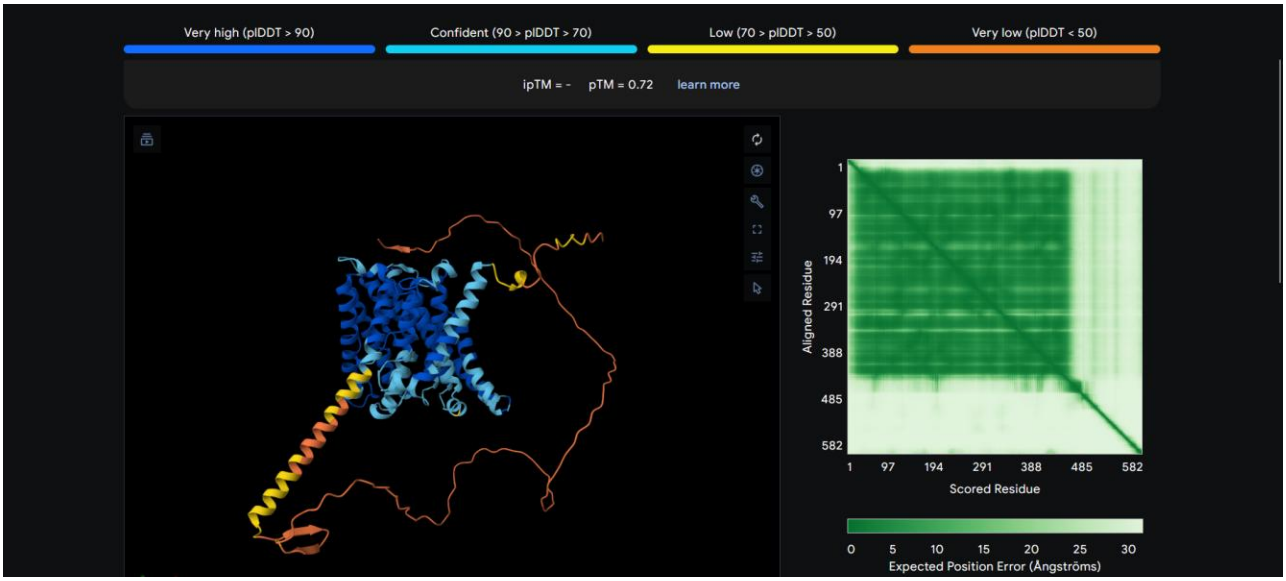

(Abramson *et al.*, 2024)

2

BD-LC5 (protein designed) AlphaFold

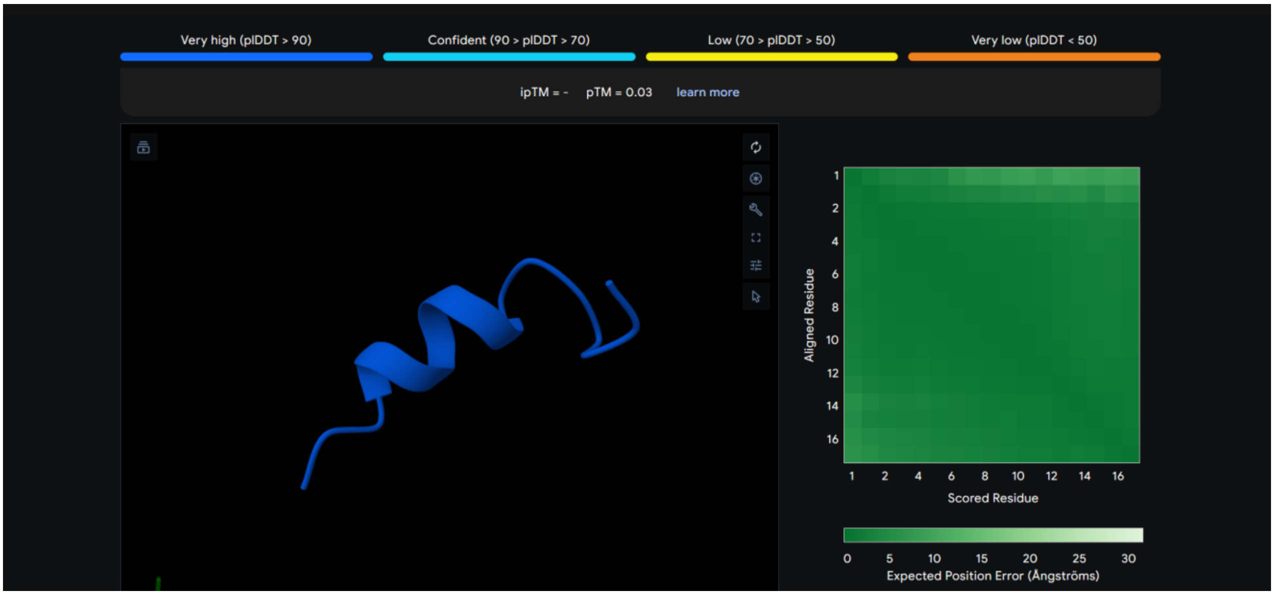

(Abramson *et al.*, 2024)

3

Surface topography analysis: Bd receptor (CASTpFold (2024))

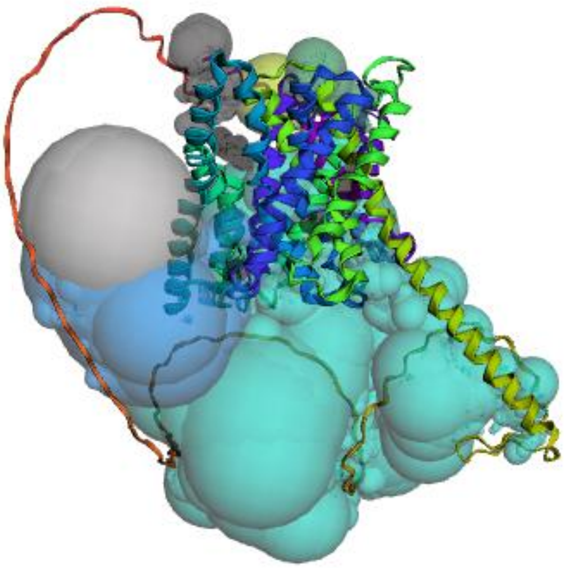

(Ye *et al.*, 2024)

5

BD-receptor and LC5 protein (HADDOCK)

Cluster 2

HADDOCK score	-92.8 +/- 5.8
Cluster size	26
RMSD from the overall lowest-energy structure	0.6 +/- 0.4
Van der Waals energy	-39.0 +/- 2.1
Electrostatic energy	-273.9 +/- 28.2
Desolvation energy	-1.8 +/- 2.3
Restraints violation energy	28.4 +/- 14.5
Buried Surface Area	1434.6 +/- 112.4
Z-Score	-1.5

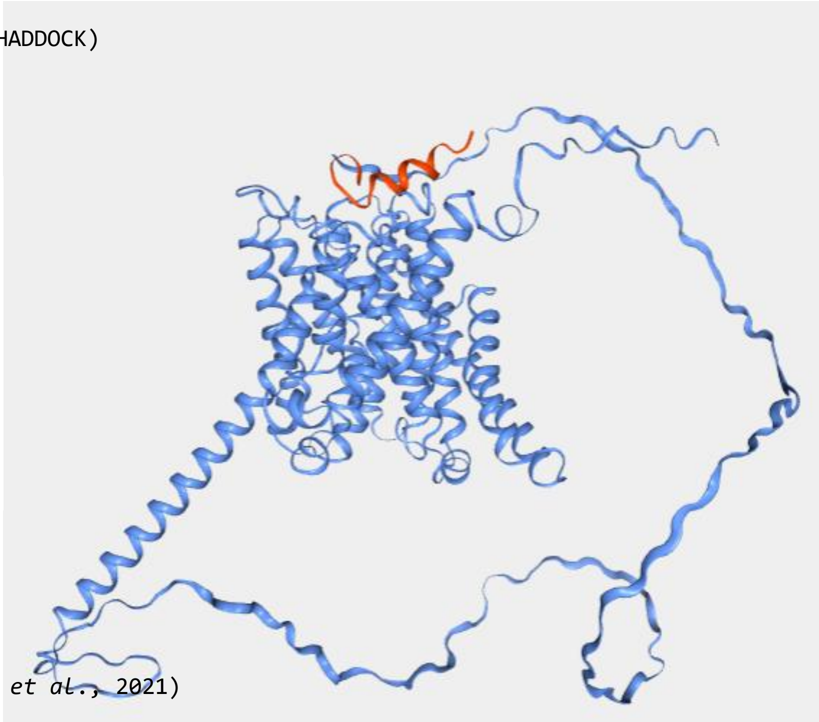

(Honorato *et al.*, 2024; Honorato *et al.*, 2021)

6

BD-receptor and LC5 protein (HADDOCK)

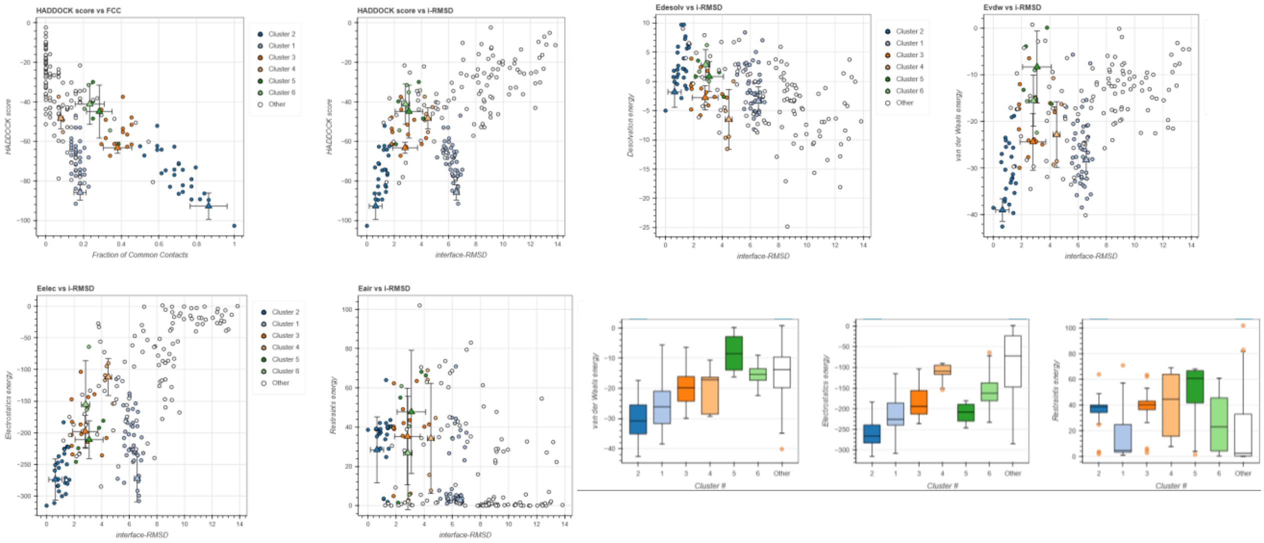

(Honorato *et al.*, 2024; Honorato *et al.*, 2021)

7

BD8LC5 (AlphaFold)

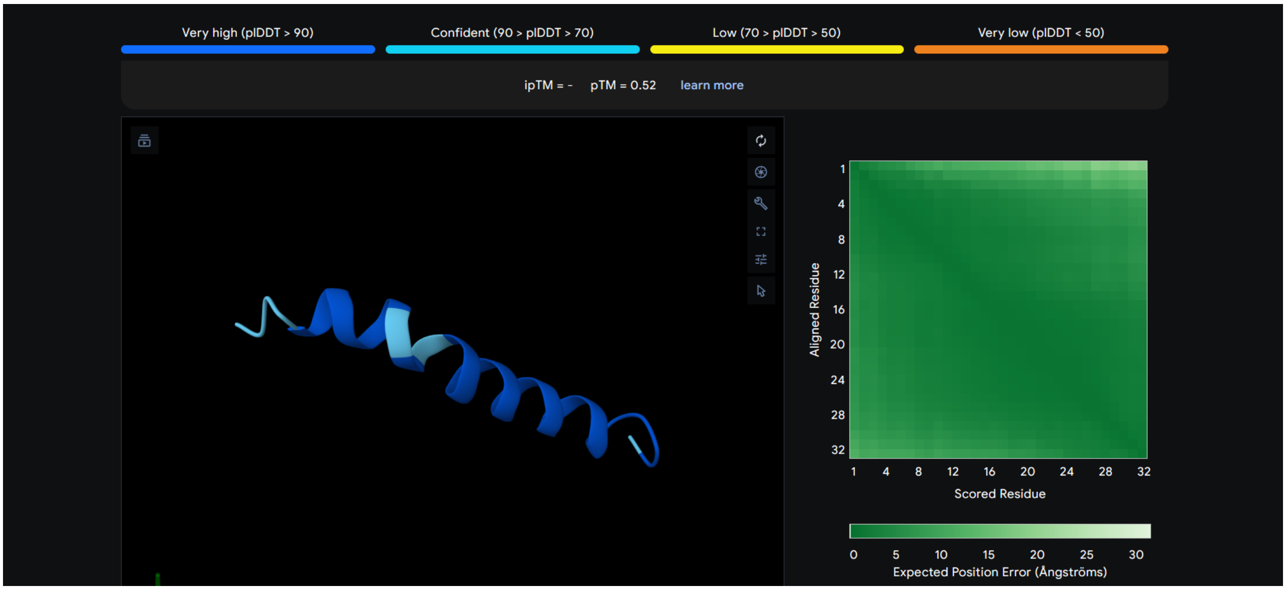

(Abramson *et al.*, 2024)

9

BD9LC5 (AlphaFold)

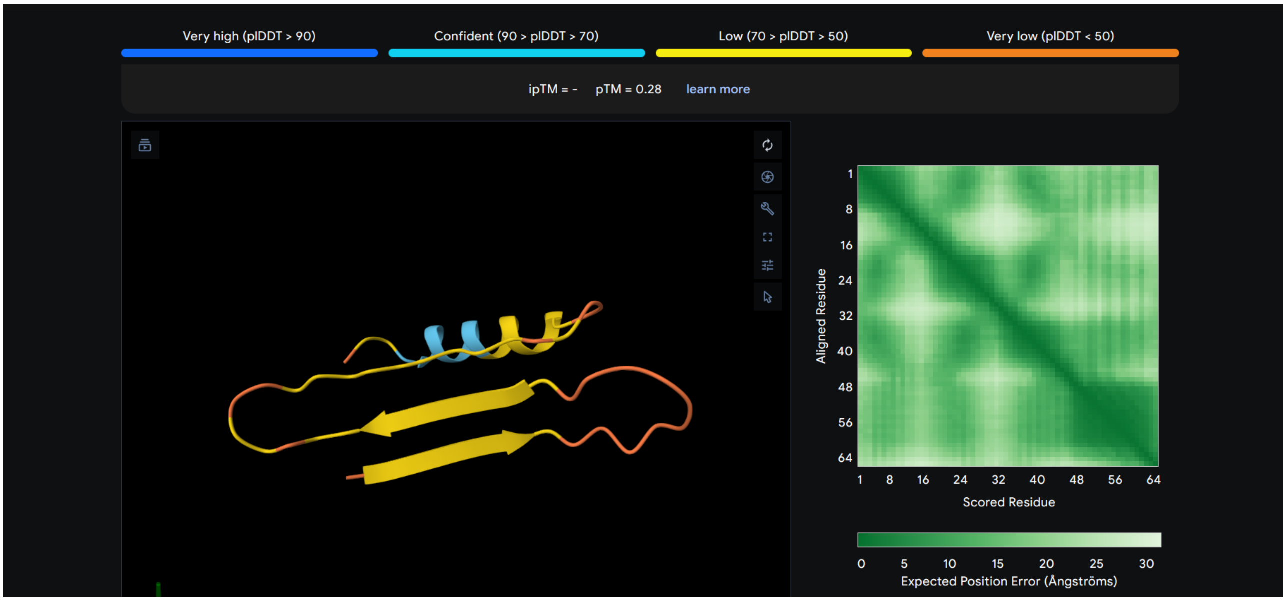

(Abramson *et al.*, 2024)

10

BD12LC5 (ALphaFold)

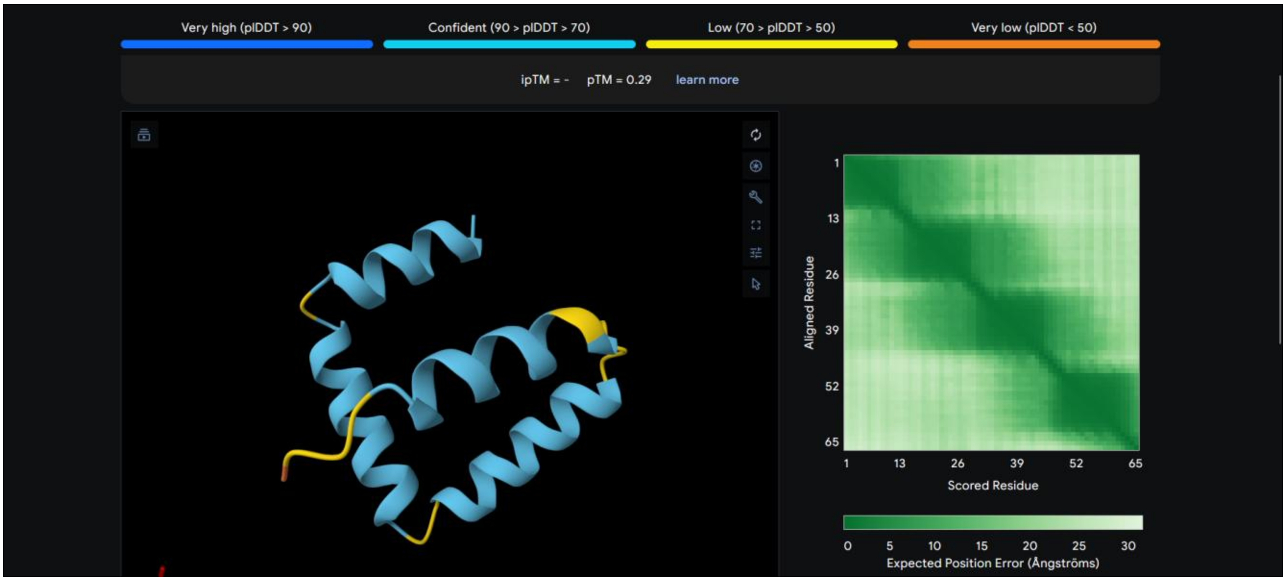

(Abramson *et al.*, 2024)

11

MSF *Vibrio fluvialis* (AlphaFold)

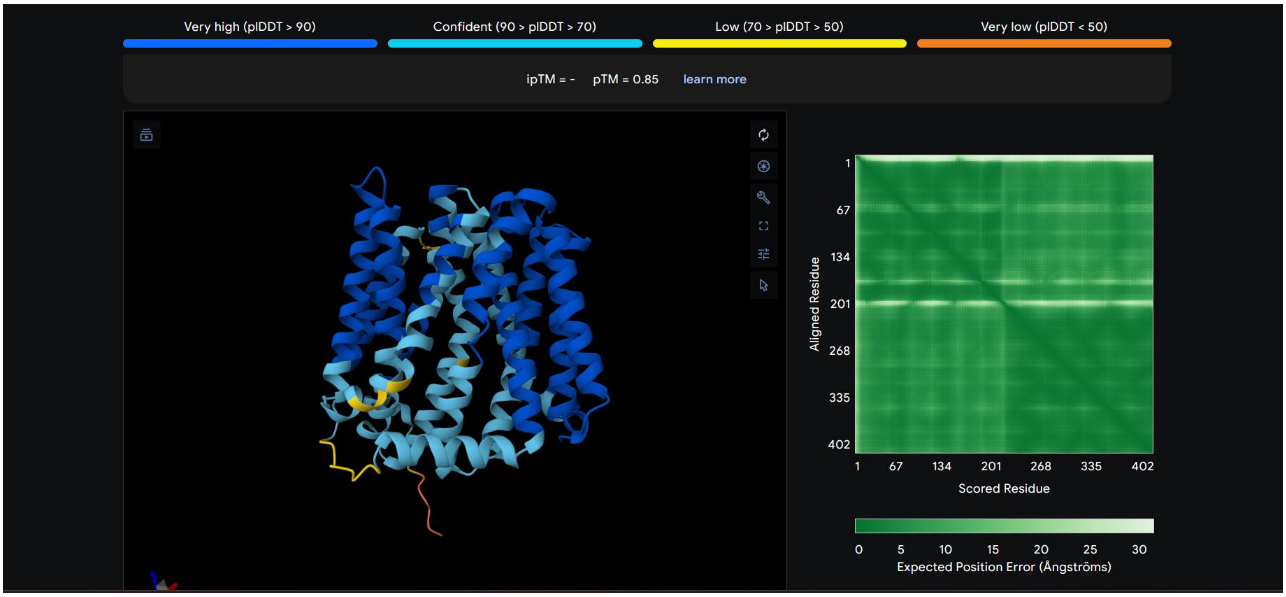

(Abramson *et al.*, 2024)

13

Surface topography analysis: MSF *Vibrio fluvialis* (CASTpFold (2024))

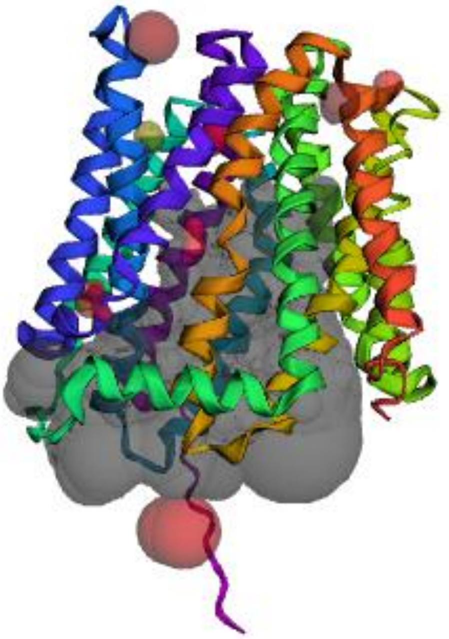

(Ye *et al.*, 2024)

14

MSF *Edwardsiella tarda* AlphaFold

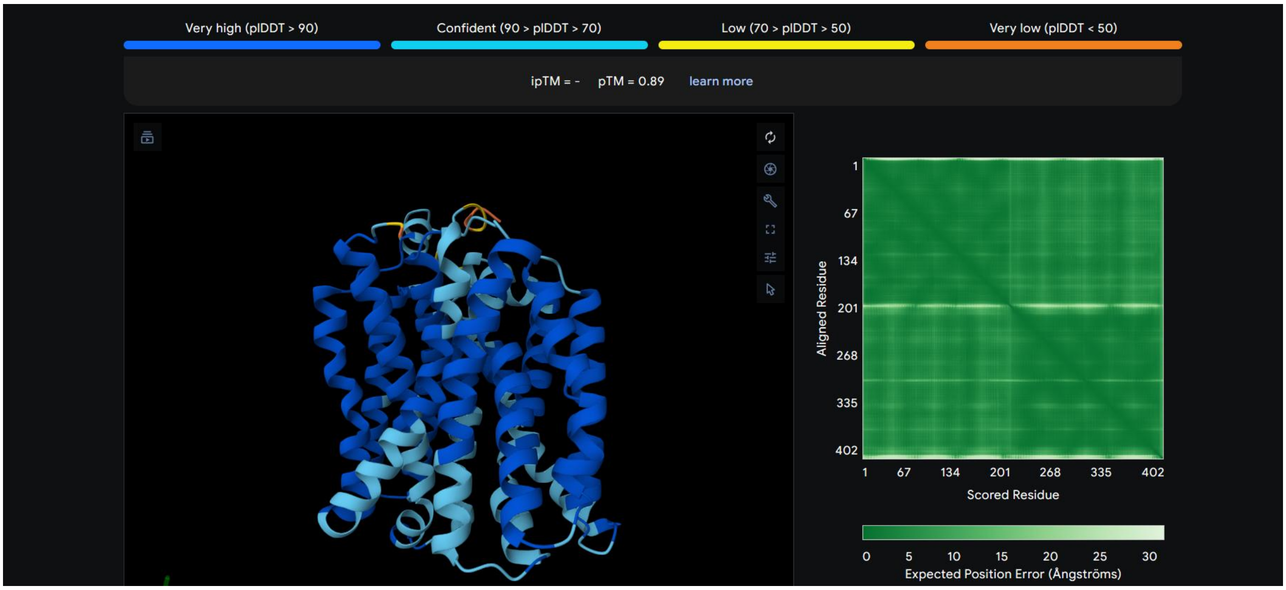

(Abramson *et al.*, 2024)

16

Surface topography analysis:
MSF *Edwardsiella tarda*
(CASTpFold 2024)

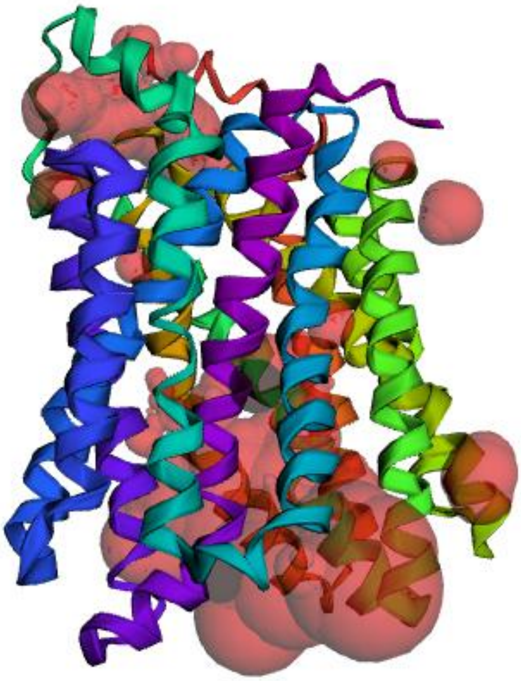

(Ye *et al.*, 2024)

17

MSF *Acinetobacter baumannii* (AlphaFold)

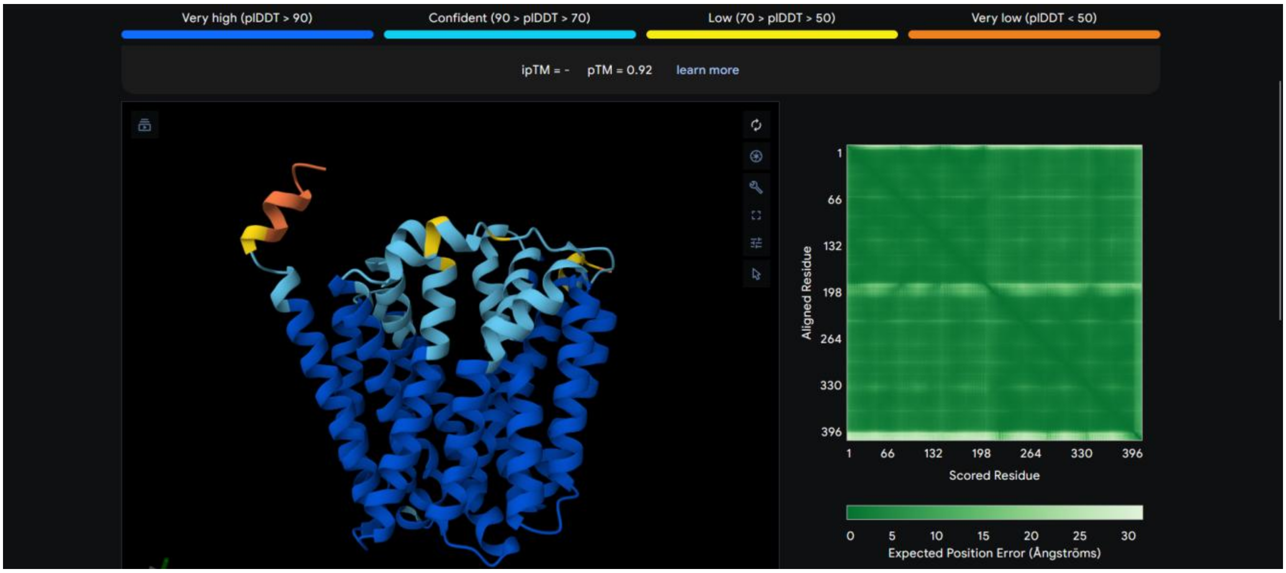

(Abramson *et al.*, 2024)

19

Surface topography analysis: MSF *Acinetobacter baumannii* (CASTpFold 2024)

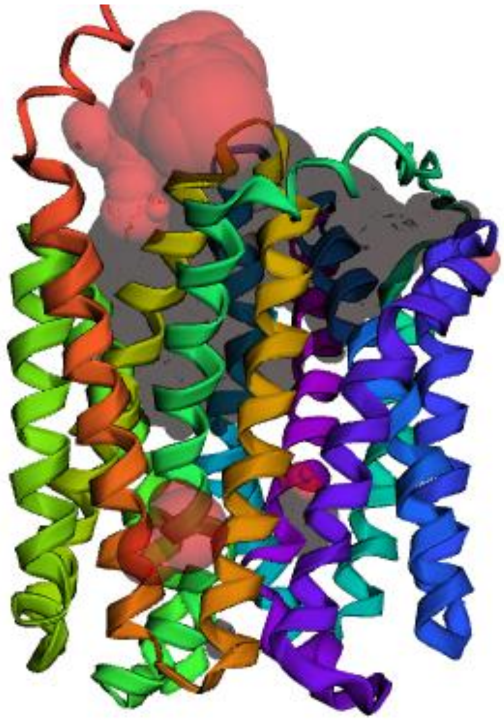

(Ye *et al.*, 2024)

20

MSF *Yersinia pestis* (AlphaFold)

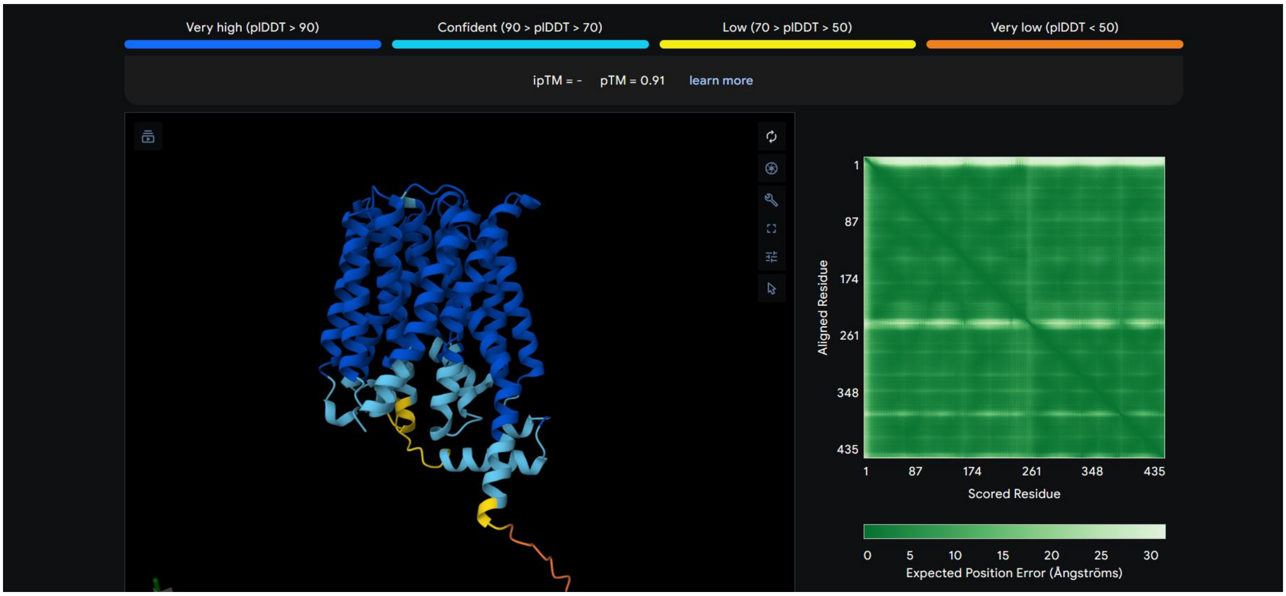

(Abramson *et al.*, 2024)

22

Surface topography analysis: MSF
Yersinia pestis (CASTpFold 2024)

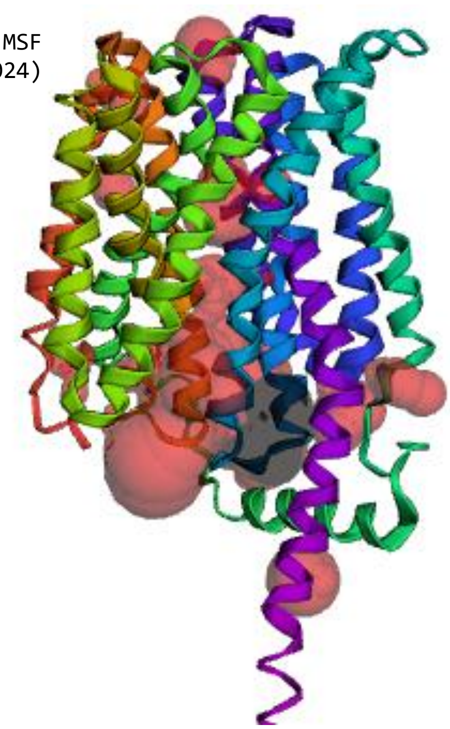

(Ye *et al.*, 2024)

23

MSF- *Vibrio fluvialis* and BD8LC5 HADDOCK

Cluster 1

HADDOCK score	-90.1 +/- 4.0
Cluster size	22
RMSD from the overall lowest-energy structure	1.0 +/- 0.7
Van der Waals energy	-35.6 +/- 2.5
Electrostatic energy	-194.1 +/- 15.1
Desolvation energy	-28.7 +/- 2.9
Restraints violation energy	130.1 +/- 40.5
Buried Surface Area	1306.5 +/- 51.7
Z-Score	-2.1

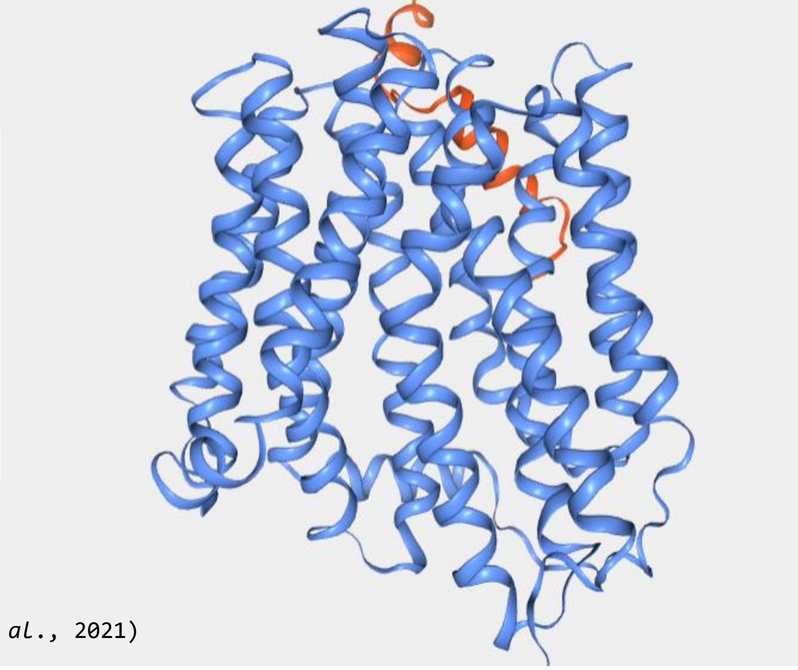

(Honorato *et al.*, 2024; Honorato *et al.*, 2021)

25

MSF- *Vibrio fluvialis* and BD8LC5 (HADDOCK)

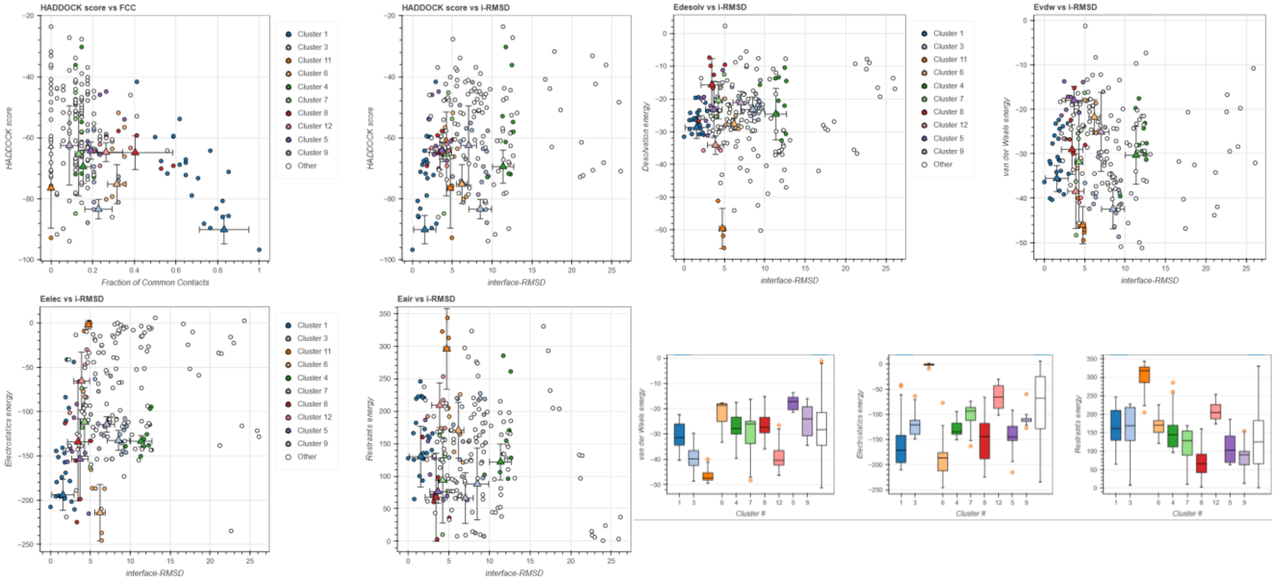

(Honorato *et al.*, 2024; Honorato *et al.*, 2021)

26

MSF- *Edwarsiella tarda* and BD8LC5 (HADDOCK)

Cluster 2

HADDOCK score	-87.2 +/- 4.2
Cluster size	16
RMSD from the overall lowest-energy structure	7.9 +/- 0.1
Van der Waals energy	-52.3 +/- 4.0
Electrostatic energy	-24.0 +/- 5.2
Desolvation energy	-47.3 +/- 1.4
Restraints violation energy	171.8 +/- 67.0
Buried Surface Area	1643.3 +/- 116.7
Z-Score	-1.7

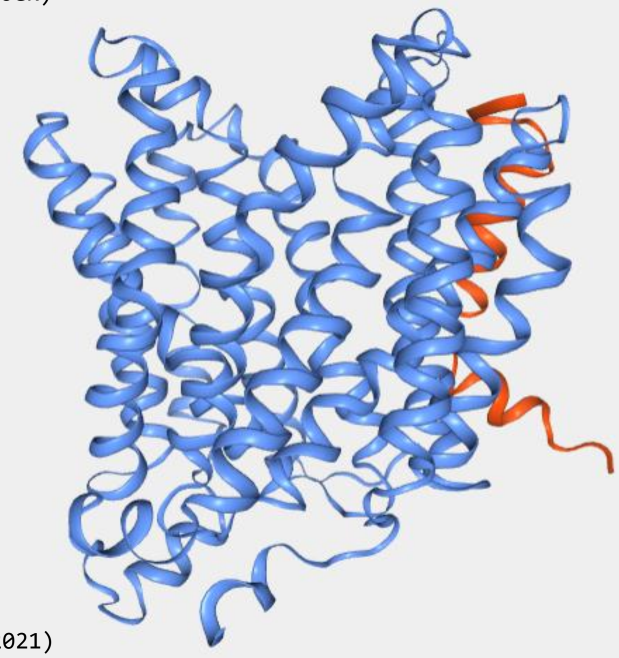

(Honorato *et al.*, 2024; Honorato *et al.*, 2021)

27

MSF- *Edwardsiella tarda* and BD8LC5 (HADDOCK)

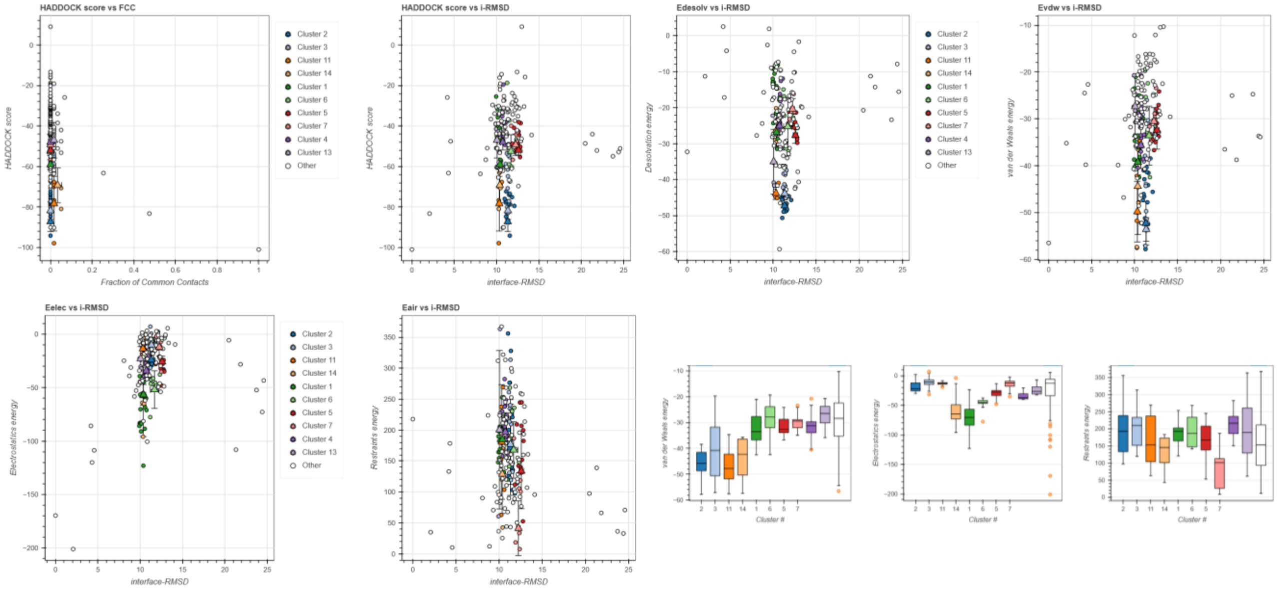

(Honorato *et al.*, 2024; Honorato *et al.*, 2021)

28

MSF- *Acinetobacter baumannii* and BD8LC5 (HADDOCK)

Cluster 2

HADDOCK score	-90.1 +/- 4.7
Cluster size	12
RMSD from the overall lowest-energy structure	6.8 +/- 0.1
Van der Waals energy	-59.6 +/- 4.4
Electrostatic energy	-278.8 +/- 42.3
Desolvation energy	-5.2 +/- 2.7
Restraints violation energy	304.4 +/- 85.6
Buried Surface Area	2041.2 +/- 54.0
Z-Score	-2.0

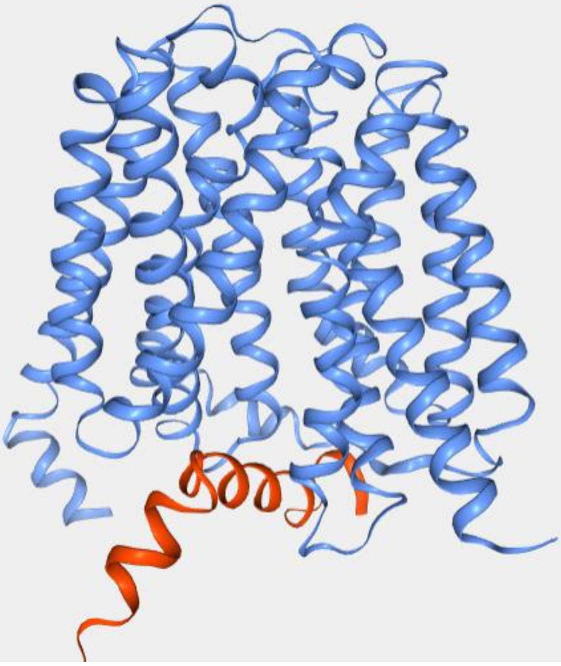

(Honorato *et al.*, 2024; Honorato *et al.*, 2021)

29

MSF- *Acinetobacter baumannii* and BD8LC5 (HADDOCK)

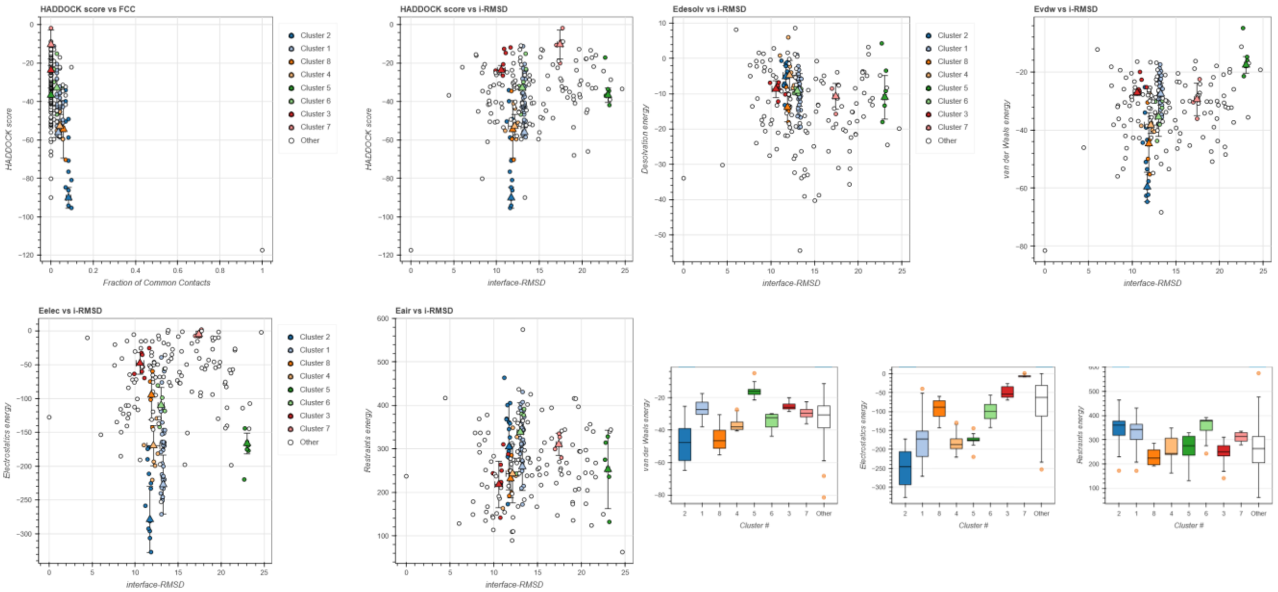

(Honorato *et al.*, 2024; Honorato *et al.*, 2021)

30

MSF- *Yersinia pestis* and BD8LC5 (HADDOCK)

Cluster 12

HADDOCK score	-63.9 +/- 6.0
Cluster size	4
RMSD from the overall lowest-energy structure	13.9 +/- 0.1
Van der Waals energy	-53.9 +/- 4.9
Electrostatic energy	-37.3 +/- 19.0
Desolvation energy	-34.6 +/- 3.5
Restraints violation energy	319.9 +/- 56.8
Buried Surface Area	1510.7 +/- 115.5
Z-Score	-1.7

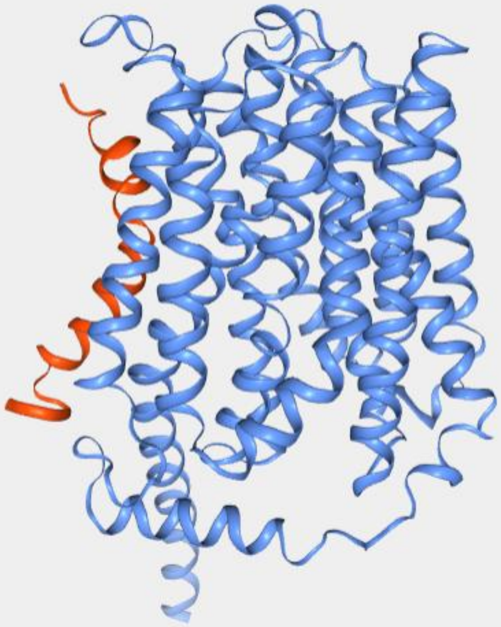

(Honorato *et al.*, 2024; Honorato *et al.*, 2021)

31

MSF- *Yersinia pestis* and BD8LC5 (HADDOCK)

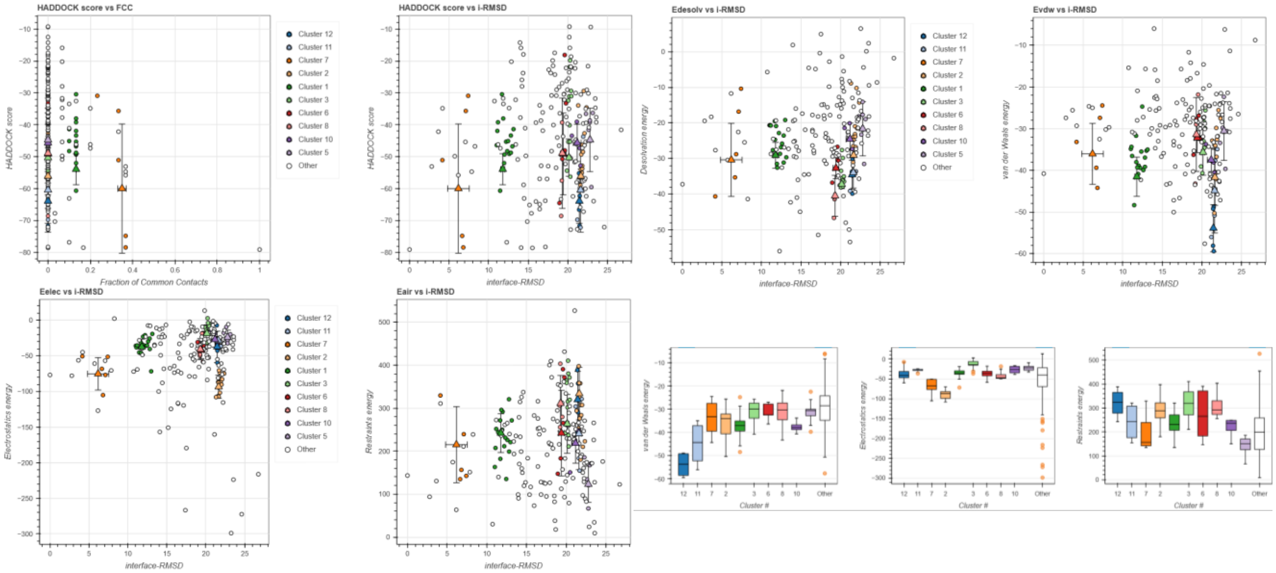

(Honorato *et al.*, 2024; Honorato *et al.*, 2021)

32

MSF- *Vibrio fluvialis* and BD9LC5 (HADDOCK)

Cluster 3

HADDOCK score	-94.4 +/- 3.5
Cluster size	14
RMSD from the overall lowest-energy structure	2.5 +/- 0.1
Van der Waals energy	-66.9 +/- 5.3
Electrostatic energy	-61.2 +/- 12.4
Desolvation energy	-42.1 +/- 2.6
Restraints violation energy	267.9 +/- 76.3
Buried Surface Area	2084.5 +/- 49.6
Z-Score	-1.9

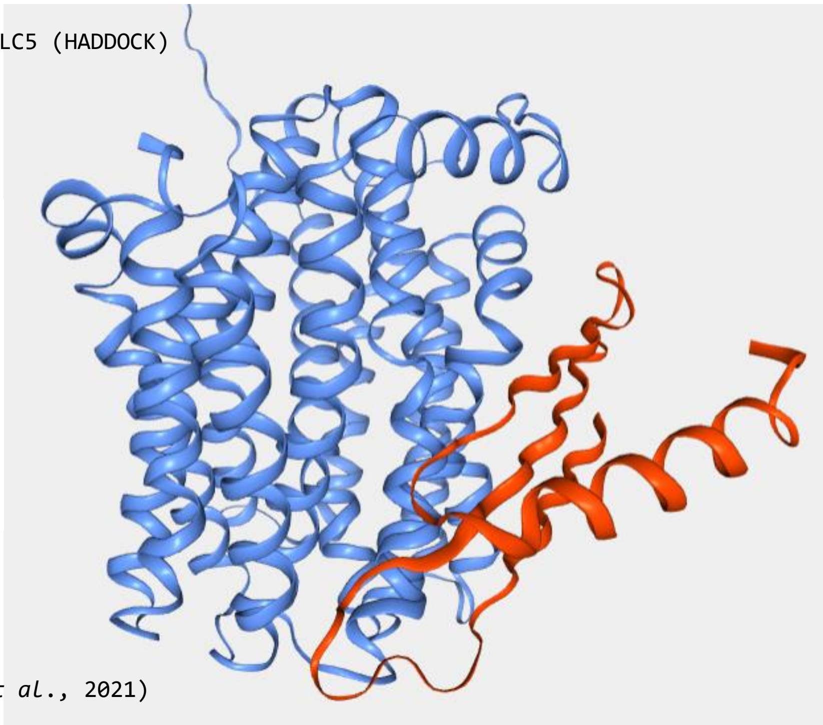

(Honorato *et al.*, 2024; Honorato *et al.*, 2021)

34

MSF- *Vibrio fluvialis* and BD9LC5 (HADDOCK)

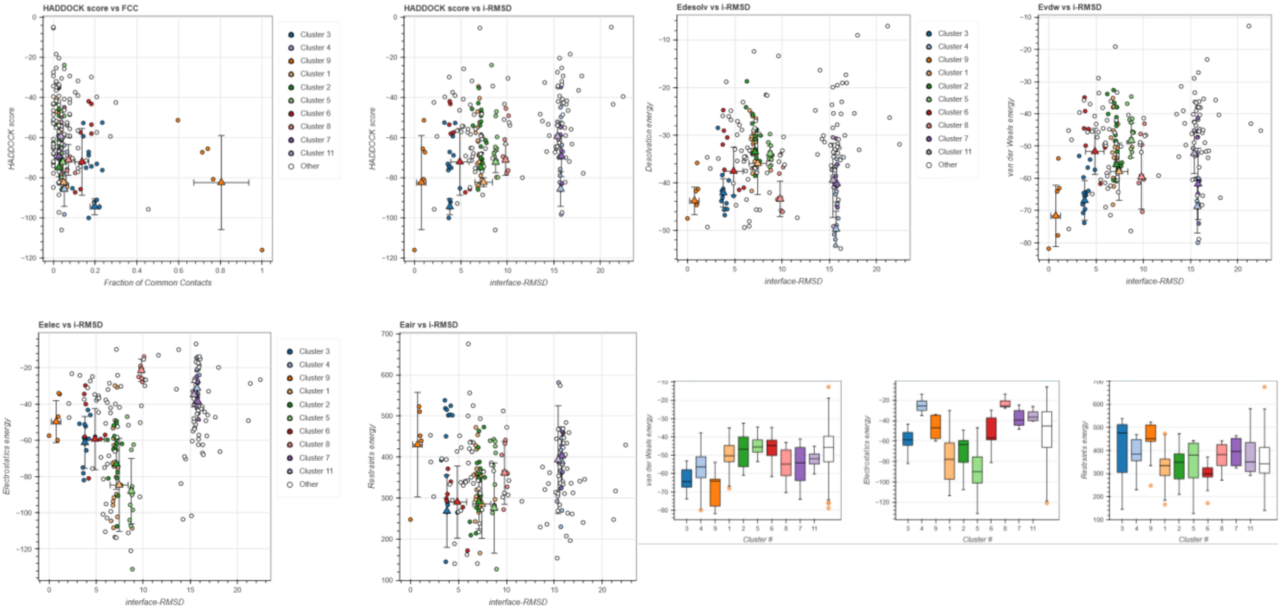

(Honorato *et al.*, 2024; Honorato *et al.*, 2021)

35

MSF- *Edwardsiella tarda* and BD9LC5 (HADDOCK)

Cluster 1

HADDOCK score	-69.3 +/- 17.7
Cluster size	11
RMSD from the overall lowest-energy structure	1.2 +/- 0.8
Van der Waals energy	-58.0 +/- 9.3
Electrostatic energy	-16.7 +/- 9.8
Desolvation energy	-46.7 +/- 5.7
Restraints violation energy	386.4 +/- 75.8
Buried Surface Area	2045.1 +/- 174.6
Z-Score	-2.0

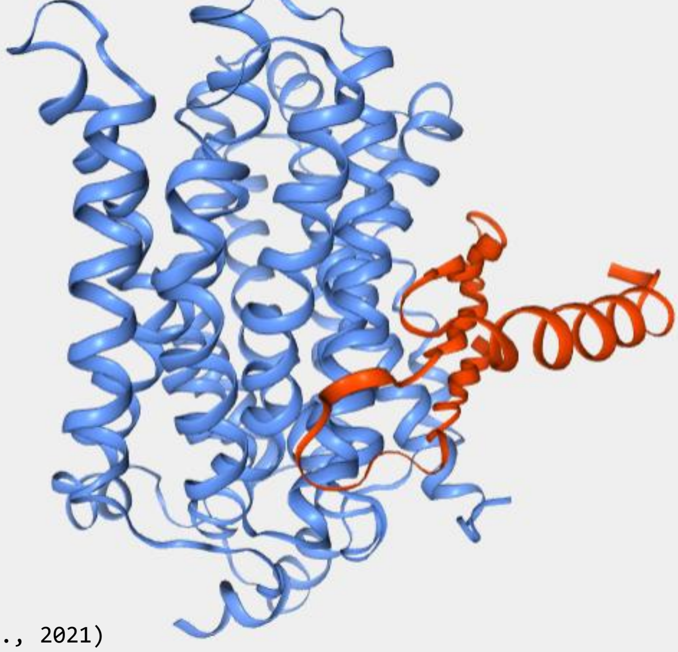

(Honorato *et al.*, 2024; Honorato *et al.*, 2021)

36

MSF- *Edwardsiella tarda* and BD9LC5 (HADDOCK)

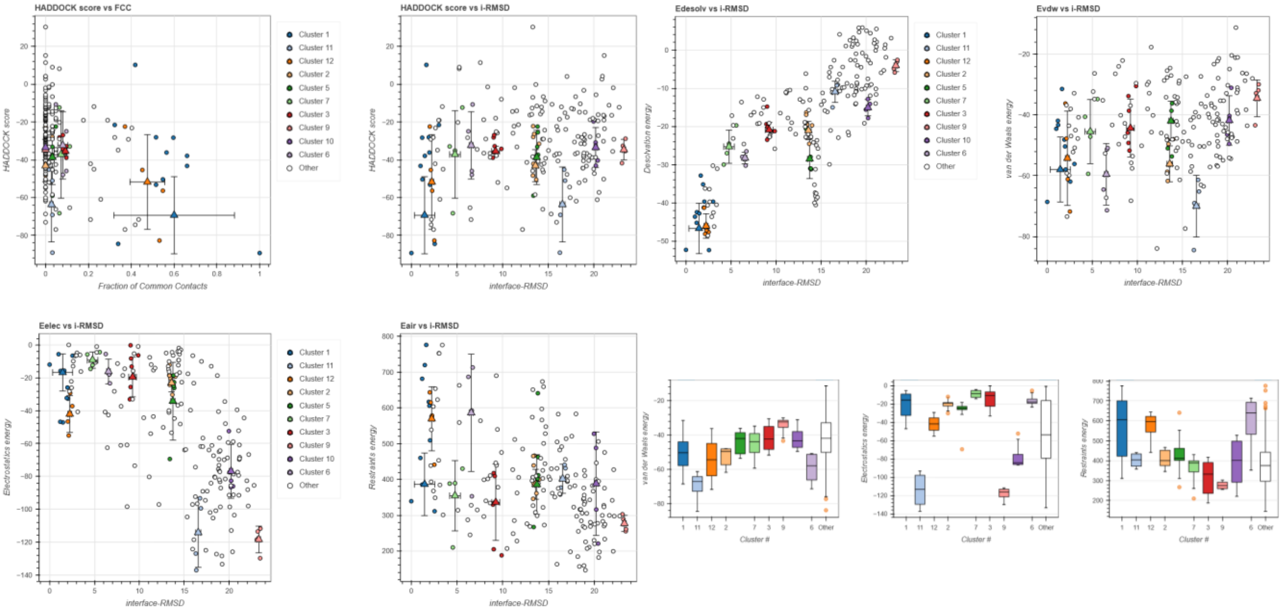

(Honorato *et al.*, 2024; Honorato *et al.*, 2021)

37

MSF- *Acinetobacter baumannii* and BD9LC5 (HADDOCK)

Cluster 7

HADDOCK score	-66.3 +/- 21.4
Cluster size	4
RMSD from the overall lowest-energy structure	1.0 +/- 0.9
Van der Waals energy	-69.4 +/- 8.3
Electrostatic energy	-85.9 +/- 4.5
Desolvation energy	-27.4 +/- 4.3
Restraints violation energy	476.7 +/- 108.9
Buried Surface Area	2254.3 +/- 95.0
Z-Score	-1.7

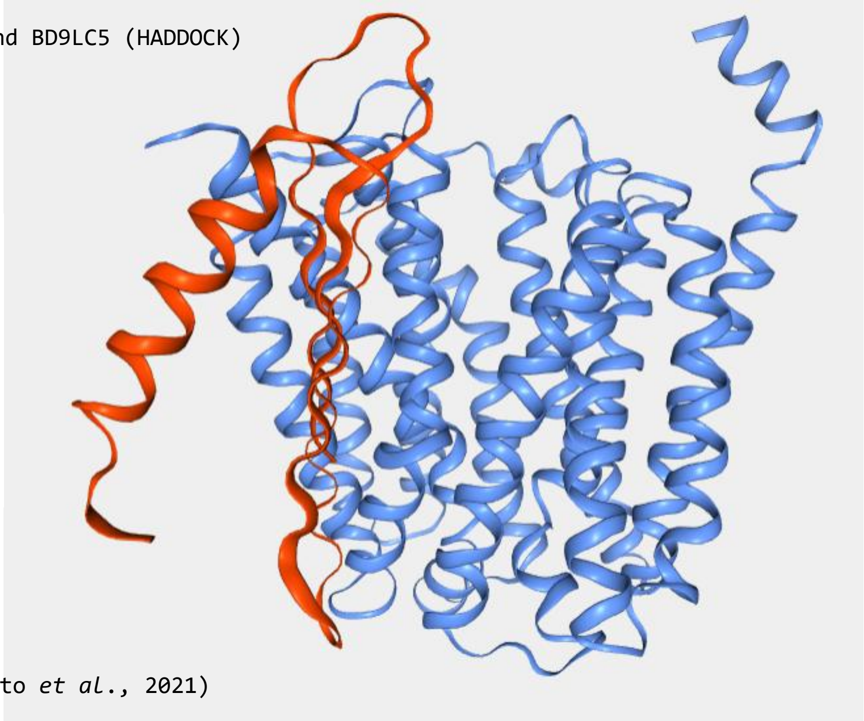

(Honorato *et al.*, 2024; Honorato *et al.*, 2021)

38

MSF- *Acinetobacter baumannii* and BD9LC5 (HADDOCK)

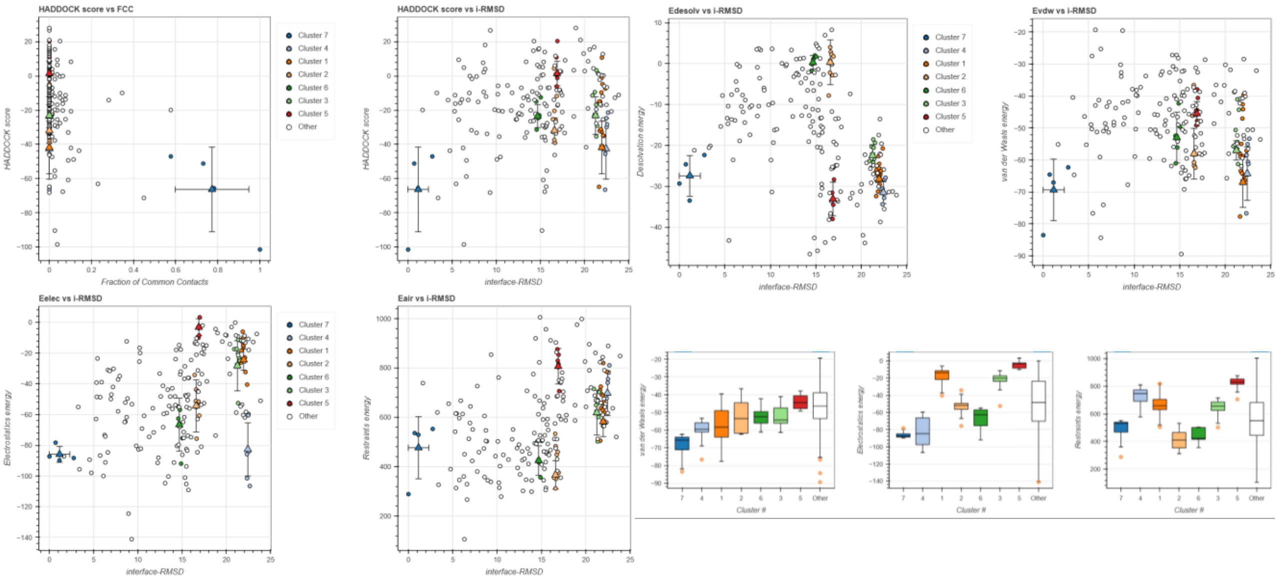

(Honorato *et al.*, 2024; Honorato *et al.*, 2021)

39

MSF- *Yersinia pestis* and BD9LC5 (HADDOCK)

Cluster 4

HADDOCK score	-43.9 +/- 8.3
Cluster size	9
RMSD from the overall lowest-energy structure	7.4 +/- 0.2
Van der Waals energy	-69.9 +/- 6.8
Electrostatic energy	-95.8 +/- 23.4
Desolvation energy	-4.9 +/- 1.5
Restraints violation energy	500.6 +/- 41.7
Buried Surface Area	1997.5 +/- 167.1
Z-Score	-2.4

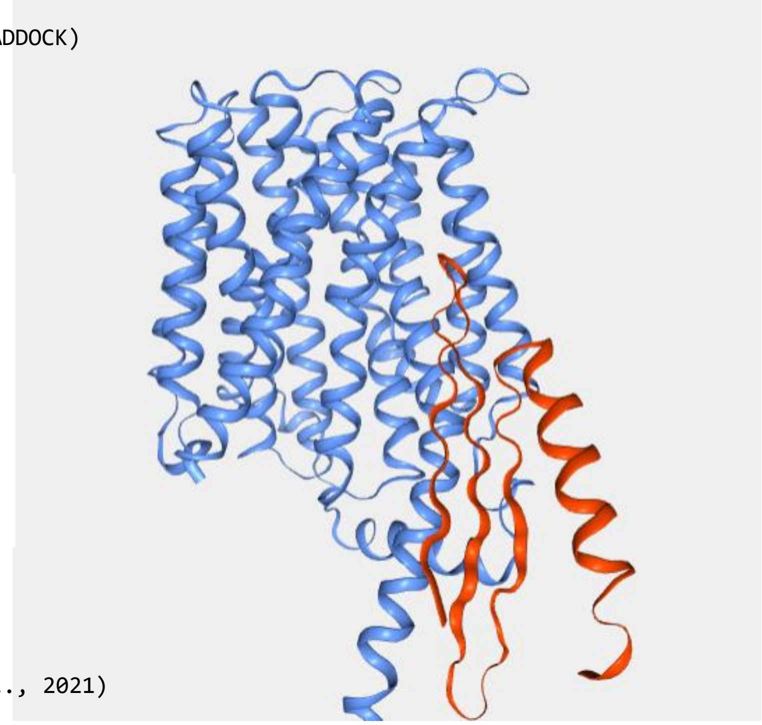

(Honorato *et al.*, 2024; Honorato *et al.*, 2021)

40

MSF- *Yersinia pestis* and BD9LC5 (HADDOCK)

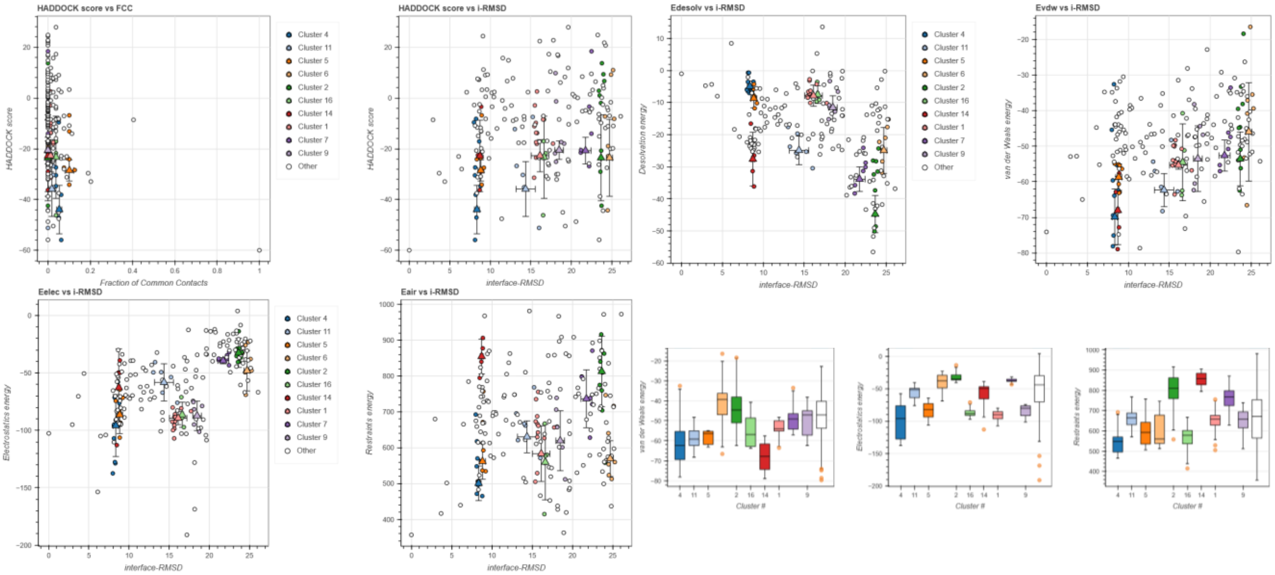

(Honorato *et al.*, 2024; Honorato *et al.*, 2021)

41

MSF- *Vibrio fluvialis* and BD12LC5 (HADDOCK)

Cluster 3

HADDOCK score	-251.1 +/- 9.6
Cluster size	11
RMSD from the overall lowest-energy structure	0.4 +/- 0.2
Van der Waals energy	-126.5 +/- 9.4
Electrostatic energy	-35.1 +/- 8.7
Desolvation energy	-175.2 +/- 6.7
Restraints violation energy	576.6 +/- 147.9
Buried Surface Area	3560.0 +/- 168.5
Z-Score	-2.3

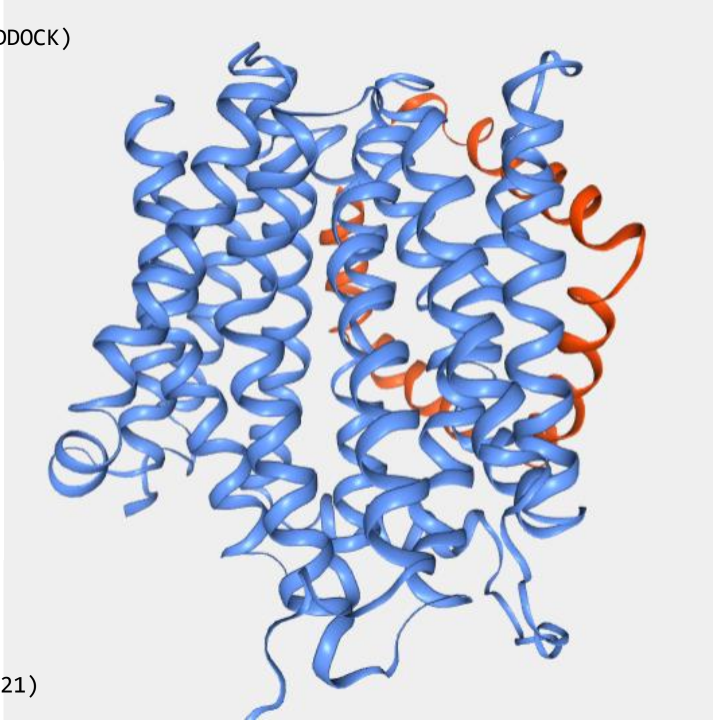

(Honorato *et al.*, 2024; Honorato *et al.*, 2021)

43

MSF- *Vibrio fluvialis* and BD12LC5 (HADDOCK)

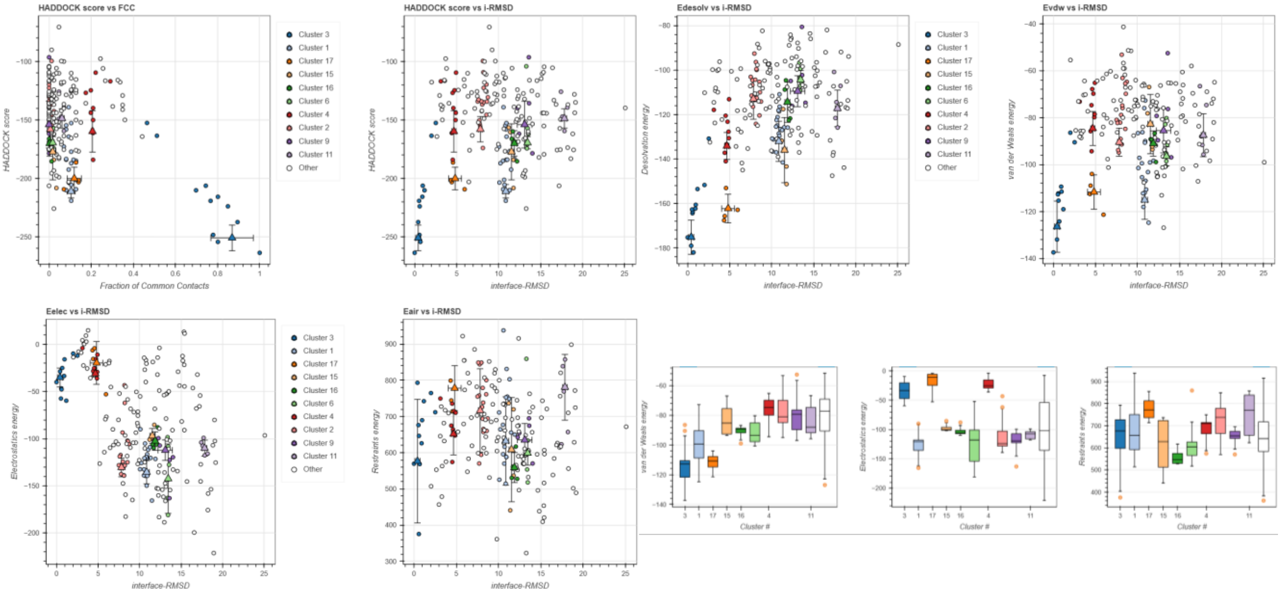

(Honorato *et al.*, 2024; Honorato *et al.*, 2021)

44

MSF- *Edwardsiella tarda* and BD12LC5 (HADDOCK)

Cluster 10

HADDOCK score	-179.5 +/- 5.9
Cluster size	6
RMSD from the overall lowest-energy structure	0.6 +/- 0.3
Van der Waals energy	-112.9 +/- 7.5
Electrostatic energy	-50.2 +/- 5.6
Desolvation energy	-136.6 +/- 4.4
Restraints violation energy	801.2 +/- 69.6
Buried Surface Area	3233.5 +/- 67.6
Z-Score	-2.4

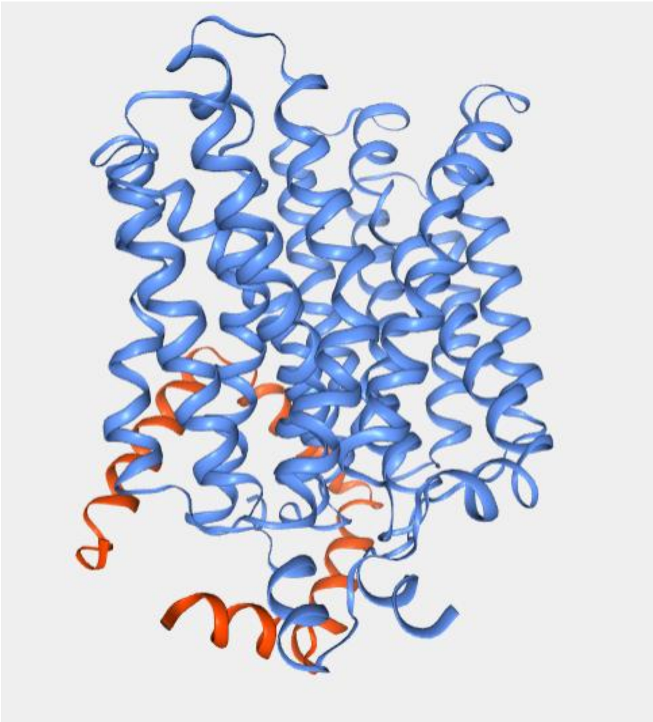

(Honorato *et al.*, 2024; Honorato *et al.*, 2021)

45

MSF- *Edwardsiella tarda* and BD12LC5 (HADDOCK)

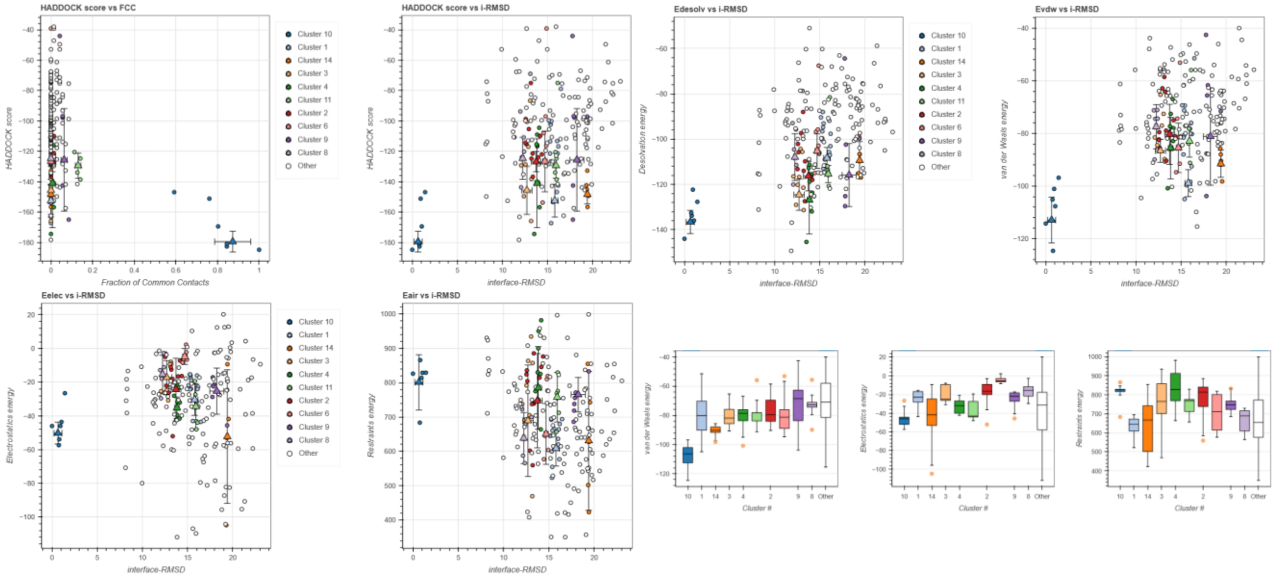

(Honorato *et al.*, 2024; Honorato *et al.*, 2021)

46

MSF- *Acinetobacter baumannii* and BD12LC5 (HADDOCK)

Cluster 2

HADDOCK score	-160.9 +/- 10.3
Cluster size	8
RMSD from the overall lowest-energy structure	7.5 +/- 0.3
Van der Waals energy	-100.2 +/- 10.1
Electrostatic energy	-50.0 +/- 11.2
Desolvation energy	-127.6 +/- 5.0
Restraints violation energy	769.5 +/- 38.1
Buried Surface Area	2896.7 +/- 99.8
Z-Score	-1.6

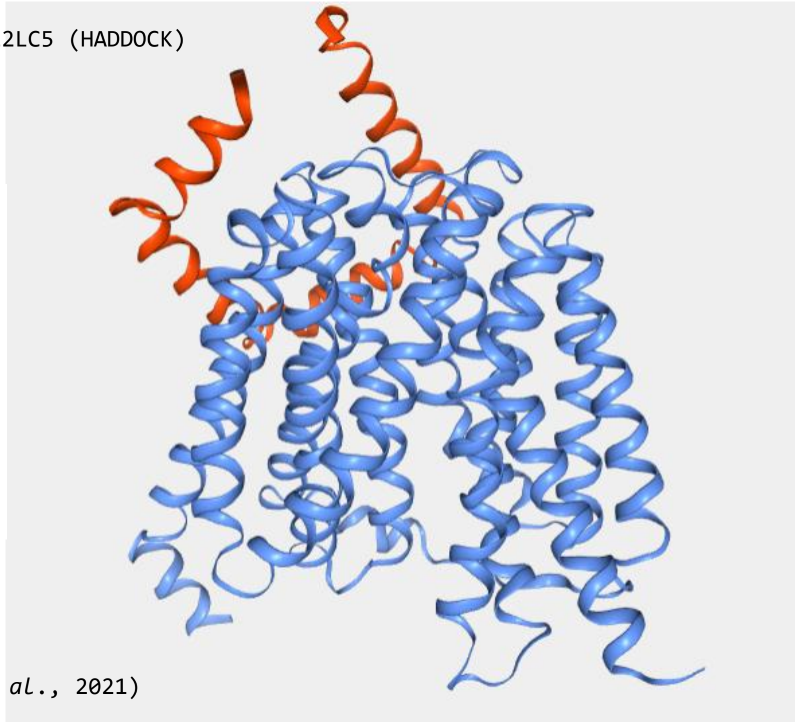

(Honorato *et al.*, 2024; Honorato *et al.*, 2021)

47

MSF- *Acinetobacter baumannii* and BD12LC5 (HADDOCK)

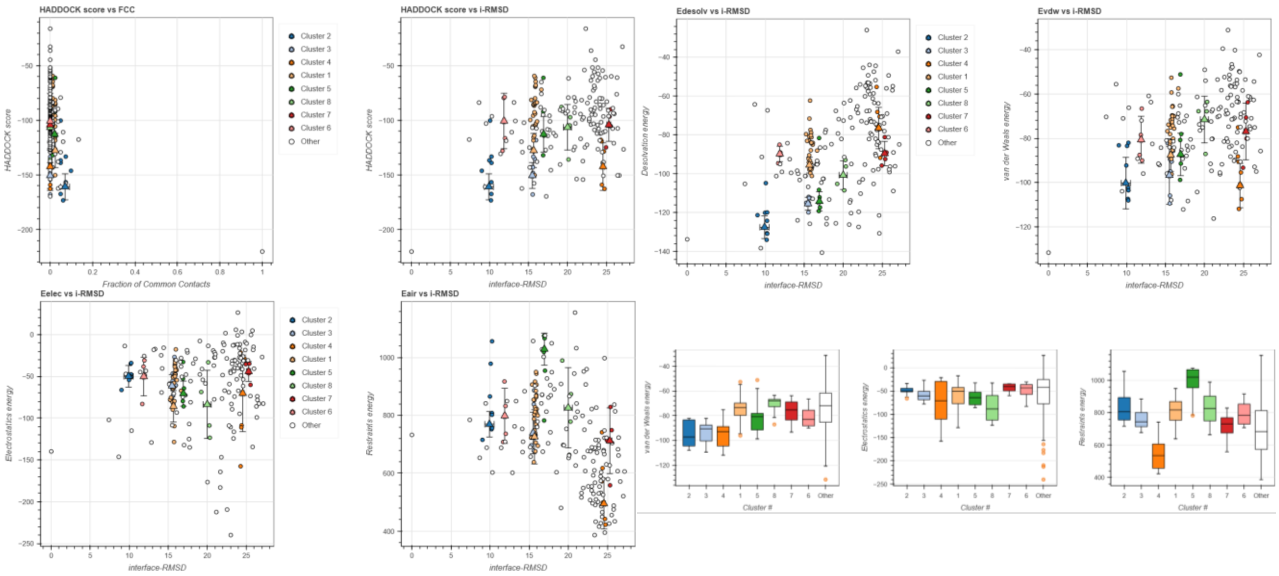

(Honorato *et al.*, 2024; Honorato *et al.*, 2021)

48

MSF- *Yersinia pestis* and BD12LC5 (HADDOCK)

Cluster 1

HADDOCK score	-163.8 +/- 14.5
Cluster size	15
RMSD from the overall lowest-energy structure	1.2 +/- 0.7
Van der Waals energy	-99.2 +/- 1.8
Electrostatic energy	-82.4 +/- 12.2
Desolvation energy	-121.2 +/- 5.2
Restraints violation energy	730.8 +/- 125.9
Buried Surface Area	2671.8 +/- 126.4
Z-Score	-1.8

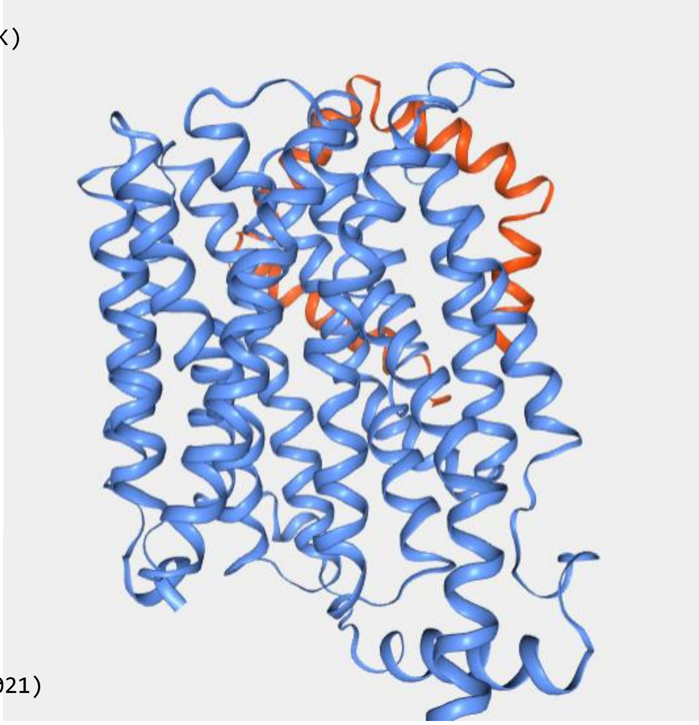

(Honorato *et al.*, 2024; Honorato *et al.*, 2021)

49

MSF- *Yersinia pestis* and BD12LC5 (HADDOCK)

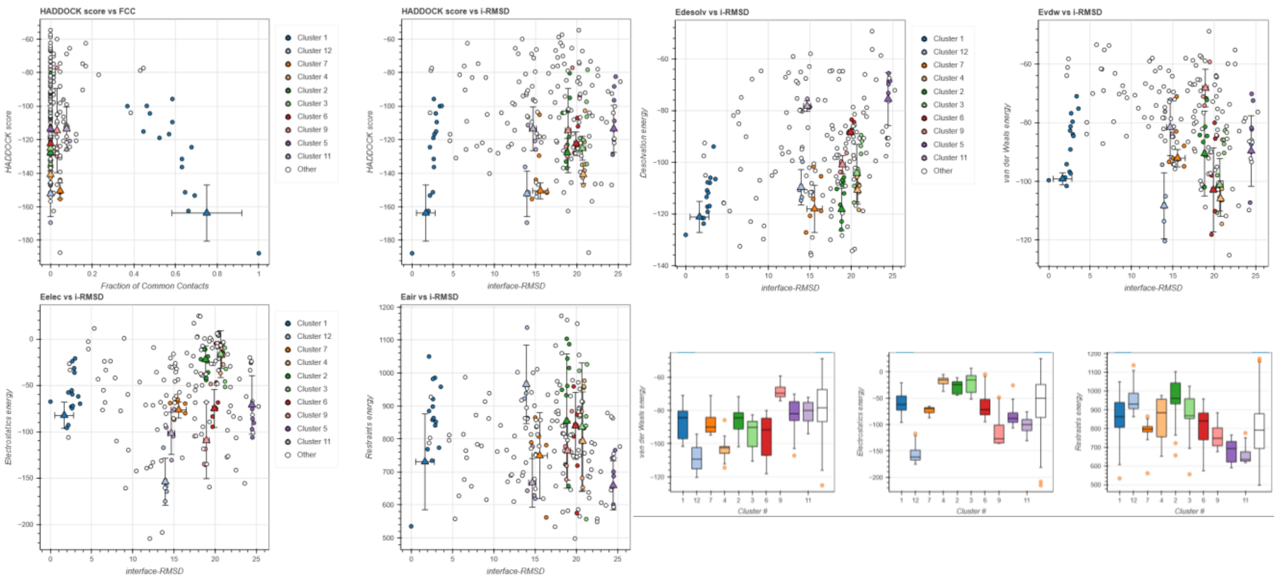

(Honorato *et al.*, 2024; Honorato *et al.*, 2021)

50

Supplementary material
File: Structural_Comparison_AlphaFold_vs_Original_Main_Manuscript_Models

The following figures show the molecular docking results for each treatment design and receptor. These are the original results, which can be compared with those generated using AlphaFold and HADDOCK. HADDOCK was used in this part of the manuscript revision because AlphaFold's output format is .CIF. This format is not compatible with all structural modeling tools, and converting it to PDB is very slow because it is done per atom. The structures used in this work are numerous and large. The differences between the molecular docking methods are not significant. In fact, as previously mentioned, the results are similar.

52

Molecular Docking

HEX loria (original)

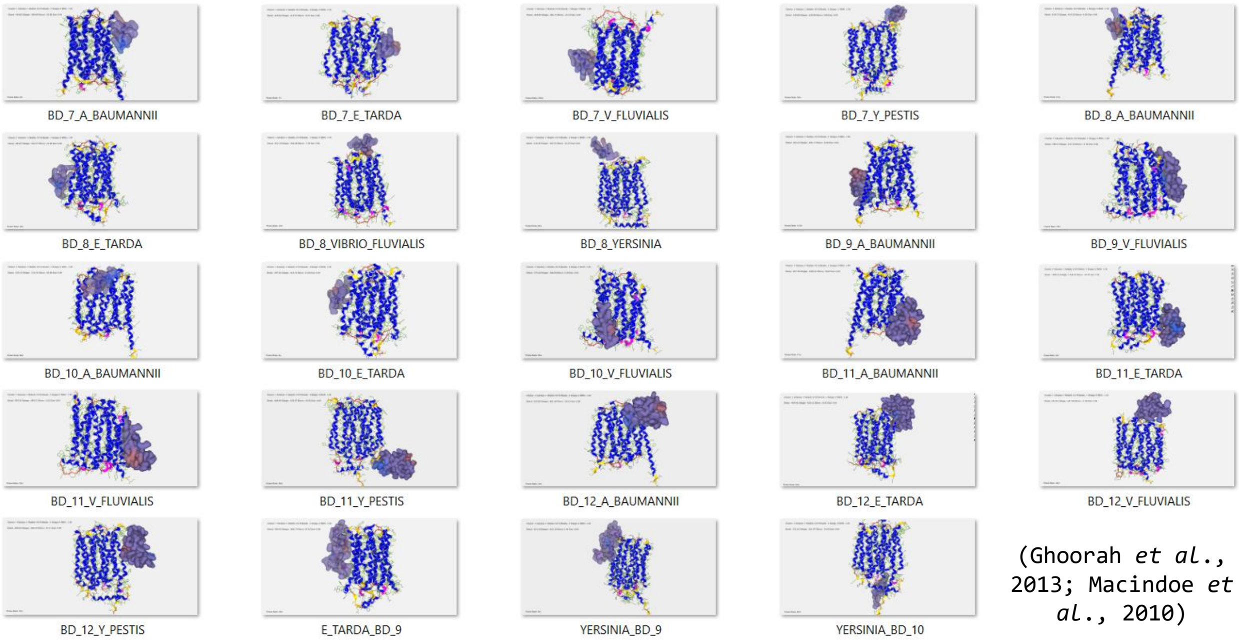

(Ghoorah *et al.*, 2013; Macindoe *et al.*, 2010)

53

Tools References:
File: Structural_Comparison_AlphaFold_vs_Original_Main_Manuscript_Models

Ye, B., Tian, W., Wang, B., & Liang, J. (2024). CASTpFold: Computed Atlas of Surface Topography of the universe of protein Folds. *Nucleic Acids Research*, *52*(W1), W194-W199. https://doi.org/10.1093/nar/gkae415

R.V. Honorato, M.E. Trellet, B. Jiménez-García1, J.J. Schaarschmidt, M. Giulini, V. Reys, P.I. Koukos, J.P.G.L.M. Rodrigues, E. Karaca, G.C.P. van Zundert, J. Roel-Touris, C.W. van Noort, Z. Jandová, A.S.J. Melquiond and A.M.J.J. Bonvin. "The HADDOCK2.4 web server: A leap forward in integrative modelling of biomolecular complexes" *Nature Prot.*, 19, 3219–3241 (2024).

R.V. Honorato, P.I. Koukos, B. Jimenez-Garcia, A. Tsaregorodtsev, M. Verlato, A. Giachetti, A. Rosato and A.M.J.J. Bonvin (2021). "Structural biology in the clouds: The WeNMR-EOSC Ecosystem." *Frontiers Mol. Biosci.*, **8**, fmolb.2021.729513.

Abramson, J., Adler, J., Dunger, J., Evans, R., Green, T., Pritzel, A., Ronneberger, O., Willmore, L., Ballard, A. J., Bambrick, J., Bodenstein, S. W., Evans, D. A., Hung, C.-C., O'Neill, M., Reiman, D., Tunyasuvunakool, K., Wu, Z., Žemgulytė, A., Arvaniti, E., … Jumper, J. M. (2024). Accurate structure prediction of biomolecular interactions with AlphaFold 3. *Nature*, *630*(8016), 493-500. https://doi.org/10.1038/s41586-024-07487-w

54
