# Supplementary material for: Identification of Opportunistic Pathogens on the Skin of Salamanders for Use as Molecular Targets of a De Novo Design of Multitarget Anti‐Bd Proteins
Source: Int J Microbiol. 2026 Apr 20;2026:5903624. doi: 10.1155/ijm/5903624 (PMC13093180; doi:10.1155/ijm/5903624)
Supplement: Supplementary file 1 — Supporting Information Additional supporting information can be found online in the Supporting Information section. [file IJM-2026-5903624-s001.zip › SUPPLEMENTAL-material-complement-figures-information.pdf]

SUPPLEMENTAL MATERIAL: Complement figures

Table 1. Identification of Opportunistic Pathogens on Salamander Skin. The following table
summarizes the results obtained from the 16S rRNA Internal Transcribed Spacer (ITS) PCR program for identifying pathogenic bacteria, performed using the raw FASTQ files from animals
of the genera *Ambystoma*, *Desmognathus*, *Eurycea*, and *Notophthalmus*. In the first column, we
can see the names of the bacteria; in the next, the number of reads that matched the sequence of
the bacteria to be detected and the percentage of those same reads.

Ambystoma_altamirani			Desmognathus_monticola			Eurycea_bislineata			Notophtalmus_viridicens		
Species	Match_num	Percentage	Species	Match_num	Percentage	Species	Match_num	Percentage	Species	Match_num	Percentage
Pseudomonas aeruginosa	4619	17.371	Vibrio fluvialis	5794	10.544	Vibrio fluvialis	8918	14.316	Vibrio fluvialis	6507	11.374
Edwardsiella tarda	2333	8.774	Pseudomonas aeruginosa	624	1.136	Pseudomonas aeruginosa	1477	2.371	Pseudomonas aeruginosa	1113	1.946
Aeromonas hydrophila	100	0.376	Vibrio vulnificus	268	0.488	Klebsiella pneumoniae	477	0.766	Klebsiella pneumoniae	308	0.538
Acinetobacter baumannii	28	0.105	Klebsiella pneumoniae	187	0.34	Vibrio vulnificus	360	0.578	Edwardsiella tarda	279	0.488
Klebsiella pneumoniae	28	0.105	Enterobacter cloacae	55	0.1	Edwardsiella tarda	343	0.551	Vibrio vulnificus	212	0.371
Bacillus cereus	22	0.083	Afipia broomeae	52	0.095	Enterobacter cloacae	97	0.156	Enterobacter cloacae	63	0.11
Yersinia pestis	22	0.083	Enterobacter aerogenes	41	0.075	Enterobacter aerogenes	67	0.108	Enterobacter aerogenes	47	0.082
Serratia marcescens	18	0.068	Bacillus anthracis	34	0.062	Enterobacter asburiae	52	0.083	Bacillus anthracis	33	0.058
Acinetobacter lwoffii	17	0.064	Brucella canis	28	0.051	Enterobacter ludwigii	45	0.072	Enterobacter asburiae	31	0.054
Yersinia pseudotuberculosis	13	0.049	Enterobacter kobei	20	0.036	Bacillus anthracis	31	0.05	Enterobacter ludwigii	24	0.042
Aeromonas punctata	13	0.049	Enterobacter ludwigii	20	0.036	Enterococcus faecium	25	0.04	Enterococcus faecalis	21	0.037
Plesiomonas shigelloides	7	0.026	Enterococcus faecium	19	0.035	Enterococcus faecalis	20	0.032	Enterococcus faecium	18	0.031
Yersinia enterocolitica	4	0.015	Enterobacter asburiae	18	0.033	Aeromonas hydrophila	19	0.03	Aeromonas hydrophila	17	0.03
Enterobacter aerogenes	4	0.015	Citrobacter freundii	17	0.031	Enterobacter hormaechei	19	0.03	Flavobacterium meningosepticum	13	0.023
Enterobacter cloacae	3	0.011	Edwardsiella tarda	17	0.031	Brucella canis	18	0.029	Enterobacter kobei	13	0.023
Afipia broomeae	3	0.011	Enterobacter Sakazakii	14	0.025	Brucella ovis	18	0.029	Brucella canis	11	0.019
Enterobacter hormaechei	1	0.004	Flavobacterium meningosepticum	13	0.024	Citrobacter freundii	17	0.027	Citrobacter freundii	10	0.017
Enterobacter mori	1	0.004	Enterobacter hormaechei	11	0.02	Klebsiella oxytoca	16	0.026	Enterobacter hormaechei	10	0.017
Afipia massiliensis	1	0.004	Afipia massiliensis	11	0.02	Flavobacterium meningosepticum	15	0.024	Brucella ovis	7	0.012
Serratia liquefaciens	1	0.004	Streptococcus gallolyticus	10	0.018	Serratia marcescens	15	0.024	Afipia broomeae	7	0.012
Enterobacter asburiae	1	0.004	Nocardia farcinica	10	0.018	Enterobacter kobei	14	0.022	Acinetobacter baumannii	6	0.01
Citrobacter freundii	1	0.004	Brucella ovis	10	0.018	Afipia broomeae	14	0.022	Yersinia pestis	5	0.009
Enterobacter ludwigii	1	0.004	Aeromonas hydrophila	9	0.016	Acinetobacter baumannii	12	0.019	Serratia marcescens	4	0.007
Afipia birgiae	1	0.004	Mycobacterium fortuitum	8	0.015	Yersinia pestis	11	0.018	Brucella neotomae	3	0.005
Enterobacter soli	1	0.004	Serratia marcescens	7	0.013	Acinetobacter lwoffii	6	0.01	Mycobacterium fortuitum	3	0.005

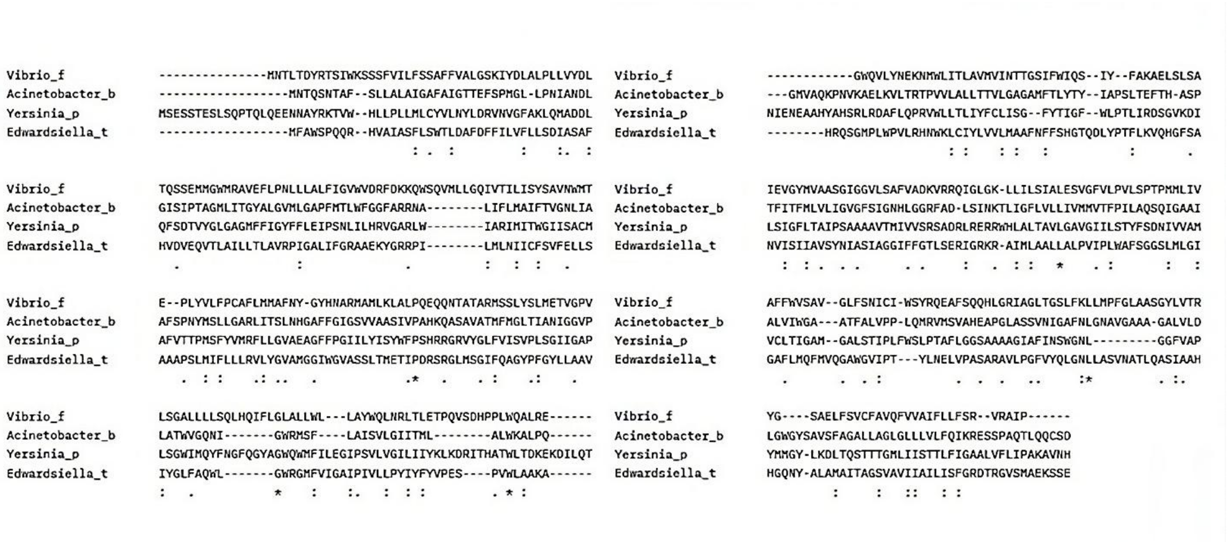

Figure 2. Multiple alignments. The results of the alignment performed for the MSF transmembrane protein of the four target microorganisms of the treatment to be designed are shown. Highly conserved regions can be observed in (*), moderately conserved in (:), and no significant conservation in (.). All four proteins have conserved sequences that may represent important functions in the protein across species. These regions can be used as potential targets for treatment by looking for their localization in the extracellular regions of the protein.

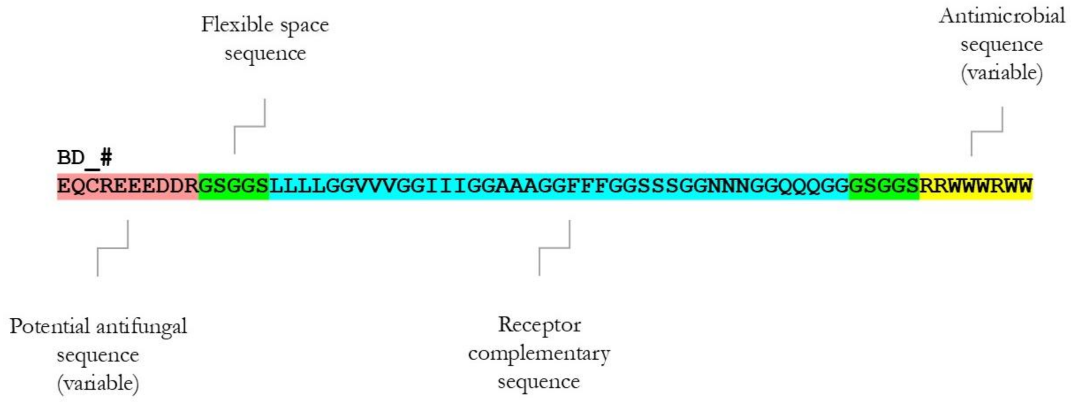

Figure 4. Diagram of antimicrobial model construction. It illustrates the construction of the sequence from various sections, including flexible spacers in gray and modified sequences with tested antimicrobial and antifungal effects, as indicated in green from databases. The 'complementary' sequence with optimal chemical properties for interaction with the studied amino acids of the outer regions of the MSF transmembrane protein is shown in orange. In addition, a short de novo sequence enriched in arginines and tryptophans, which are known to facilitate membrane interaction and have antimicrobial effects, is shown in cyan.

A comparison of all samples belonging to *Ambystoma altamirani* revealed a total of 12 pathogenic bacteria shared between samples of the same salamander species: *Yersinia pestis, Plesiomonas shigelloides, Pseudomona aeruginosa, Yersinia pseudotuberculosis, Edwardsiella tarda, Aeromonas hydrophila, Acinetobacter baumannii, Acinetobacter lwoffii, Enterobacter cloacae, Klebsiella pneumoniae, Aeromonas punctata, Yersinia enterocolitica*. The specific differences between *Ambystoma* samples are as follows: Sample 1) Six differential microorganisms: *Brucella ovis, Vibrio vulnificus, Mycobacterium bovis, Gardnerella vaginalis, Enterococcus faecium, Enterobacter mori*, and *Streptococcus vestibularis*. Sample 2) Only *Proteus vulgaris* and *Prevotella intermedia* were found. Sample 3) only *Streptococcus oralis* was found, and sample 4) *Shigella flexneri, Bacillus anthracis, Streptococcus mutans, Prevotella ruminicola, Citrobacter freundii, Shigella boydii* and *Prevotella melaninogenica.*

The comparison of samples of the genus *Desmognathus monticola* salamander revealed a total of 27 pathogenic bacteria common to all samples of the same salamander. These microorganisms include: *Brucella canis, Vibrio vulnificus, Bacillus anthracis, Enterobacter hormaechei, Afipia massiliensis, Yersinia pseudotuberculosis, and Bacillus cereus. Mycobacterium fortuitum, Enterococcus faecium, Aeromonas hydrophila, Vibrio fluvialis, Enterobacter aerogenes, Enterobacter cloacae, Enterococcus faecalis, Enterobacter asburiae, Yersinia pestis, Enterobacter kobei, Nocardia farcinica, Pseudomonas aeruginosa, Citrobacter freundii, Edwardsiella tarda, Afipia broomeae, Enterobacter ludwigii, Klebsiella oxytoca, Klebsiella pneumoniae, Brucella ovis and Enterobacter soli*. Some of the bacteria found as differential between samples are Sample 1) *Streptococcus oralis, Streptococcus mitis, Streptococcus peroris, Streptococcus entericus, Staphylococcus aureus, Pasteurella pneumotropica, Streptococcus australis, Bordetella bronchiseptica and Streptococcus pseudoporcinus*. In sample 2) *Afipia felis, Prevotella copri, Rickettsia conorii, Rickettsia massiliae, Streptococcus parasanguinis, Mycobacterium szulgai, Mycobacterium africanum* and *Mycobacterium ulcerans* were found. In samples 3, 4, and 5, only two differential bacteria were found in each sample: *Clostridium difficile and Morganella morganii; Vibrio cholerae and Amycolata autotrophica; and Actinomyces israelii* and *Actinomyces gerencseriae*, respectively.

In the case of *Eurycea*, a total of 29 pathogenic bacteria common to all samples of this salamander were identified. The list of identified bacteria is as follows: *Brucella canis, Vibrio vulnificus, Yersinia pestis, Enterobacter kobei, Pseudomonas aeruginosa, Bacillus anthracis, Enterobacter hormaechei, Yersinia pseudotuberculosis, Brucella neotomae, Citrobacter freundii, Edwardsiella tarda, Enterococcus faecium, Aeromonas hydrophila, Vibrio fluvialis, Flavobacterium meningosepticum. Streptococcus mitis. Streptococcus equinus*, *Enterobacter aerogenes, Serratia marcescens, Enterobacter ludwigii, Enterobacter cloacae, Klebsiella oxytoca, Enterococcus faecalis, Klebsiella pneumoniae, Aeromonas punctata, Brucella ovis, Enterobacter asburiae, Streptococcus suis and Yersinia enterocolitica*. Differential bacteria were found in both samples, a total of 8 in sample 1 and 14 in sample 2.

In the case of the genus *Notophthalmus*, a total of 21 opportunistic pathogens were identified in all samples corresponding to the same salamander species and belonging to the following species: *Brucella canis, Vibrio vulnificus, Bacillus anthracis, Enterobacter hormaechei, Enterococcus faecium, Aeromonas hydrophila, Vibrio fluvialis, Flavobacterium meningosepticum, Enterobacter aerogenes, Enterobacter cloacae, Enterococcus faecalis, Aeromonas punctata, Enterobacter asburiae, Yersinia pestis, Enterobacter kobei, Streptococcus parauberis, Citrobacter freundii, Acinetobacter baumannii, Acinetobacter lwoffii, Enterobacter ludwigii* and *Klebsiella pneumoniae*. Differentially, each sample showed 11, 3, 2, 1, and 7 pathogenic bacteria among samples from different salamanders of the same species (see complete list in Supplementary Material Table S1).

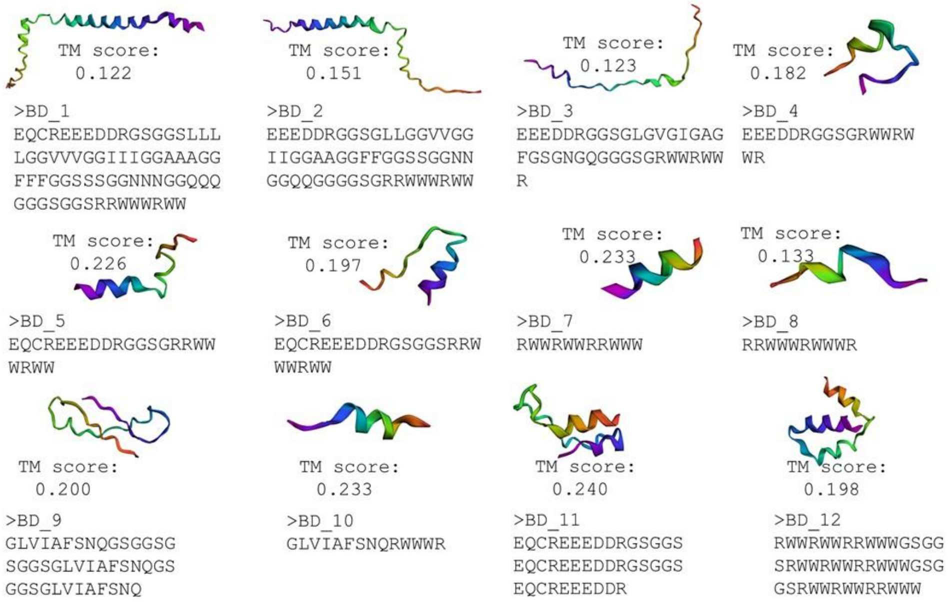

Figure 5. Generated antimicrobial models. The models generated from the constructed sequences are shown. In total, twelve proteins were designed and modeled with varying amino acid lengths and different sequence parts, each with predicted effects, including short or long flexible spacers, hinge amino acids, amino acid chains with modified antimicrobial or antifungal properties, and random tryptophan and arginine sequences. The modeling was performed to study the physical properties and possible interactions of these proteins with their molecular targets.

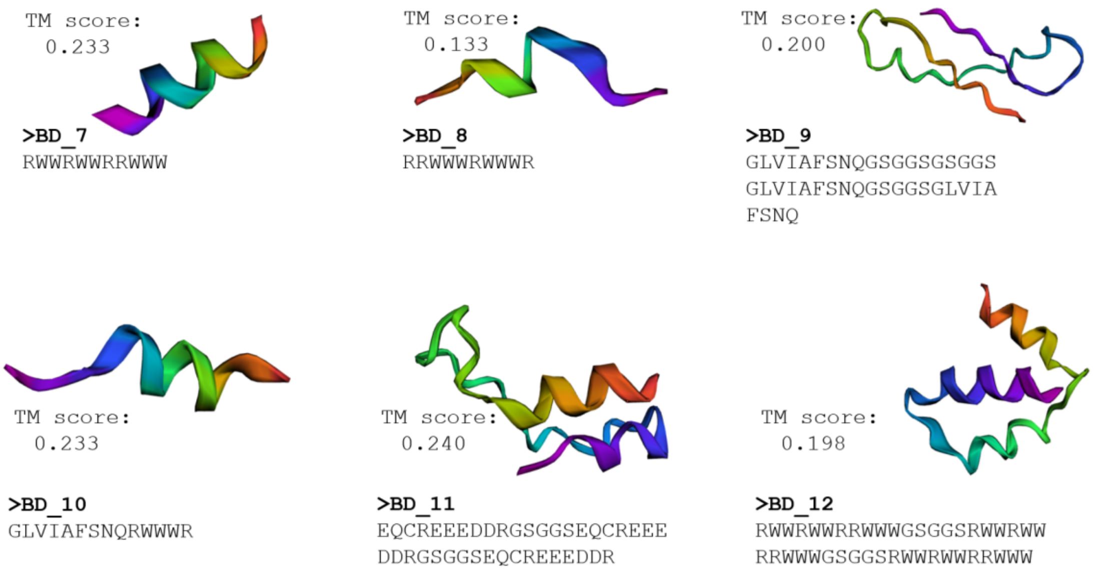

Figure 6. Models after the filter tests. The following figure illustrates the modeling of sequences that passed the above filters, along with their TM score and sequence.

Sequence	KAAAKWLLKIIKCCPCC
Prediction	CHHHHHHHHHHCCCCCC
Conf. Score	9189999998017899
	H:Helix; S:Strand; C:Coil

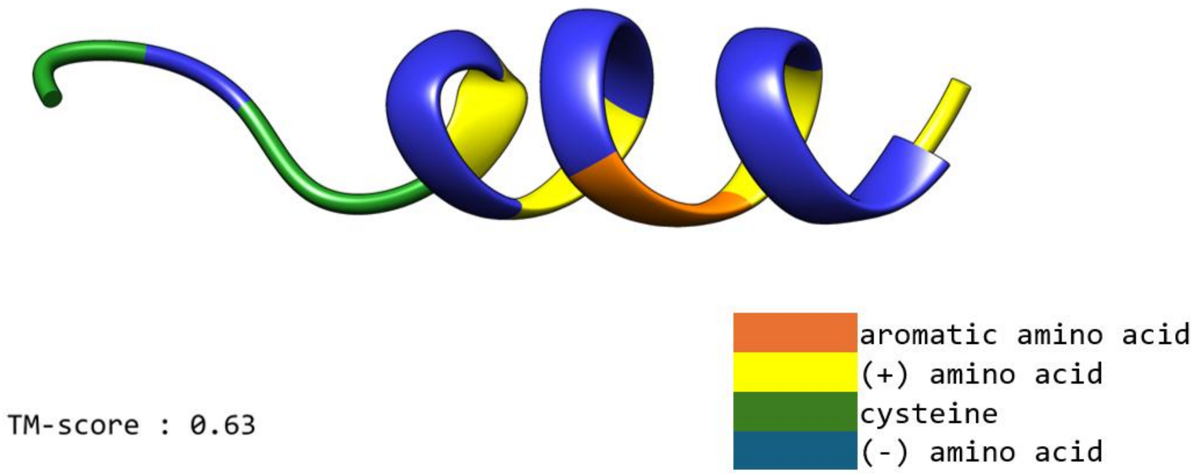

Figure 7. Anti-Bd model. The following figure shows the LC5 section, which was also constructed from the sequence following the procedure performed for the sequences with antimicrobial effects. It has different sections in yellow, orange, blue, and green. Each part ensures the stability of the peptide, and a specific part of the sequence ensures binding to both negatively and neutrally charged membranes.

sequence with membrane binding function (see Figures 9, 10, 11).

The structural models BD8LC5, BD9LC5, and BD11LC5 were subjected to the analysis. We found that the three proteins were cataloged by the program as BINDER (there is binding/interaction). They were able to locate the regions in the three proteins that probably interact with the membrane, and fortunately, in each protein, there is more than one region with this function, which is the expected result in all cases. In BD8LC5, the sequences RRWWWRWWWRGSGG, KAAAK, LKI, and Arginine #27 K had a positive prediction as BINDER (see Figure 9). The BD9LC5 protein has a censor region (amino acid serine #30), two K-membrane binding amino acids, arginine #51 and #55, and a motif with amino acids proline, cysteine, and cysteine #61 and #62 (see Figures 9, 10, 11). BD11LC5, the amino acids N #1 and W #25; the motifs C #4 and R #5; R #27 and R #28; P #67, C #68, C #69 and the RWWRRWWW There were three different models with the best results in each of the filters and tests used for their selection. These three models had to be tested for their potential affinity and interaction with the membrane-specific molecular targets, which were the proteins selected for each target: opportunistic bacteria or the *Bd* fungus. The HEX loria and Chimera programs were used for this part of the analysis. Those related to opportunistic bacteria are described in the supplementary material. The Chimera tool was primarily used for visualizing structures; HEX loria is a crucial program for molecular docking, which enables us to determine if a complete protein can interact with another protein, in this case, the membrane proteins that will be labeled as receptors. The MFS receptor proteins of *Vibrio fluvialis*, *Acinetobacter baumannii*, *Edwardsiella tarda*, and *Yersinia pestis* were tested with the engineered proteins BD9LC5, BD8LC5, and BD12LC5, respectively.

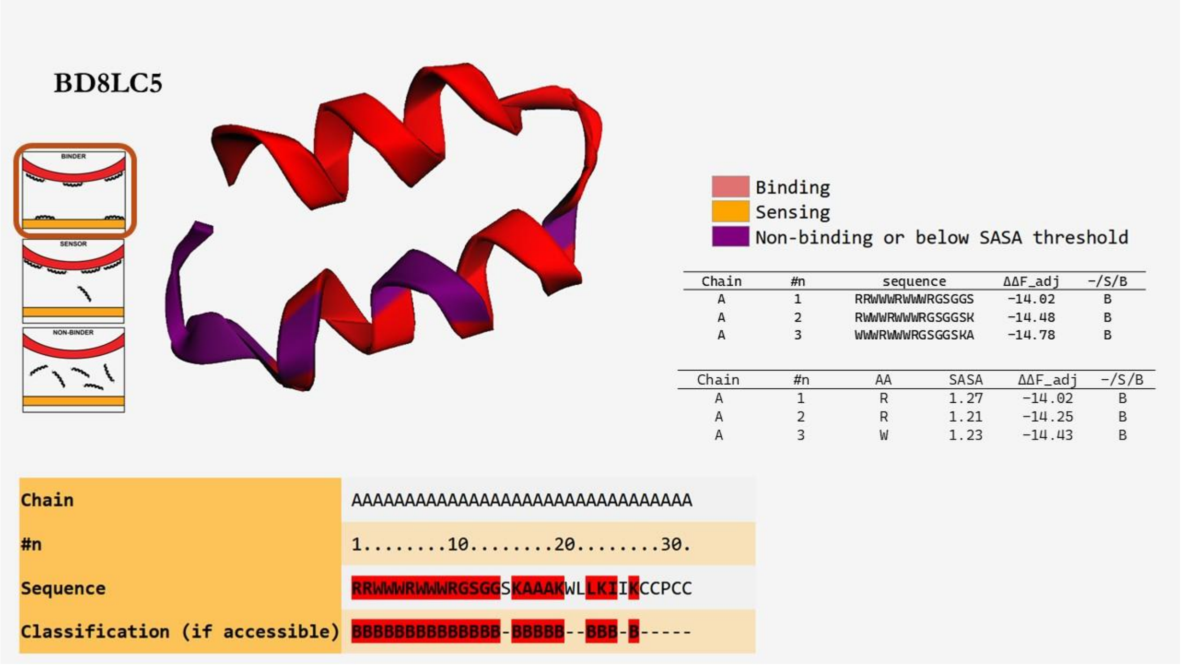

Figure 9. Membrane binding assay with the structural models 1. The following figure illustrates the negatively charged membrane binding test conducted using the PMIpred program, which predicts the sequence segments and amino acids that bind to negatively charged or neutrally charged membranes. Results are presented for negatively charged membranes, although tests were also performed on neutral membranes, yielding similar results. Predicted binding regions are shown in red in both the protein model and the sequence. Also shown are the first rows of the results Table S2, which show the calculated energy values. The threshold used for these tests was 0.8. Positive results were obtained in all cases (The complete list is available at Table S2).

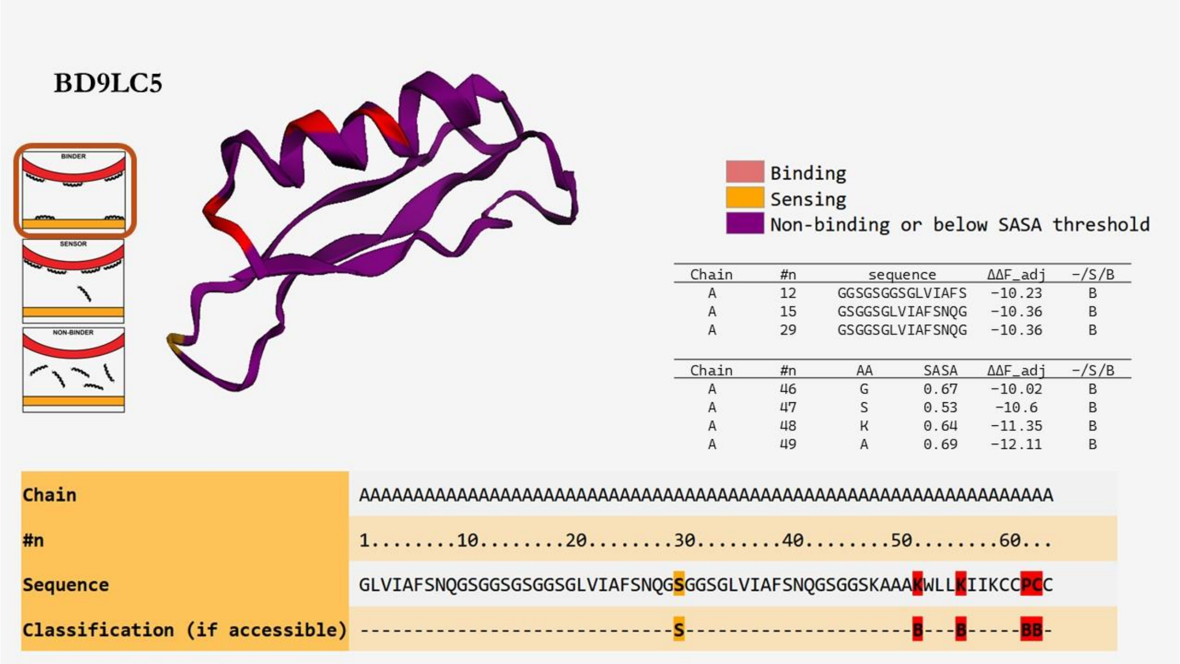

Figure 10. Membrane binding assay with the structural models 2. The following figure illustrates the negatively charged membrane binding test conducted using the PMIpred program, which predicts the sequence segments and amino acids that bind to negatively charged or neutrally charged membranes. Results are presented for negatively charged membranes, although tests were also performed on neutral membranes, yielding similar results. Predicted binding regions are shown in red in both the protein model and the sequence. Also shown are the first rows of the results Table S2, which show the calculated energy values. The threshold used for these tests was 0.8. Positive results were obtained in all cases (The complete list is available at Table S2).

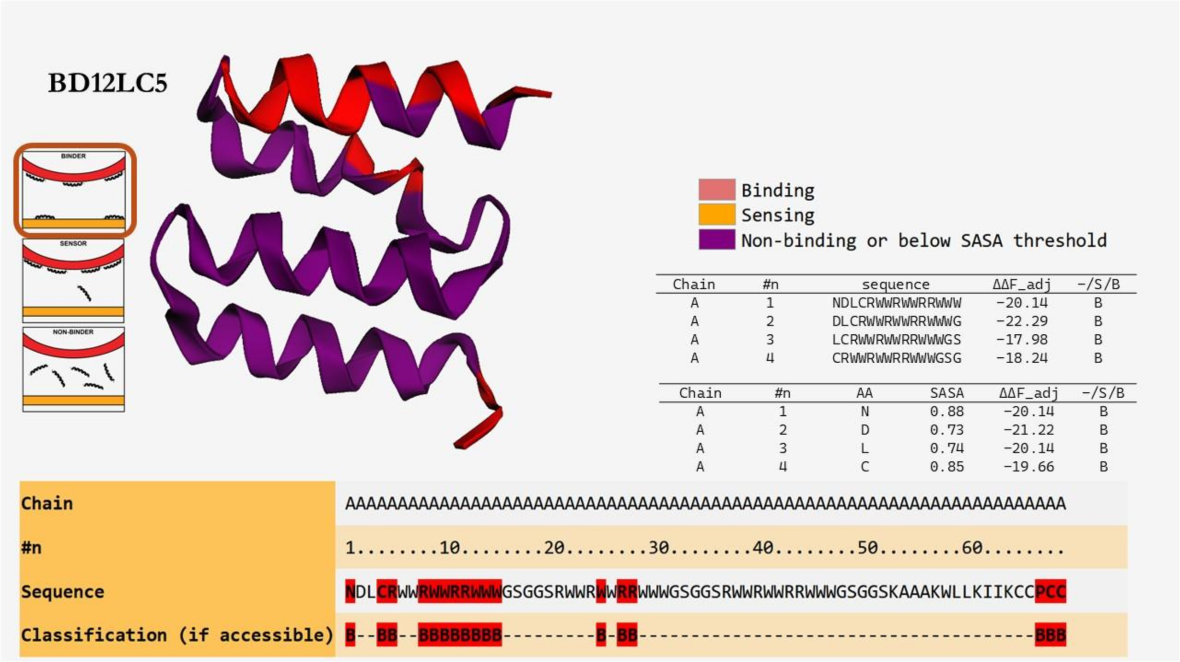

Figure 11. Membrane binding assay with the structural models 3. The following figure illustrates the negatively charged membrane binding test conducted using the PMIpred program, which predicts the sequence segments and amino acids that bind to negatively charged or neutrally charged membranes. Results are presented for negatively charged membranes, although tests were also performed on neutral membranes, yielding similar results. Predicted binding regions are shown in red in both the protein model and the sequence. Also shown are the first rows of the results Table S2, which show the calculated energy values. The threshold used for these tests was 0.8. Positive results were obtained in all cases (The complete list is available at Table S2).

Although currently available bioinformatics tools are powerful for protein design, identifying protein-protein interactions, identifying opportunistic pathogens within the microbiota, and many other tasks of interest, they have certain limitations. To date, the fungus-bacteria interactions that may occur in this *Bd* salamander or *Bd* amphibian model have not been studied; instead, other interactions have been tested in various models. Understanding these interactions in this study model would bring us even closer to a more specific and effective treatment. Additionally, remember that in vitro testing is always necessary for any treatment design. The next step would be to refine the designs and perform *in vitro* testing to ensure their efficacy while also testing the safety of their future use. Designing this type of treatment is just the beginning. With the emergence of new bioinformatics tools in the coming years, designs could be further improved, interactions between the treatment and its target could be identified, and additional tests could be performed in addition to those conducted here. This will lead to much faster and more efficient production of treatments and even vaccines.

Different opportunistic pathogens were identified for all animals in the generated database. Each of the animals presented a different number of pathogenic bacteria identified on their skin; even among animals of the same species, different numbers of bacteria and different species were found. Some of these differences may be due to contamination from various sources that can occur during the different stages of sampling, as well as to differences in the contamination of the habitat of these salamanders and whether their final stage of development is aquatic or terrestrial. It also depends on the level of urbanization within the sampling localities; these are aspects that could not be controlled in this project. The presence of various opportunistic bacteria in the environment has been documented, and this is where a large proportion of the bacteria identified were obtained from animals in the different studies analyzed here [110, 111].

Some salamanders tend to select appropriate microorganisms that are not abundant in the environment in which they live. It is likely that the presence of specific opportunistic pathogens in *Notophthalmus viridescens* is due to this cause [110, 111]. Most of the pathogens identified are human opportunistic pathogens, and no cases have been reported in amphibians. However, some bacteria have been documented, such as *Mycobacterium gordonae*, a human pathogen that causes disease in *Xenopus tropicalis* [112].

To date, antibiotic treatment of *Bd-infected* amphibians has been shown to induce significant changes in the gut microbiota of the animals. Surprisingly, they do not completely alter the diversity of microorganisms present in the skin. These changes indicate that some treatments should be evaluated in terms of their therapeutic benefits versus the side effects they may

cause [113]. Antibiotic treatments may facilitate the transfer of antibiotic-resistance genes, leading to a more severe state of dysbiosis and the decline of probiotic microorganisms [113-116].

Dysbiosis in the gut microbiota has been studied about the disruption of synergies between microorganisms that can initiate or exacerbate disease in animals [117]. It has also been demonstrated that within the intestinal microbial dysbiosis of amphibians, specific pathogenic bacterial genera, such as *Aeromonas* and *Plesiomonas*, are associated with an increase in lesions and inflammation in Xenopus laevis with ulcerative colitis, which exacerbates the disease and damages the intestinal mucosa [116-118]. Therefore, the increase or presence of some pathogen genera in the microbiota may increase the impact of an initial disease. However, these studies have not been carried out on *Bd* and the presence of opportunistic bacteria during infection, but it is known that the treatment of intestinal diseases with antibiotics is capable of altering the skin microbiota [50, 119, 120]. It can also increase the susceptibility of animals to infection [115]. Therefore, it is essential to have treatment alternatives, including the design, search, development, and improvement of antimicrobials, which reduce the unwanted effects of treatment. This is because, during their construction, it is about avoiding and taking into account each aspect.

The presence of some opportunistic pathogens, such as the genus *Acinetobacter*, in frogs like *Paa spinosa* increases their susceptibility to diseases caused by pathogens [118]. There is also evidence that animals with a higher microbiota, composed of opportunistic microorganisms, are at a greater risk of infection [118.]

Pathogens have also been found in healthy animals [119] and mice [120], as have the results of this article. It is, therefore, not surprising to think that the presence of opportunistic microorganisms in low proportions does not harm the animals, which is why they maintain a "healthy" state [120, 121]. The goal of computational treatments is to restore balance in the microbiota of sick animals, as the infection induces dysbiosis, and a significant portion of the bacteria responsible for maintaining health and supporting the immune system has been lost. Many bacteria classified as opportunistic, commensal, or facultative pathogens are not capable of causing infection under normal conditions. However, they can make the host sick when the microenvironment in the tissues is suitable for their development [122]. It has been studied that these microorganisms can favor the colonization and development of pathogens [123, 124]. An example of this is the bacteria of the genus *Lactobacillus*, which are regularly associated with gut health and are, in fact, part of various products on the market [123, 125].

However, these same bacteria are also capable of causing bacteremia in patients with liver abscesses and *Clostridium difficile* infection, and *Lactobacillus* supplementation is recommended [123, 125]. The high abundance of this bacterium has also been associated with patients with gastric cancer [123, 125]. One of the mechanisms studied so far is that the genus *Lactobacillus* can increase its gastrointestinal growth due to its tolerance to environments with acidic pH, making patients who have problems with pH-regulating pumps in gastric tissue susceptible to infection by this bacterium [126, 130]. More studies are needed on the microbiota and their interactions in the skin of amphibians, as it is currently only possible to make assumptions about their behavior in these animals and in animals with *Bd*. Just as the absence of gastrointestinal disease is associated with good nutrition, stress control,

and non-exposure to chemicals such as antibiotics [126], the absence of *Bd* in amphibians is associated with good nutrition, stress control, and non-exposure to chemicals such as antibiotics; in the case of amphibians, poor nutrition, pollution of water and soil, the continuous stress caused by these pollutants and the continuous warming of the planet, and possibly poor nutrition because the food sources of these animals can also decrease in quality due to the above factors, likely, many of the opportunistic pathogenic bacteria on the skin of amphibians can cause infection associated with *Bd*, but more studies are needed in this regard.
