# Supplementary material for: Identification of Opportunistic Pathogens on the Skin of Salamanders for Use as Molecular Targets of a De Novo Design of Multitarget Anti‐Bd Proteins
Source: Int J Microbiol. 2026 Apr 20;2026:5903624. doi: 10.1155/ijm/5903624 (PMC13093180; doi:10.1155/ijm/5903624)
Supplement: Supplementary file 1 — Supporting Information Additional supporting information can be found online in the Supporting Information section. [file IJM-2026-5903624-s001.zip › Supplementary_material_Figures_S1_S2(1)(1)(2).docx]

**SUPPLEMENTARY MATERIAL**

To construct the LC5 model, the same steps as presented for BD8, BD11 and BD12 were followed, as shown in the methodology of the manuscript. Several sequences were constructed with ideal characteristics to interact with a specific molecular target present in the membrane of *Bd* cells. These features are mainly amino acids known to have chemical interactions. After constructing the sequences (twelve in total), we proceeded to select those that fulfilled the function of interest. To demonstrate this point, we searched for molecular targets homologous to an Na/H+ transporter in other microorganisms using BLAST. Finally, a docking test was performed, the results of which are shown in Figures S1 and S2.


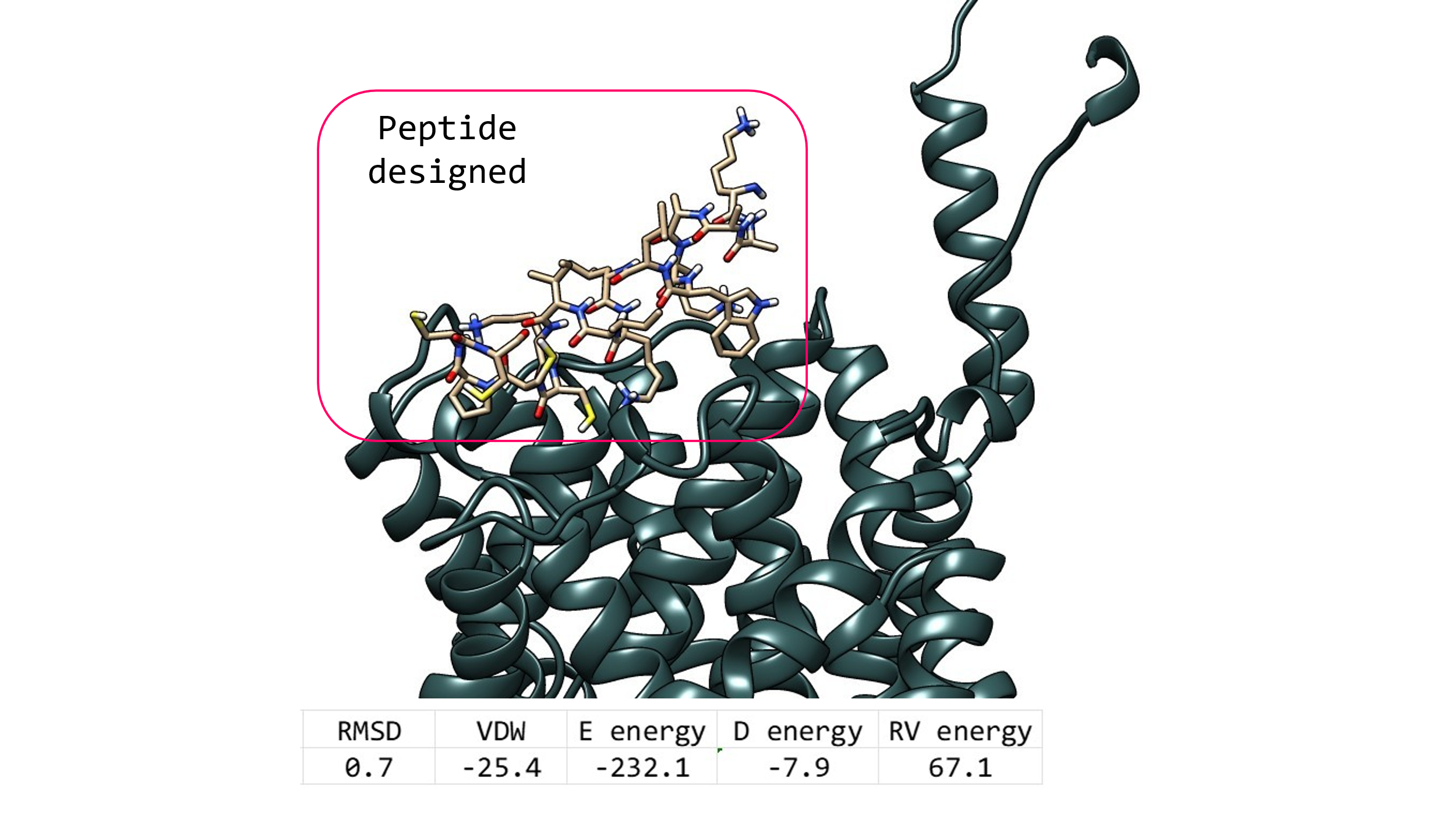


**Figure 1S. LC5 docking.** The results of the docking performed with the model of the designed protein LC5 and the Na/H+ transporter present in the *Bd* membrane are shown (These results have been described in accepted article JoVE: Villarreal, J. R. & Álvarez-Martínez, 2025).

The distances between the designed protein and the transporter present in *Bd* were also measured to infer the range of attraction and interaction that both proteins might have. The results of the distance measurements in the Chimera program are shown in Figure S2.


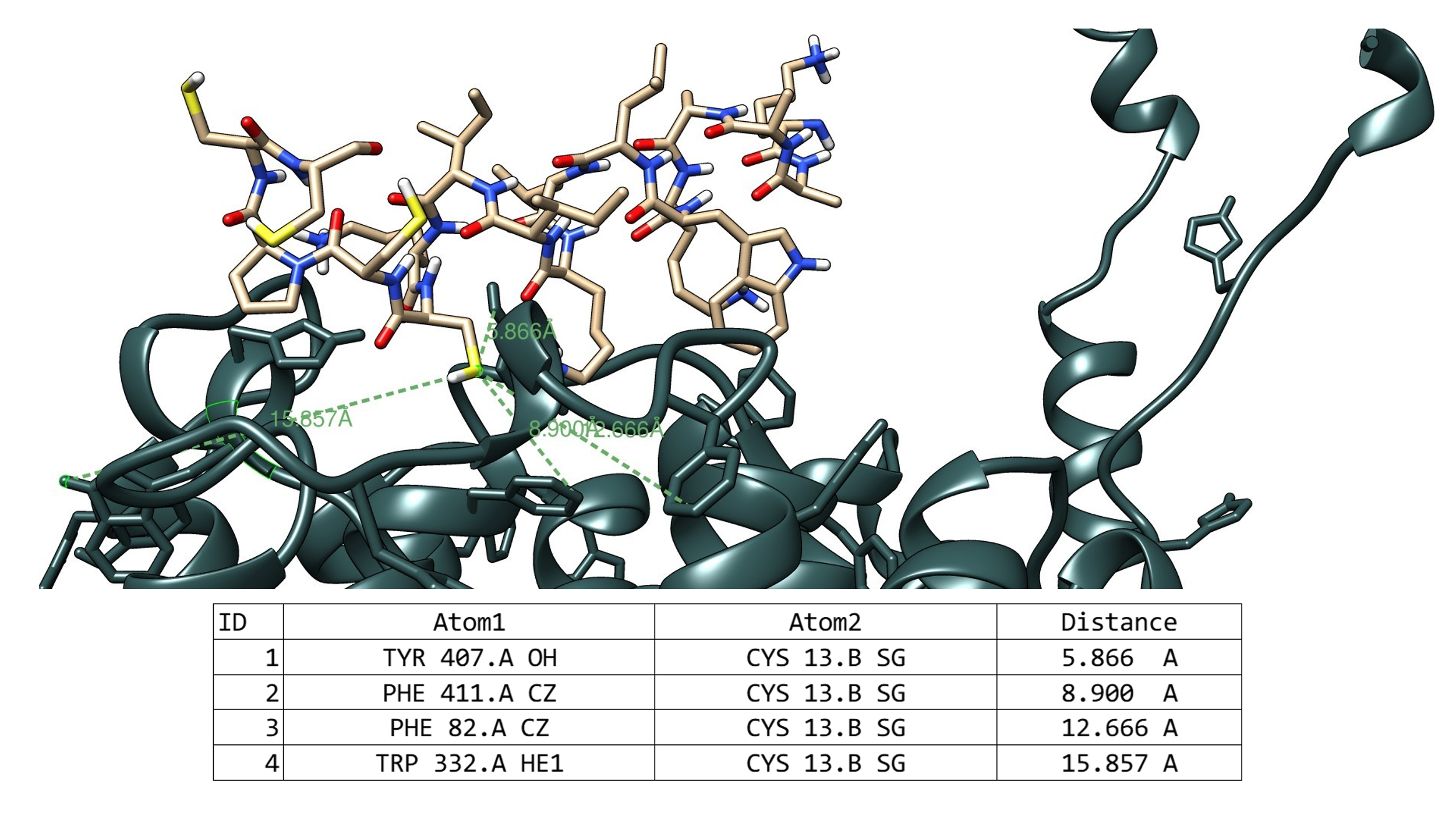


**Figure 2S. Measurement of distances.** A table and figure are shown corresponding to the measurement of the distances between the transporter and the designed protein. We can see that some of the amino acids placed in the design for the interaction of the two proteins have a certain distance in angstroms (These results have been described in accepted article JoVE: Villarreal, J. R. & Álvarez-Martínez, 2025).

**Reference**

When published, the Information on the article will be provided in this section.

**PRIOR APPOINTMENT:**
Villarreal, J. R., Álvarez-Martínez, R. Application of I TASSER, trRosetta, UCSF Chimera, HADDOCK server, and HEX loria for *De Novo* and *In Silico* Design of Proteins. *J. Vis. Exp.* (Pending Publication), e68003, In-press (2025).
